# Supplementary material for: Elevated ubiquitin phosphorylation by PINK1 contributes to proteasomal impairment and promotes neurodegeneration
Source: eLife. 2025 Jul 31;14:RP103945. doi: 10.7554/eLife.103945 (PMC12313235; doi:10.7554/eLife.103945)
Supplement: Figure 1—source data 1. [file elife-103945-fig1-data1.docx]

**Figure 1—source data 1. Clinical and pathological characteristics of brain donors.**

| **Donor** | **NBB/CBB number** | **Diagnosis** | **Age at death** | **Sex** | **Brain weight (g)** | **PMD (hrs:min)** | **Amyloid** | **Brain region** | **Neuropathological examination** | **Source** |
| --- | --- | --- | --- | --- | --- | --- | --- | --- | --- | --- |
| A | 1997-063 | Alzheimer’s Disease | 75 | F | 995 | 05:40 | B | CG |  | NBB |
| B | 2007-086 | Alzheimer’s Disease | 71 | M | 1362 | 05:25 | B | CG |  | NBB |
| C | 2019CBB050 | Acute leukemia, secondary malignant bone tumors, renal malignant tumors, pulmonary infection, heart failure | 70 | F | 1272 | 06:20 | O | CG | ND | CBB |
| D | 2022CBB083 | Breast cancer with multiple metastases; acute renal failure; hypertension | 72 | M | 1409 | 13:48 | O | CG | ND | CBB |

1. The amyloid indicates Aβ, which was absent with “O” and moderate with “B”.

2. Abbreviation, F is female; M is male; PMD is postmortem delay; CG is cingulate gyrus.

3. ND represents no obvious abnormal detected.

4. NBB represents the Netherlands Brain Bank, Netherland.

5. CBB represents the National Health and Disease Human Brain Tissue Resource Center, China.
