# Supplementary figures and images for: Elevated ubiquitin phosphorylation by PINK1 contributes to proteasomal impairment and promotes neurodegeneration (part 1 of 2)
Source: eLife. 2025 Jul 31;14:RP103945. doi: 10.7554/eLife.103945 (PMC12313235; doi:10.7554/eLife.103945)

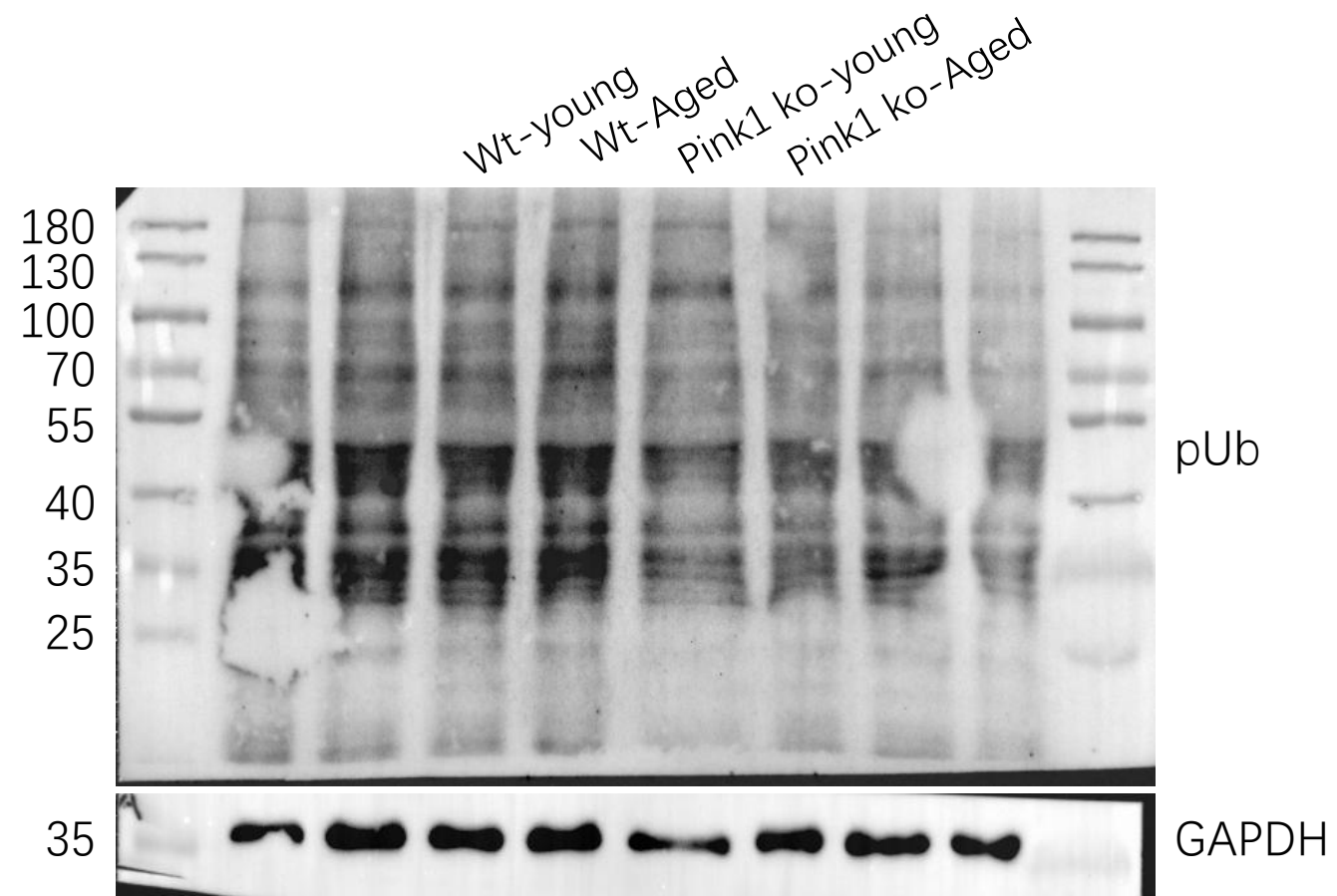

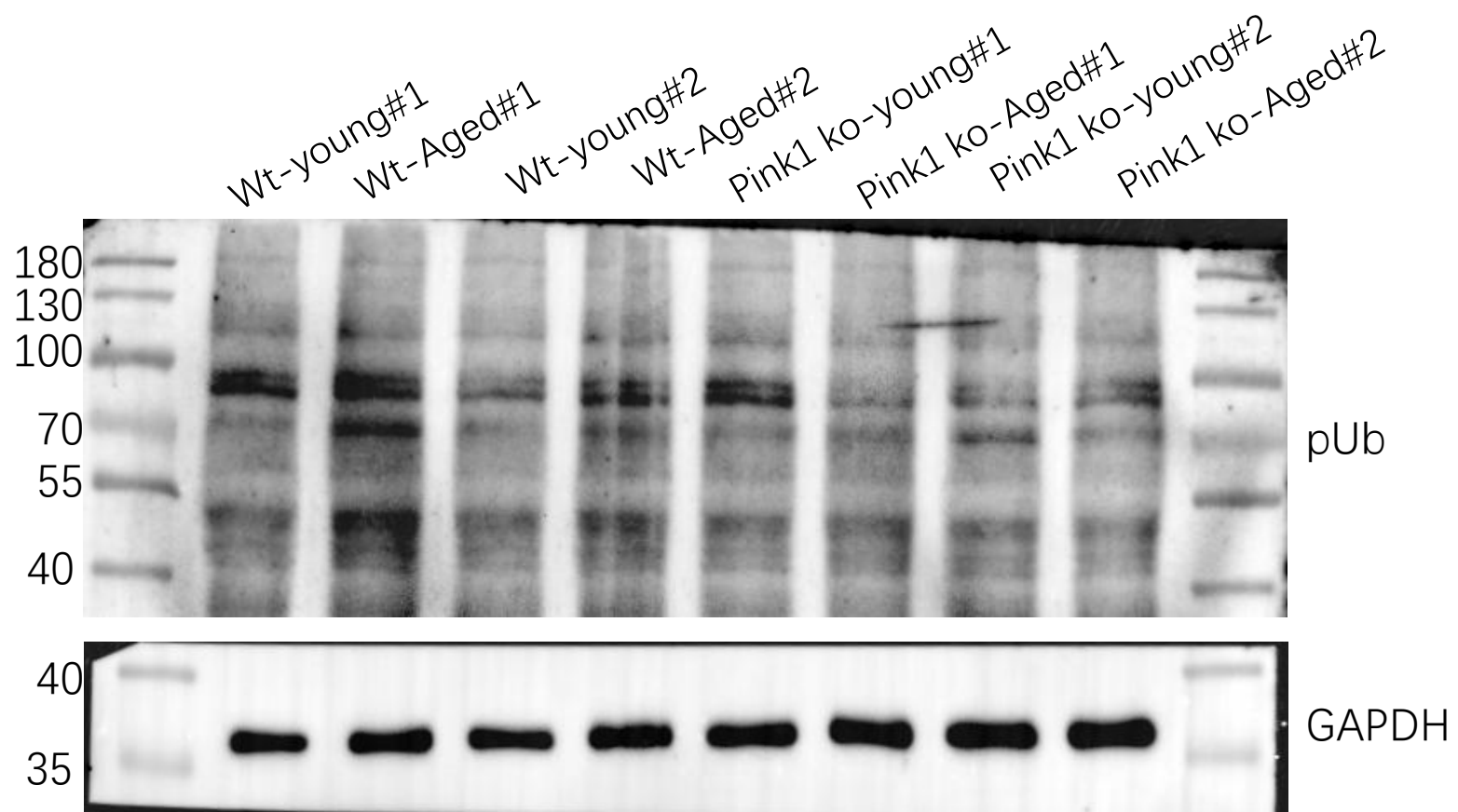

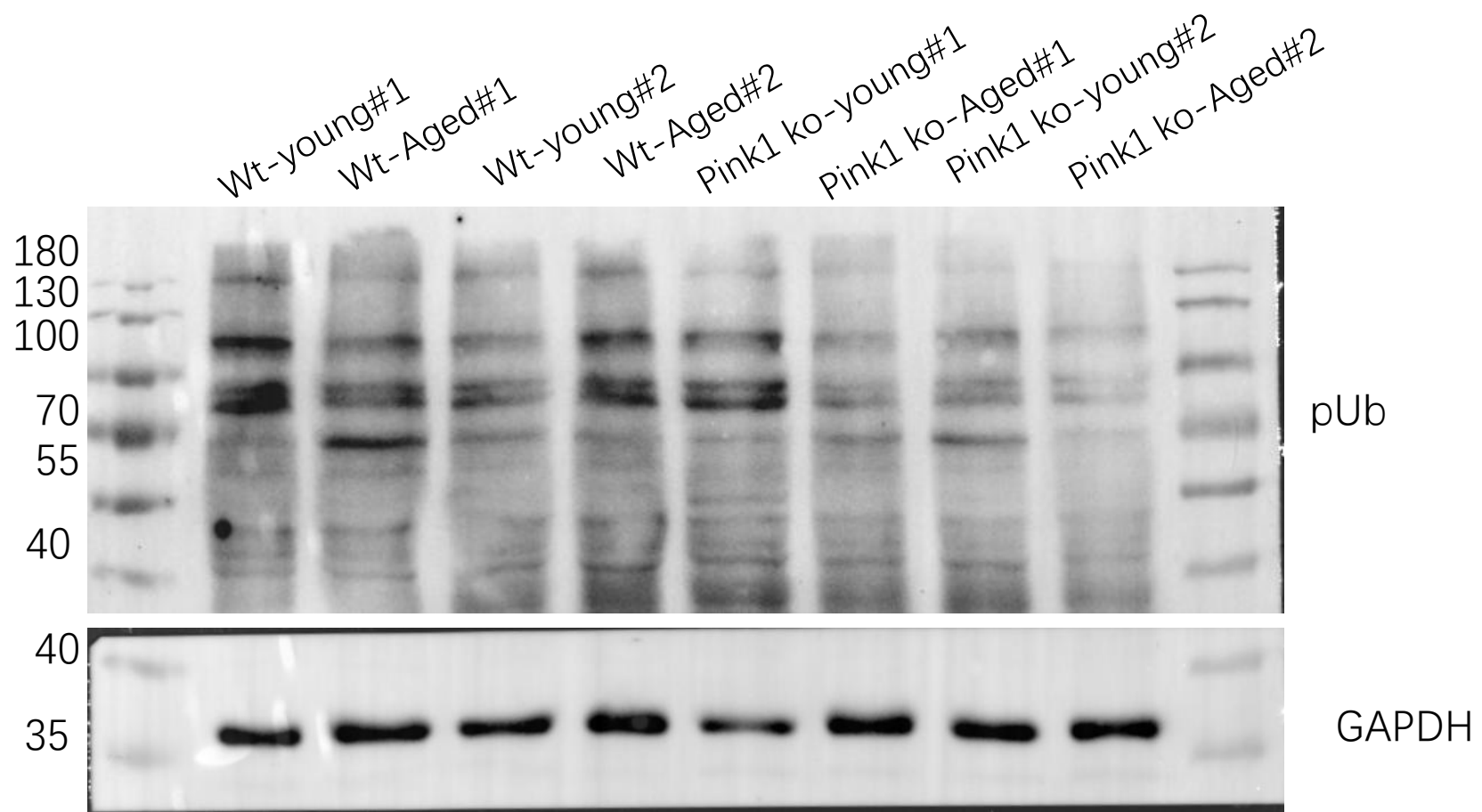

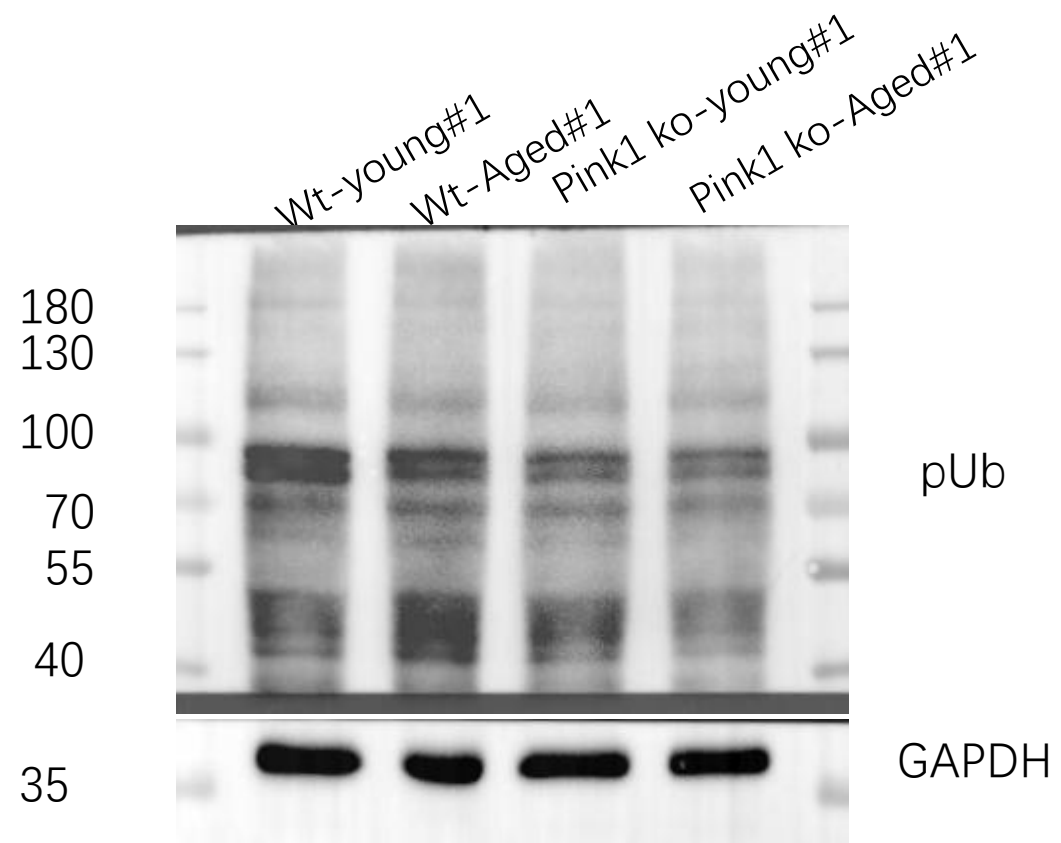

Supplement: Figure 1—source data 2. [file elife-103945-fig1-data2.pdf]

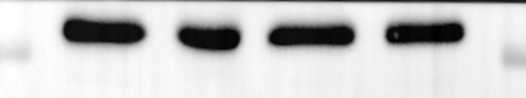

Supplement: Figure 1—source data 3. [file elife-103945-fig1-data3.zip › GAPDH-2.tif]

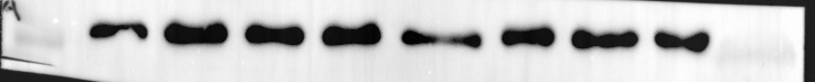

Supplement: Figure 1—source data 3. [file elife-103945-fig1-data3.zip › GAPDH-3.tif]

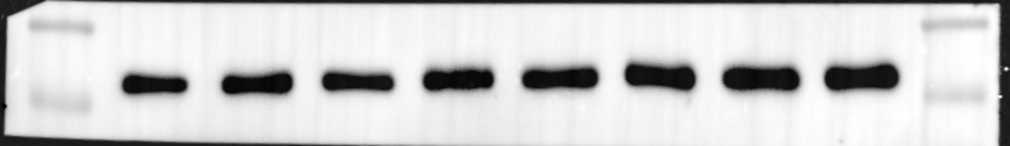

Supplement: Figure 1—source data 3. [file elife-103945-fig1-data3.zip › GAPDH-4.tif]

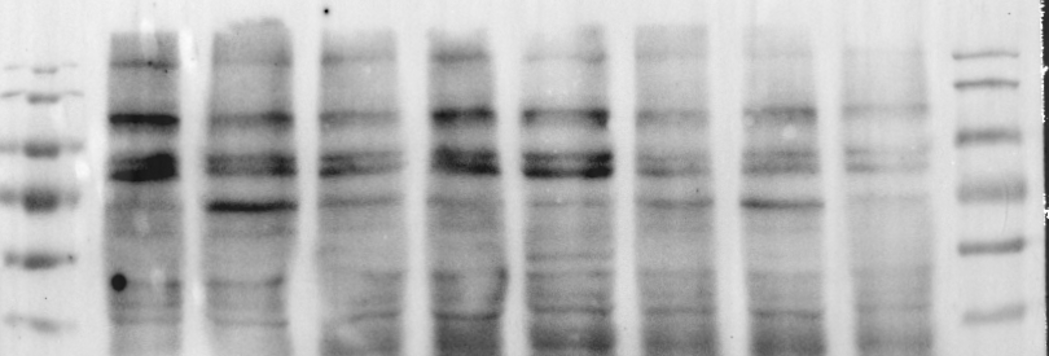

Supplement: Figure 1—source data 3. [file elife-103945-fig1-data3.zip › pUb-1.tif]

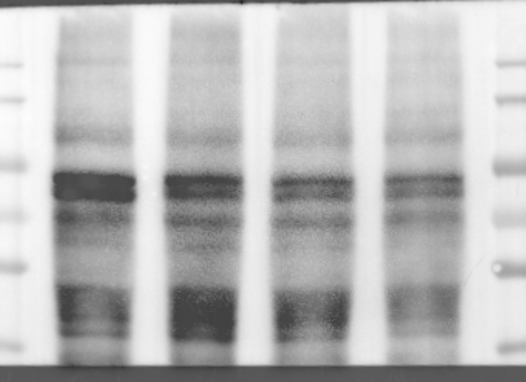

Supplement: Figure 1—source data 3. [file elife-103945-fig1-data3.zip › pUb-2.tif]

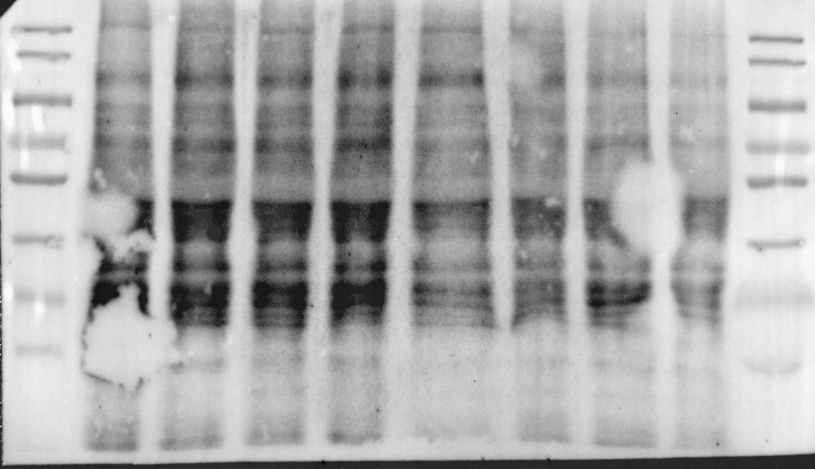

Supplement: Figure 1—source data 3. [file elife-103945-fig1-data3.zip › pUb-3.tif]

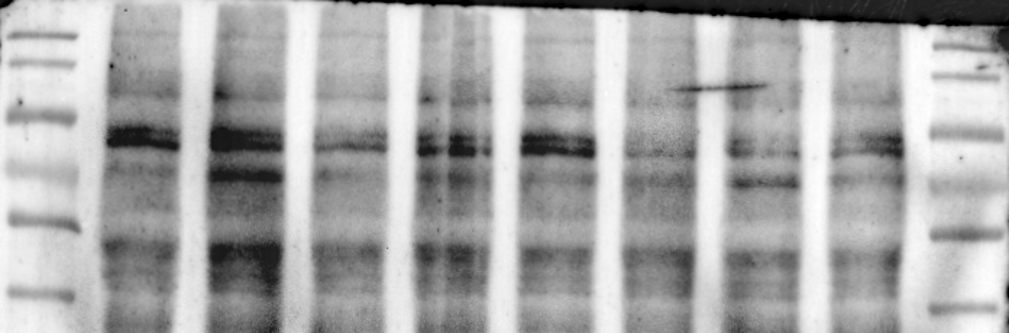

Supplement: Figure 1—source data 3. [file elife-103945-fig1-data3.zip › pUb-4.tif]

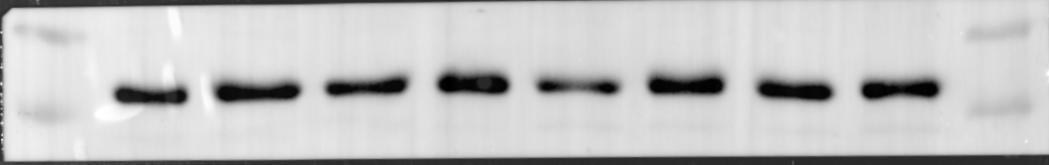

Supplement: Figure 1—source data 3. [file elife-103945-fig1-data3.zip › GAPDH-1.tif]

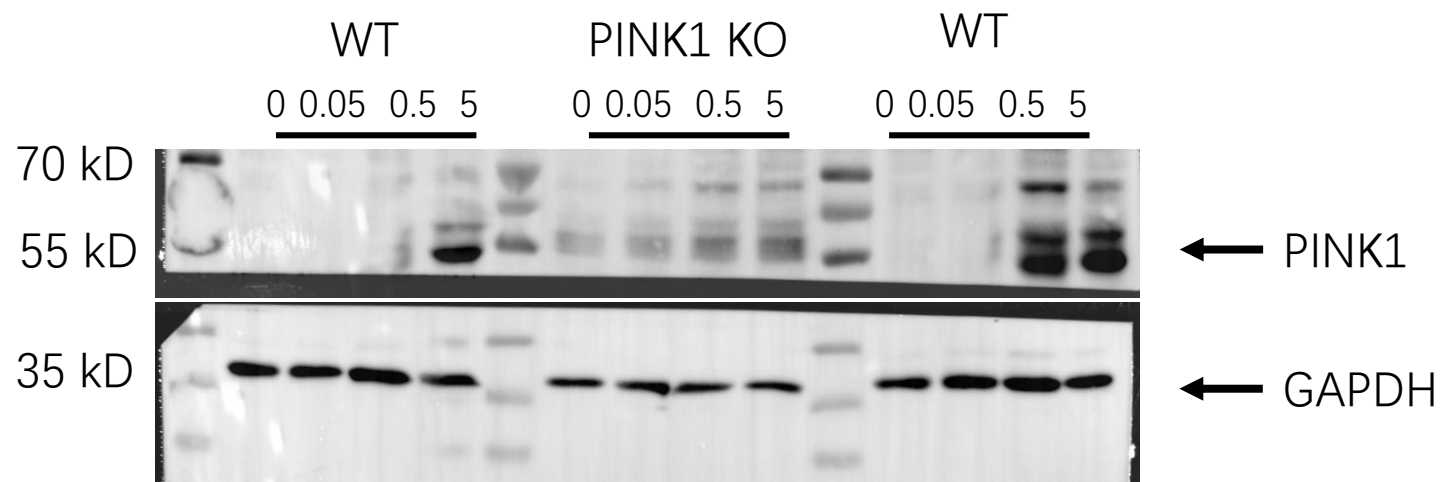

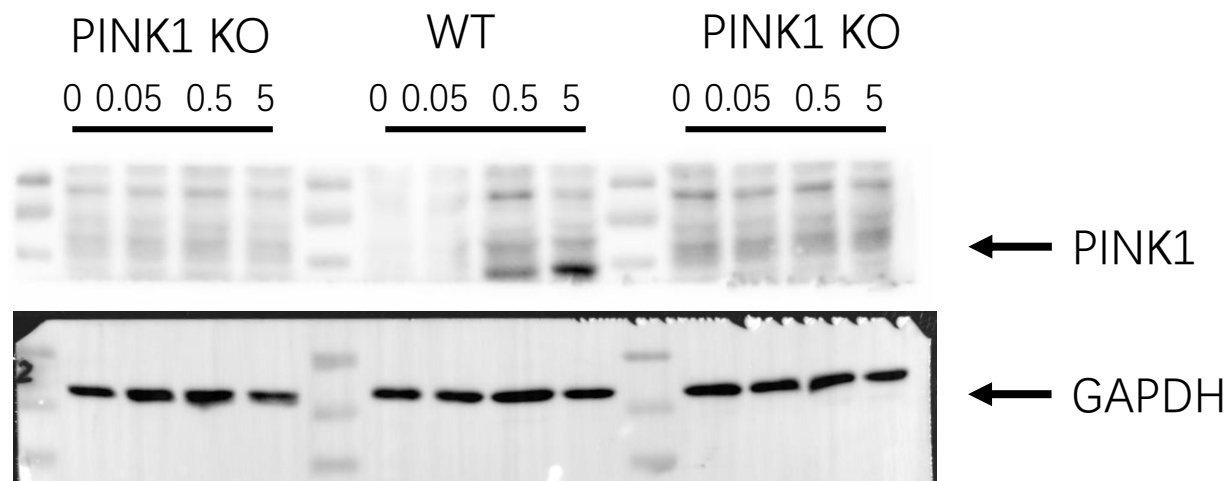

Supplement: Figure 2—source data 1. [file elife-103945-fig2-data1.pdf]

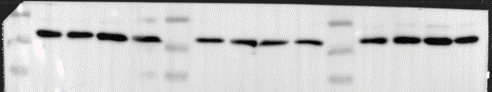

Supplement: Figure 2—source data 2. [file elife-103945-fig2-data2.zip › GAPDH-1.png]

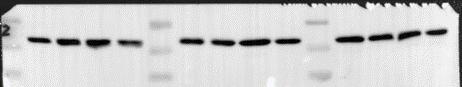

Supplement: Figure 2—source data 2. [file elife-103945-fig2-data2.zip › GAPDH-2.png]

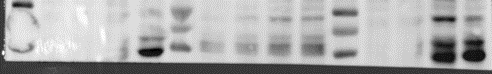

Supplement: Figure 2—source data 2. [file elife-103945-fig2-data2.zip › PINK1-1.png]

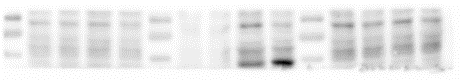

Supplement: Figure 2—source data 2. [file elife-103945-fig2-data2.zip › PINK1-2.png]

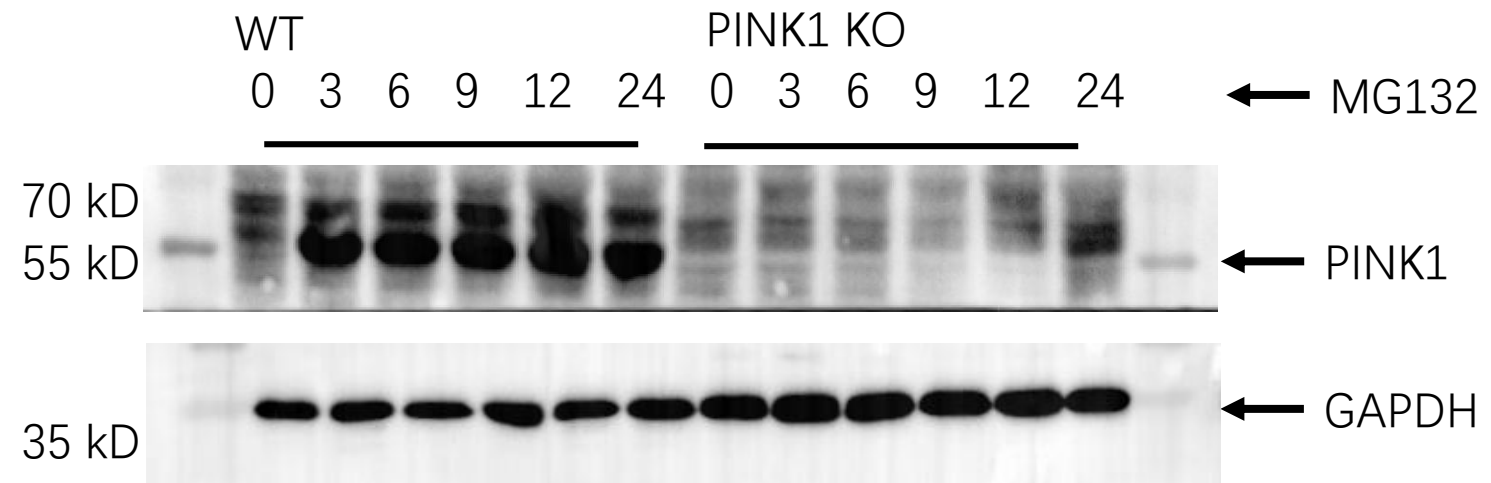

Supplement: Figure 2—source data 3. [file elife-103945-fig2-data3.pdf]

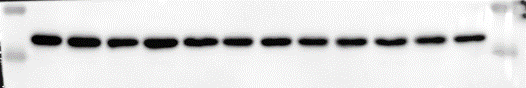

Supplement: Figure 2—source data 4. [file elife-103945-fig2-data4.zip › GAPDH 2C-1.png]

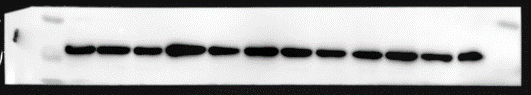

Supplement: Figure 2—source data 4. [file elife-103945-fig2-data4.zip › GAPDH 2C-2.png]

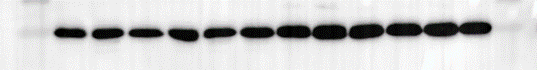

Supplement: Figure 2—source data 4. [file elife-103945-fig2-data4.zip › GAPDH 2C-3.png]

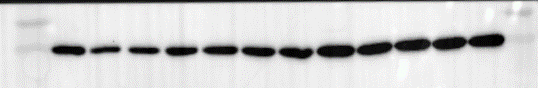

Supplement: Figure 2—source data 4. [file elife-103945-fig2-data4.zip › GAPDH 2C-4.png]

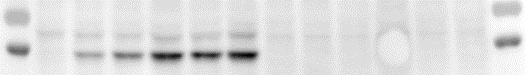

Supplement: Figure 2—source data 4. [file elife-103945-fig2-data4.zip › PINK·1 2C-1.png]

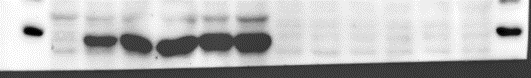

Supplement: Figure 2—source data 4. [file elife-103945-fig2-data4.zip › PINK·1 2C-2.png]

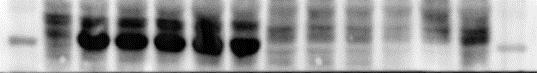

Supplement: Figure 2—source data 4. [file elife-103945-fig2-data4.zip › PINK·1 2C-3.png]

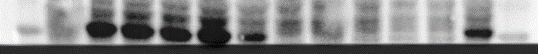

Supplement: Figure 2—source data 4. [file elife-103945-fig2-data4.zip › PINK·1 2C-4.png]

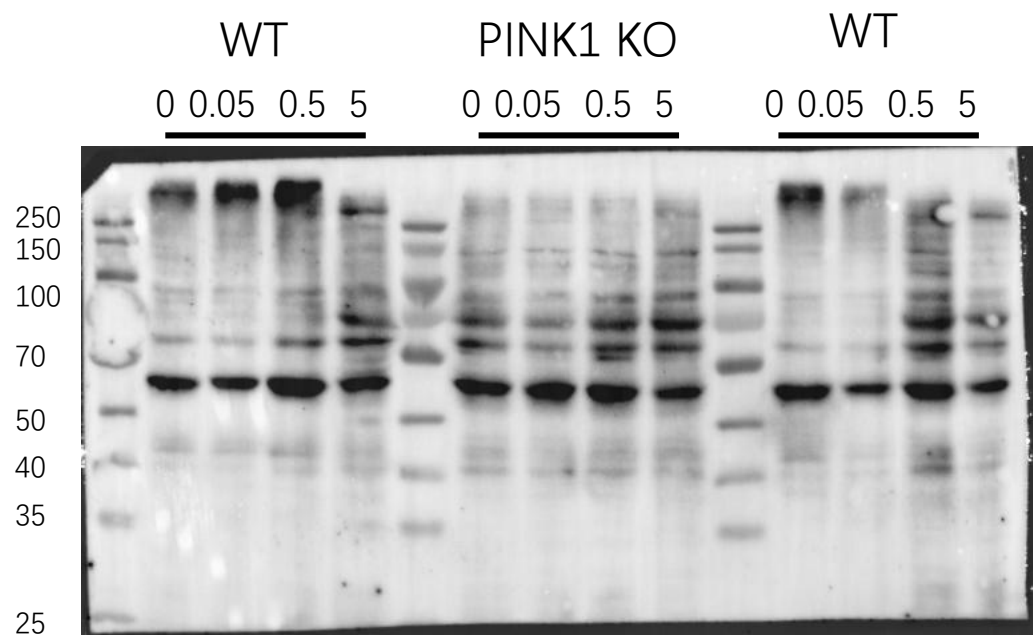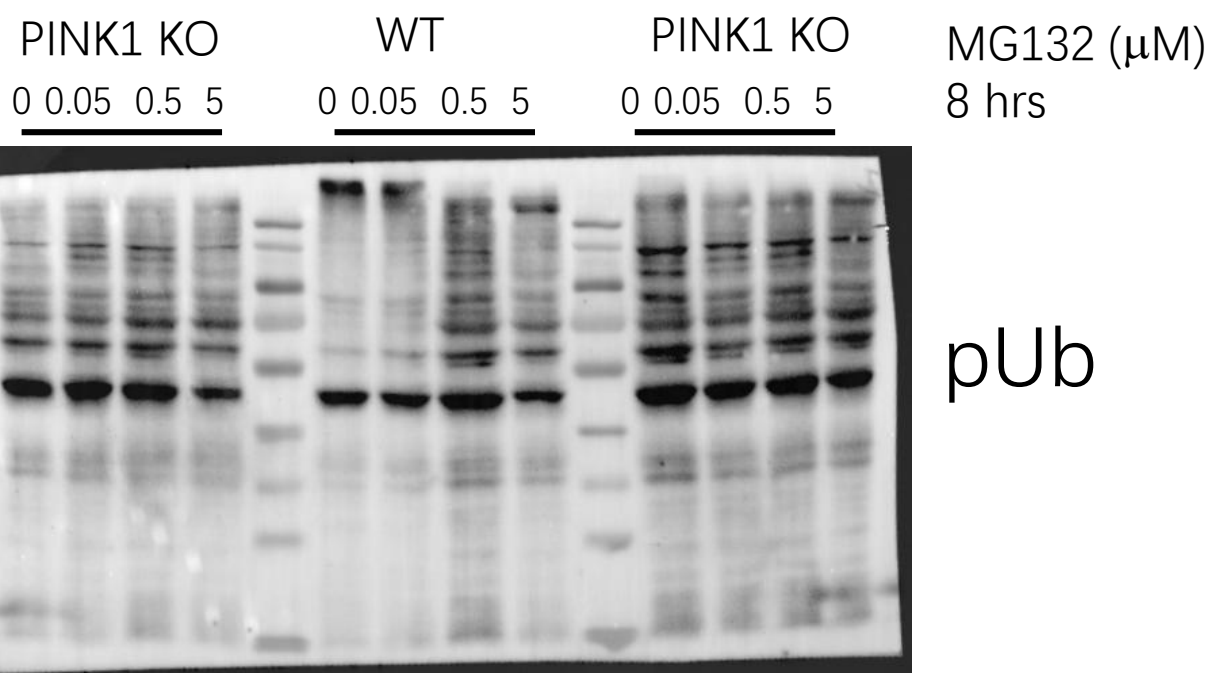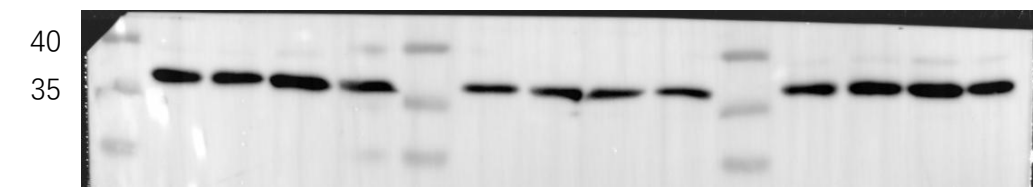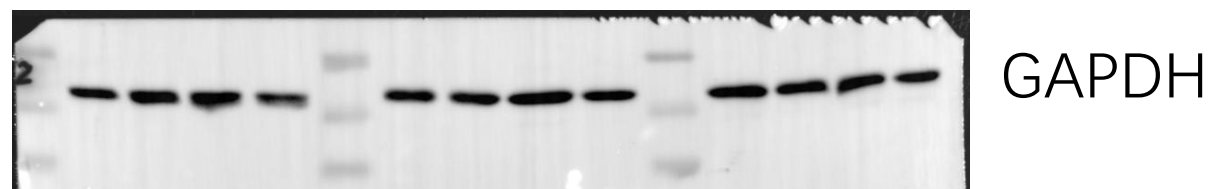

Supplement: Figure 2—source data 5. [file elife-103945-fig2-data5.pdf]

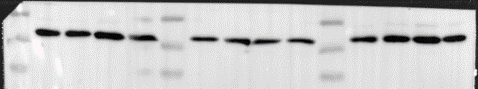

Supplement: Figure 2—source data 6. [file elife-103945-fig2-data6.zip › GAPDH-1.png]

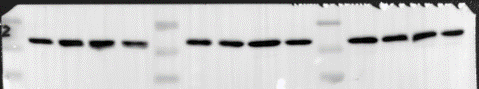

Supplement: Figure 2—source data 6. [file elife-103945-fig2-data6.zip › GAPDH-2.png]

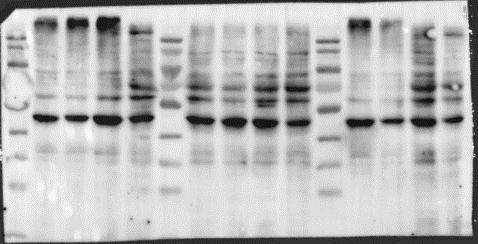

Supplement: Figure 2—source data 6. [file elife-103945-fig2-data6.zip › pUb-1.png]

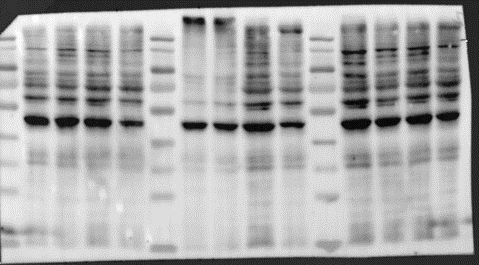

Supplement: Figure 2—source data 6. [file elife-103945-fig2-data6.zip › pUb-2.png]

MG132 (5  $\mu$ M)

|       | WT |   |   |   |    |    | PINK1 KO |   |   |   |    |    |
|-------|----|---|---|---|----|----|----------|---|---|---|----|----|
| Hours | 0  | 3 | 6 | 9 | 12 | 24 | 0        | 3 | 6 | 9 | 12 | 24 |

pUb

250  
150  
100  
70  
50  
40  
35

Ponceau staining

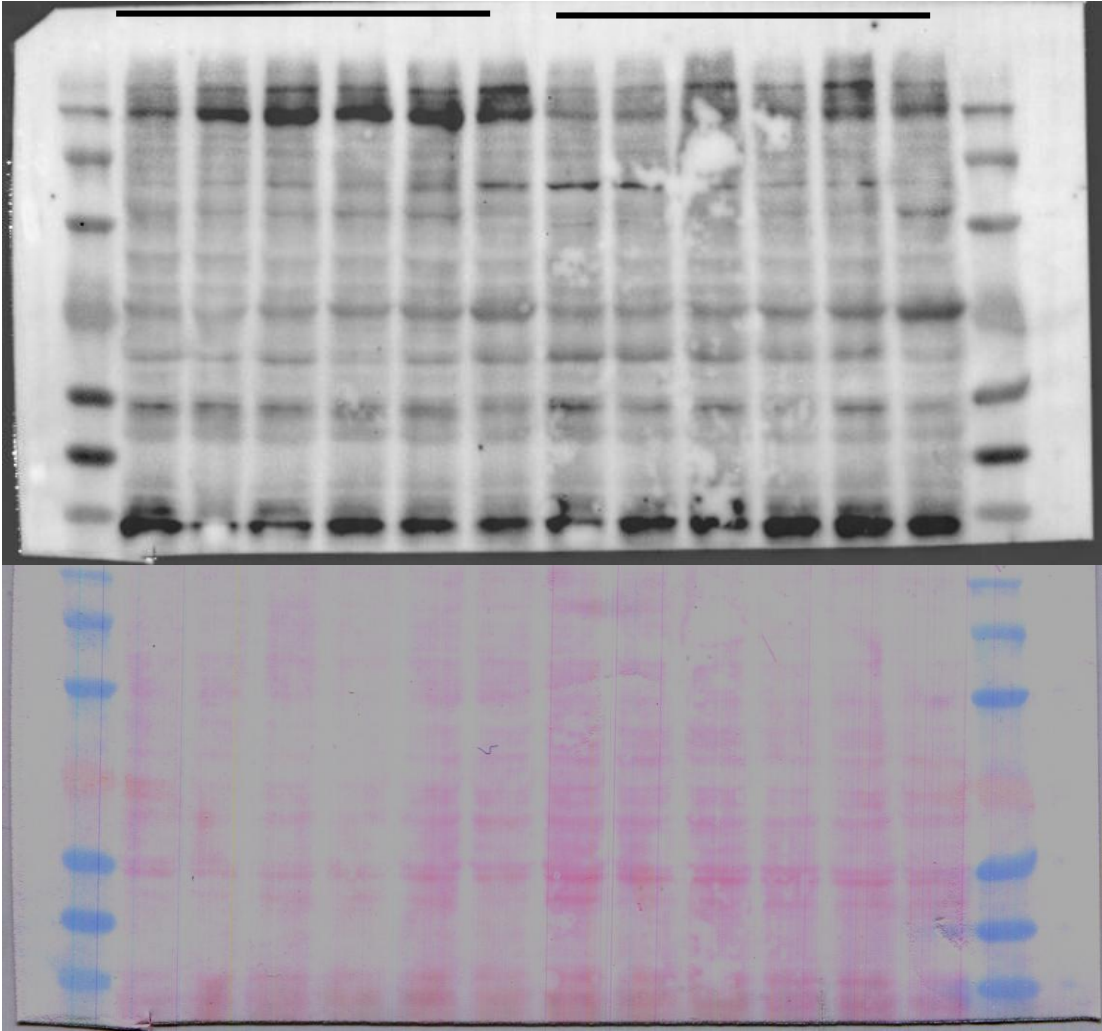

MG132 (5  $\mu$ M)

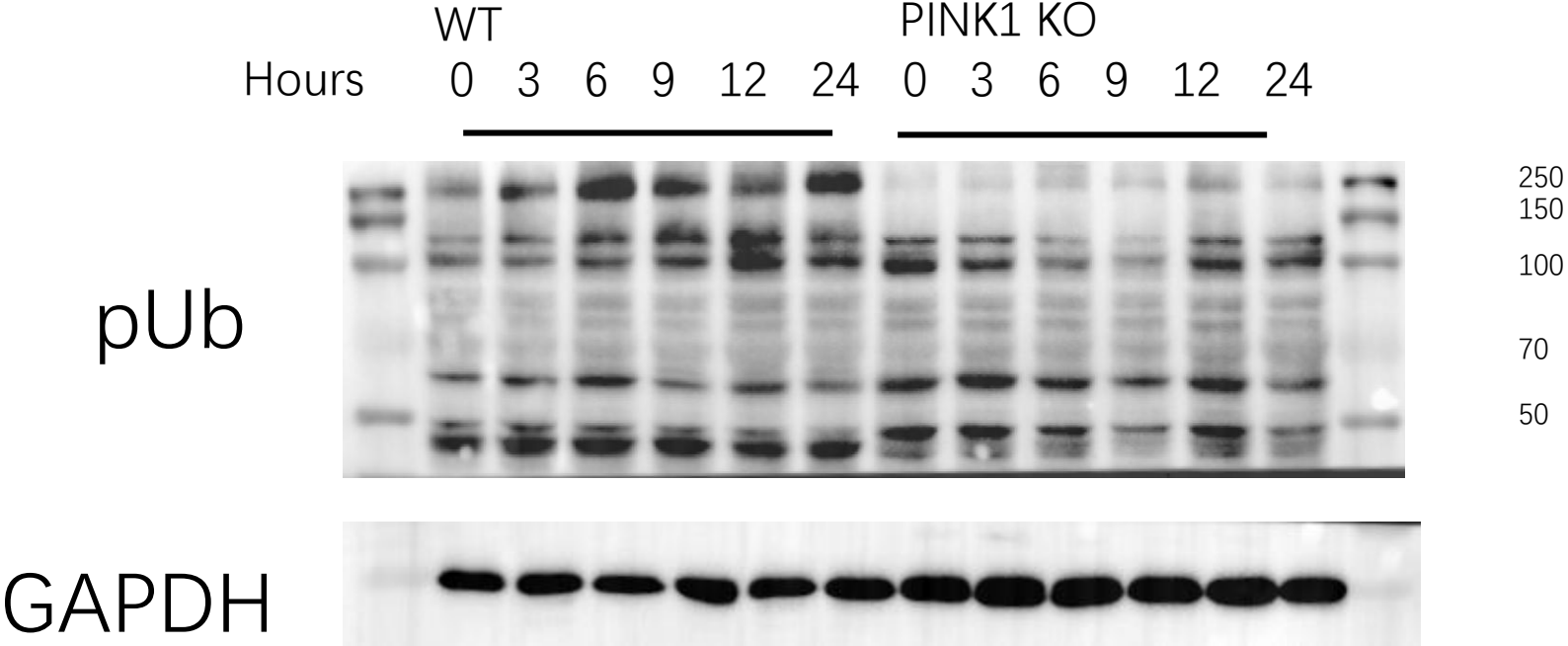

MG132 (5  $\mu$ M)

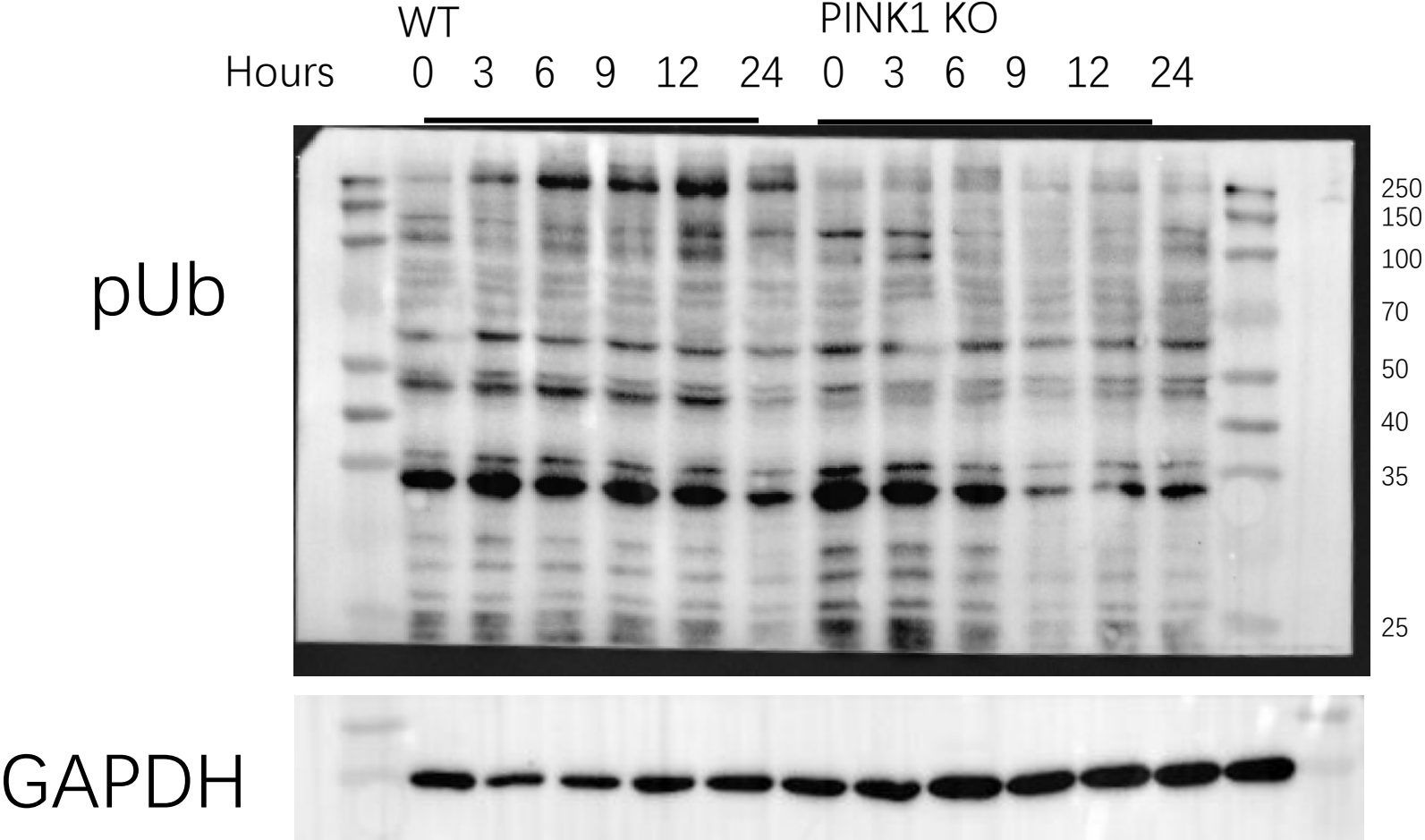

Supplement: Figure 2—source data 7. [file elife-103945-fig2-data7.pdf]

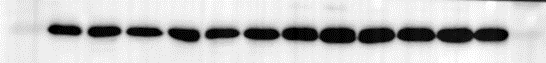

Supplement: Figure 2—source data 8. [file elife-103945-fig2-data8.zip › GAPDH-2.png]

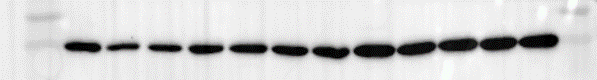

Supplement: Figure 2—source data 8. [file elife-103945-fig2-data8.zip › GAPDH-3.png]

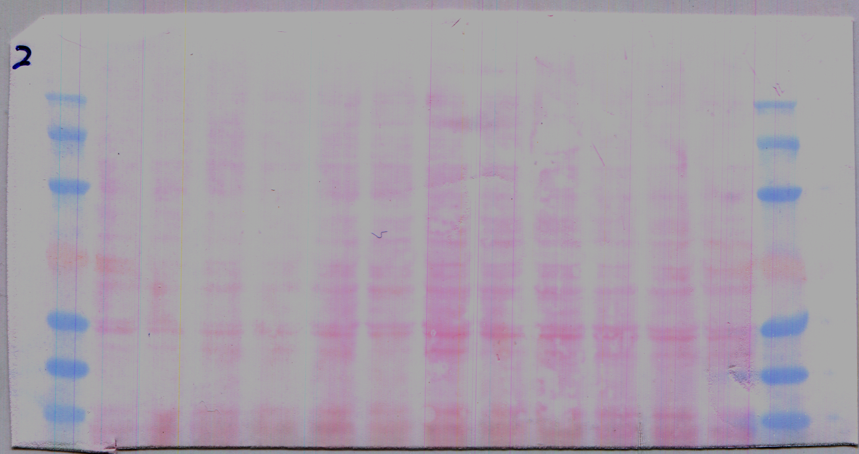

Supplement: Figure 2—source data 8. [file elife-103945-fig2-data8.zip › Ponceau staining.tif]

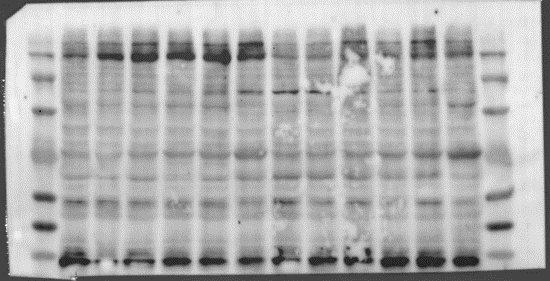

Supplement: Figure 2—source data 8. [file elife-103945-fig2-data8.zip › pUb-1.png]

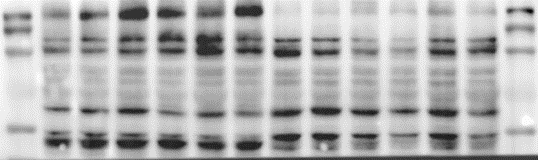

Supplement: Figure 2—source data 8. [file elife-103945-fig2-data8.zip › pUb-2.png]

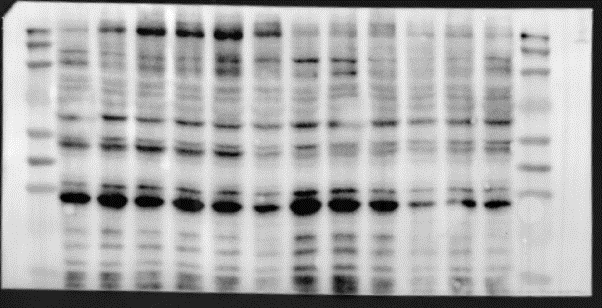

Supplement: Figure 2—source data 8. [file elife-103945-fig2-data8.zip › pUb-3.png]

Ub-R-EGFP

blank MG132 sPINK1 sPINK1KD

35kD

25kD

GFP

35kD

GAPDH

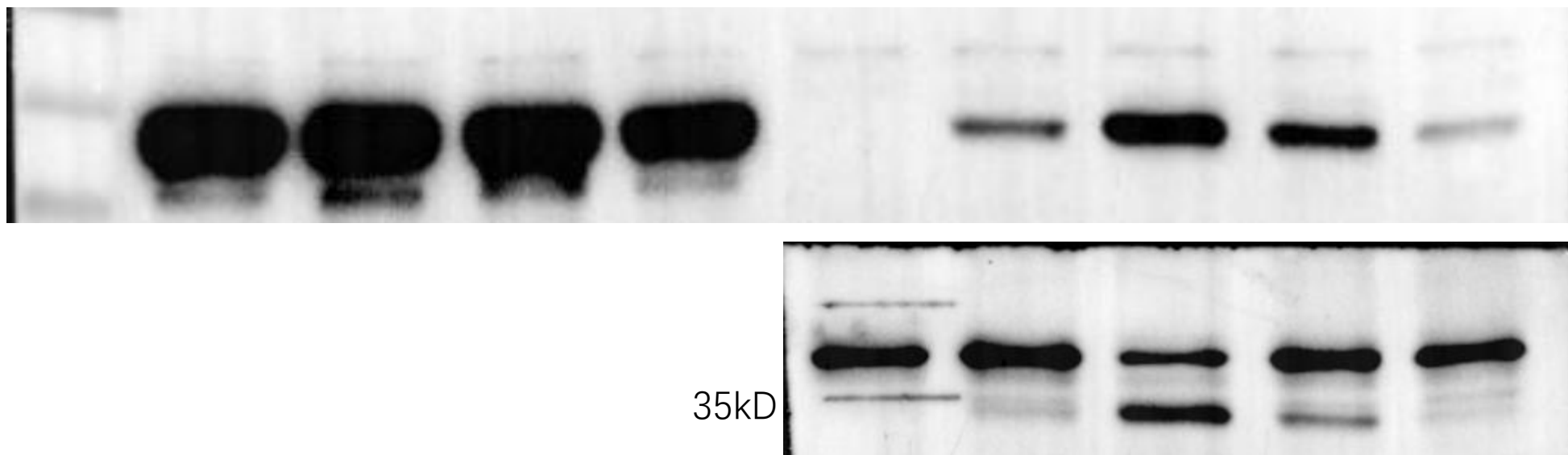

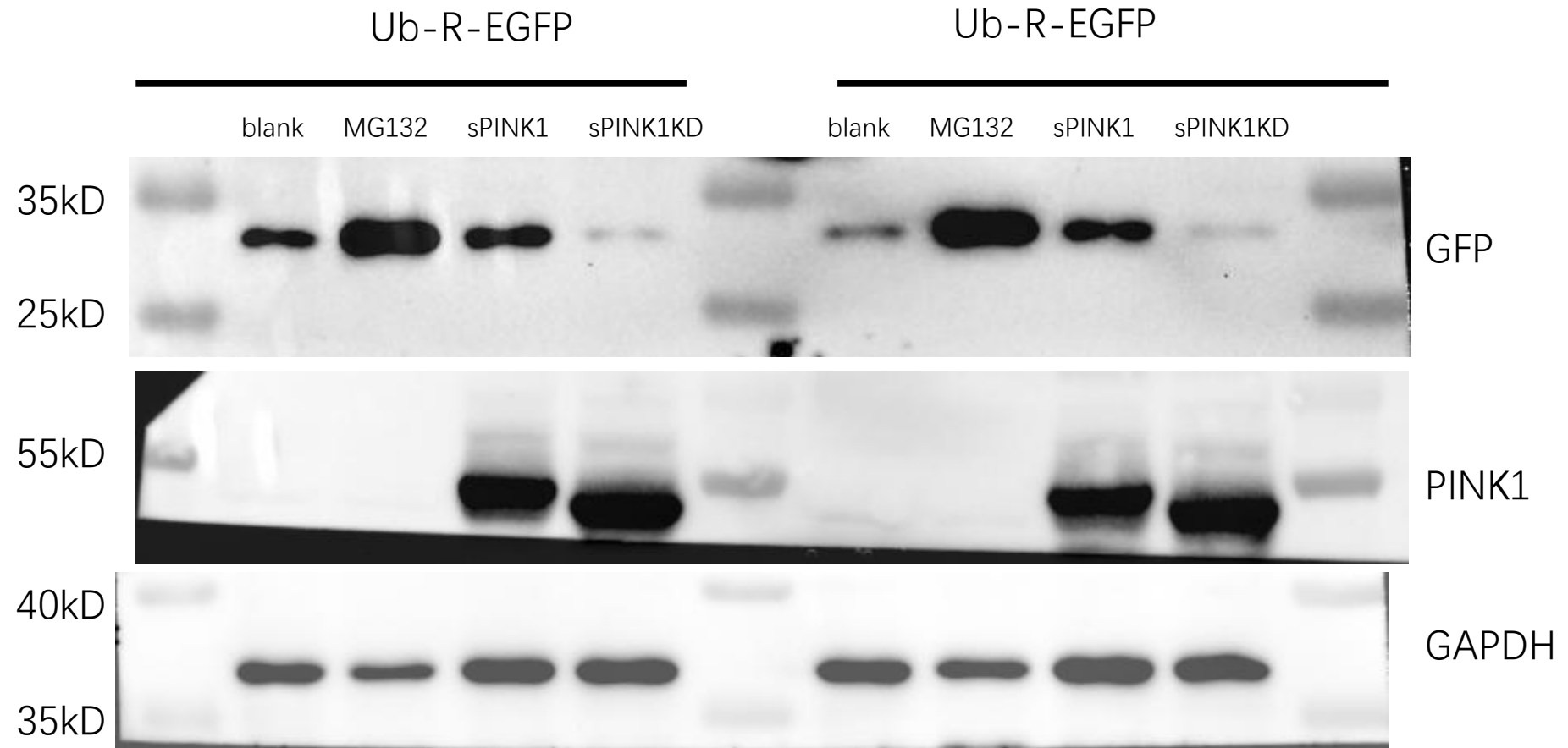

Supplement: Figure 2—source data 9. [file elife-103945-fig2-data9.pdf]

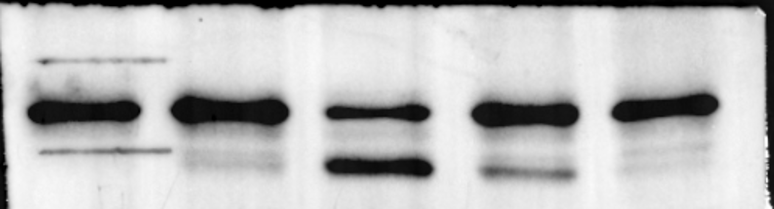

Supplement: Figure 2—source data 10. [file elife-103945-fig2-data10.zip › GAPDH-1.tif]

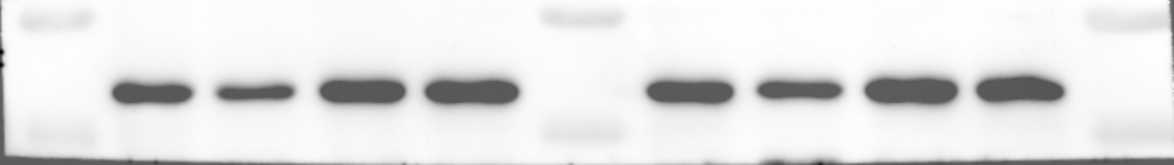

Supplement: Figure 2—source data 10. [file elife-103945-fig2-data10.zip › GAPDH-2.tif]

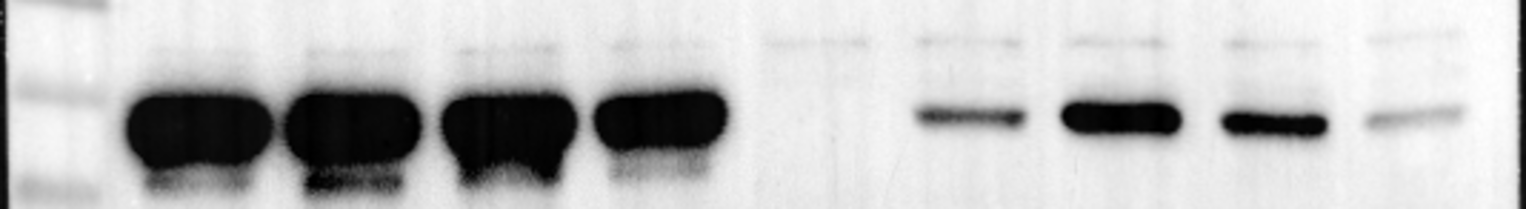

Supplement: Figure 2—source data 10. [file elife-103945-fig2-data10.zip › GFP-1.tif]

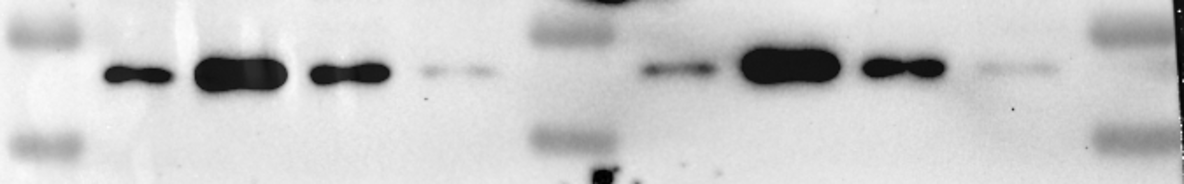

Supplement: Figure 2—source data 10. [file elife-103945-fig2-data10.zip › GFP-2.tif]

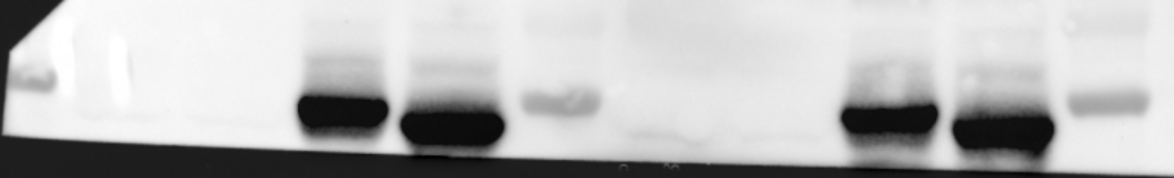

Supplement: Figure 2—source data 10. [file elife-103945-fig2-data10.zip › PINK1.tif]

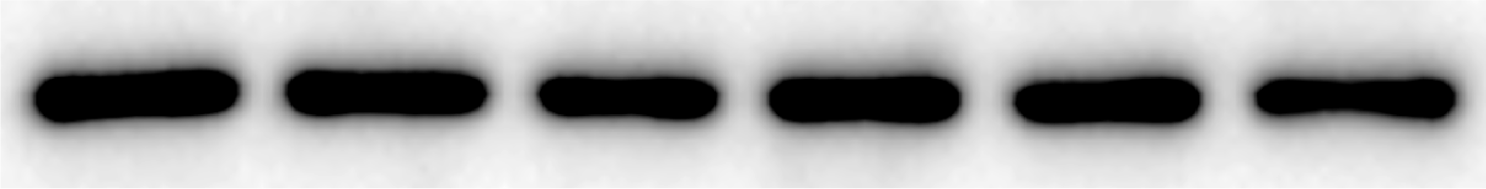

Supplement: Figure 3—source data 2. [file elife-103945-fig3-data2.zip › 1-1.tif]

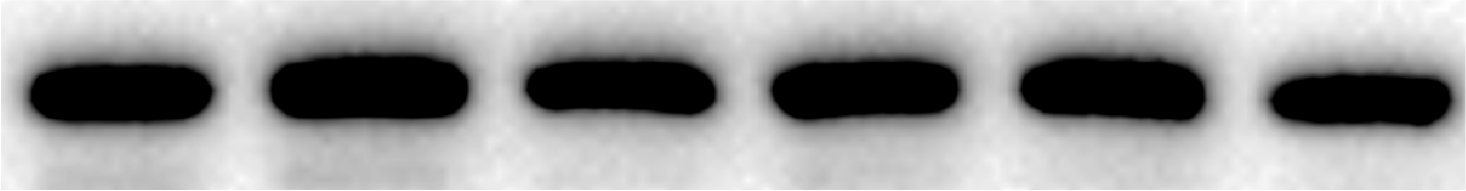

Supplement: Figure 3—source data 2. [file elife-103945-fig3-data2.zip › 1-2.tif]

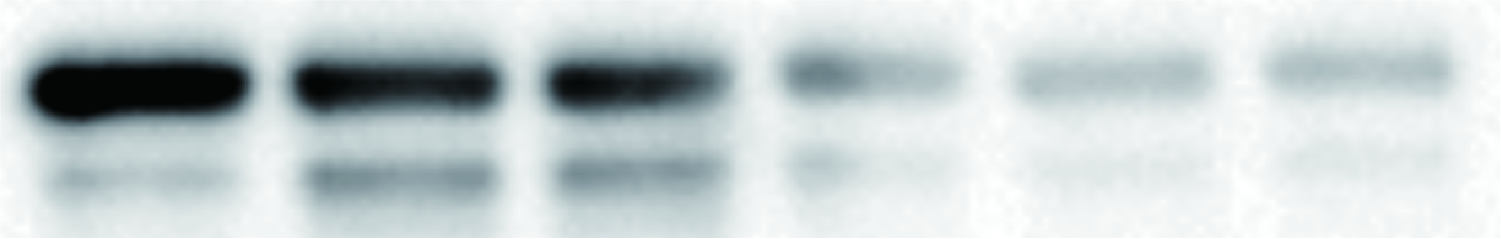

Supplement: Figure 3—source data 2. [file elife-103945-fig3-data2.zip › 1-3.tif]

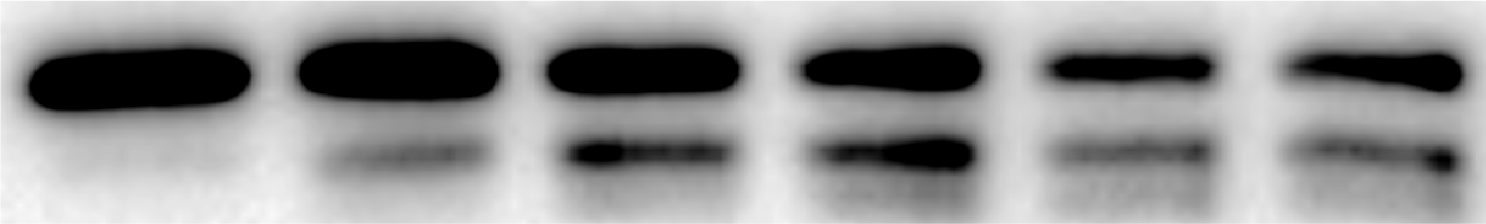

Supplement: Figure 3—source data 2. [file elife-103945-fig3-data2.zip › 1-4.tif]

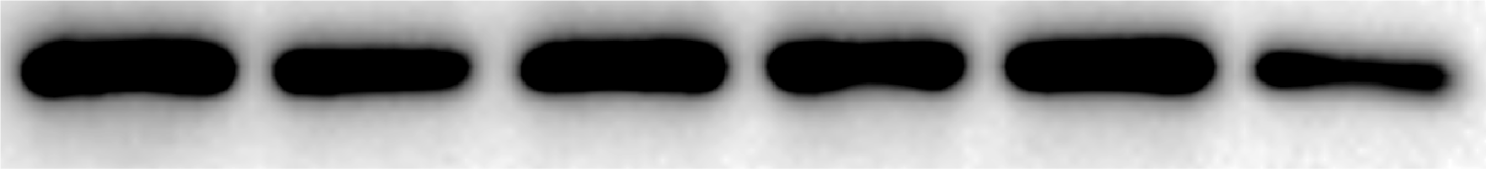

Supplement: Figure 3—source data 2. [file elife-103945-fig3-data2.zip › 2-1.tif]

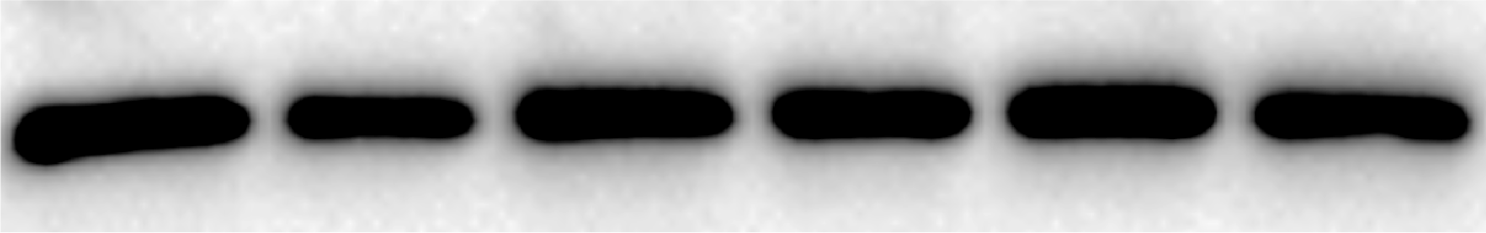

Supplement: Figure 3—source data 2. [file elife-103945-fig3-data2.zip › 2-2.tif]

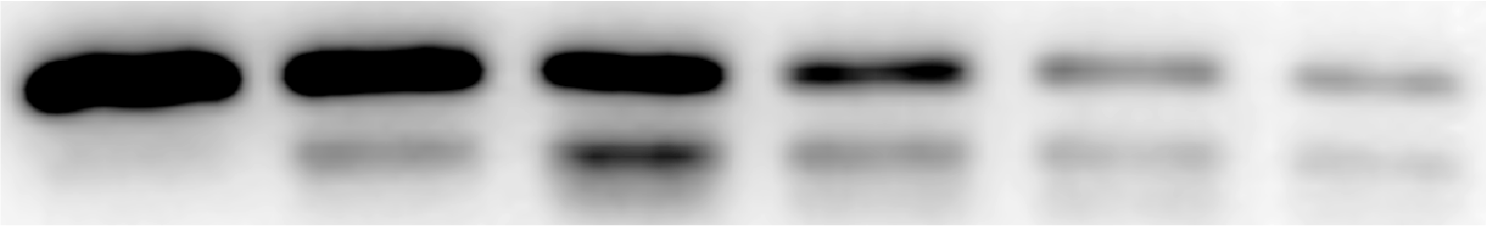

Supplement: Figure 3—source data 2. [file elife-103945-fig3-data2.zip › 2-3.tif]

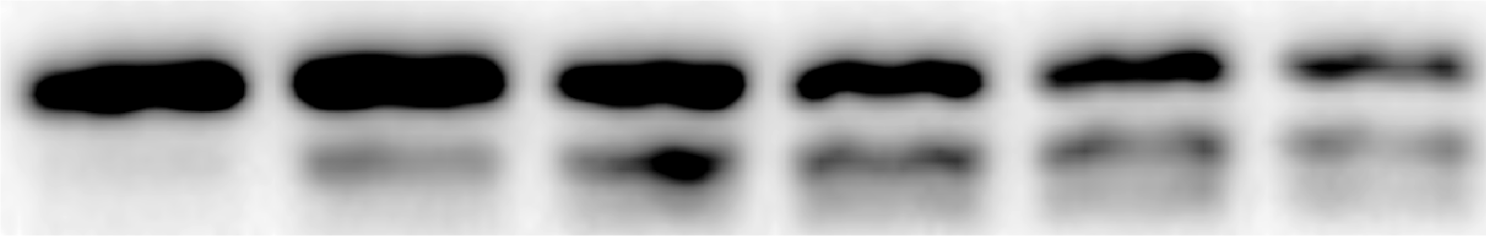

Supplement: Figure 3—source data 2. [file elife-103945-fig3-data2.zip › 2-4.tif]

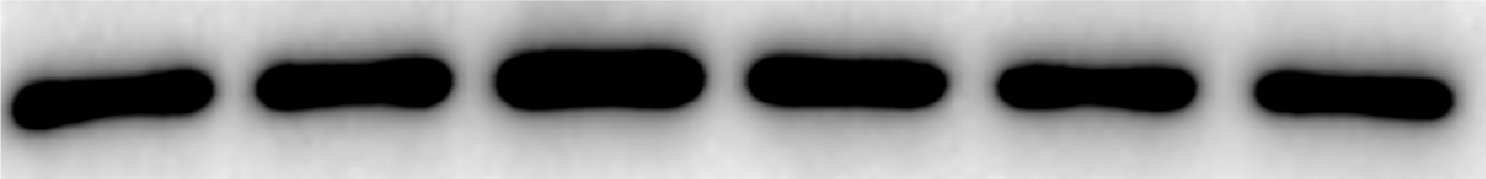

Supplement: Figure 3—source data 2. [file elife-103945-fig3-data2.zip › 3-1.tif]

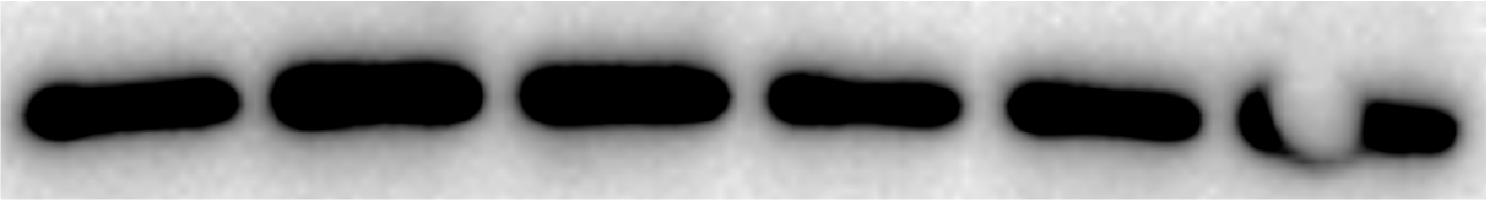

Supplement: Figure 3—source data 2. [file elife-103945-fig3-data2.zip › 3-2.tif]

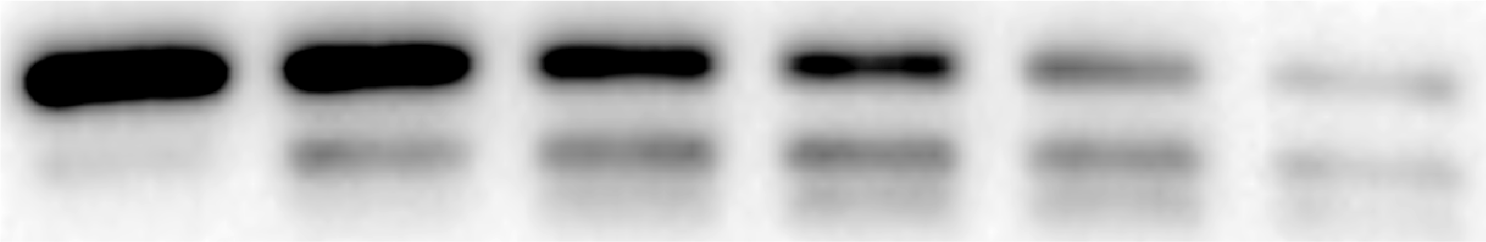

Supplement: Figure 3—source data 2. [file elife-103945-fig3-data2.zip › 3-3.tif]

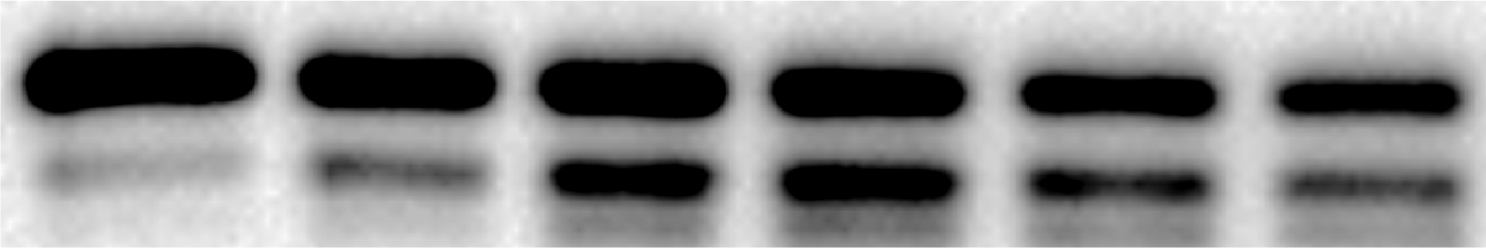

Supplement: Figure 3—source data 2. [file elife-103945-fig3-data2.zip › 3-4.tif]

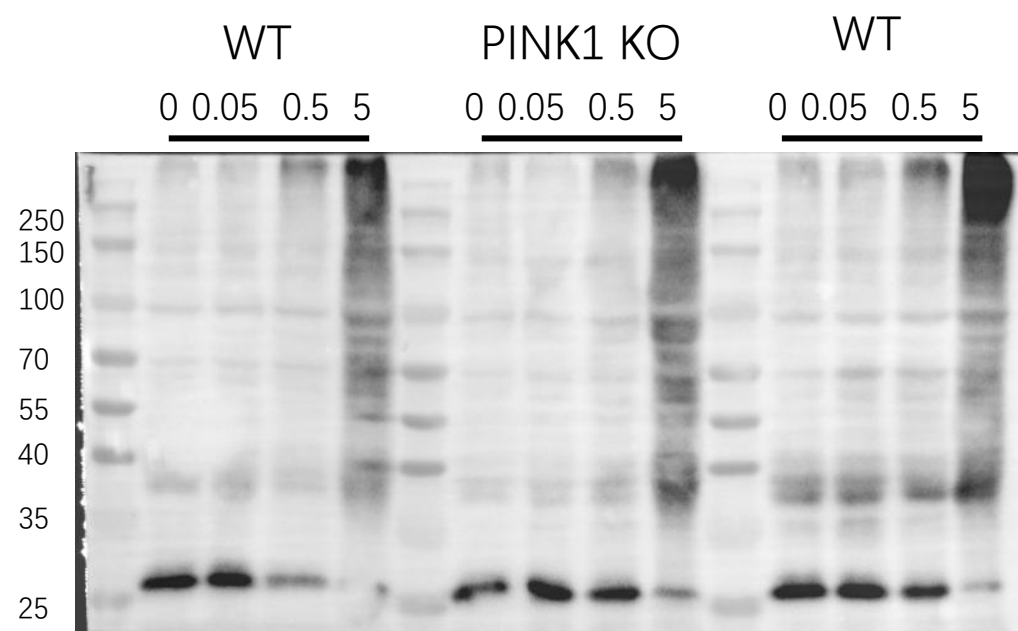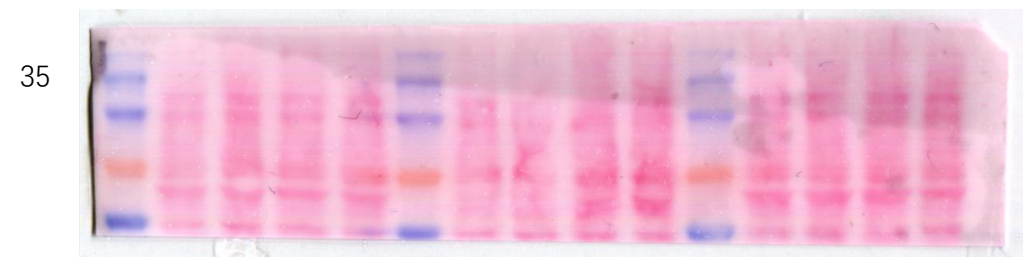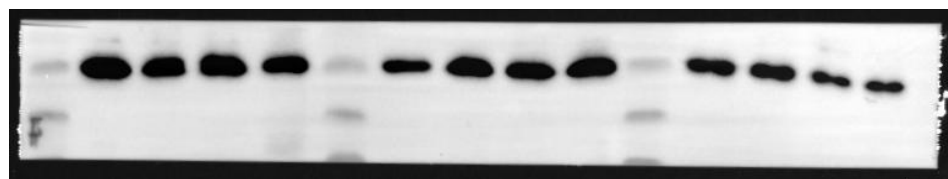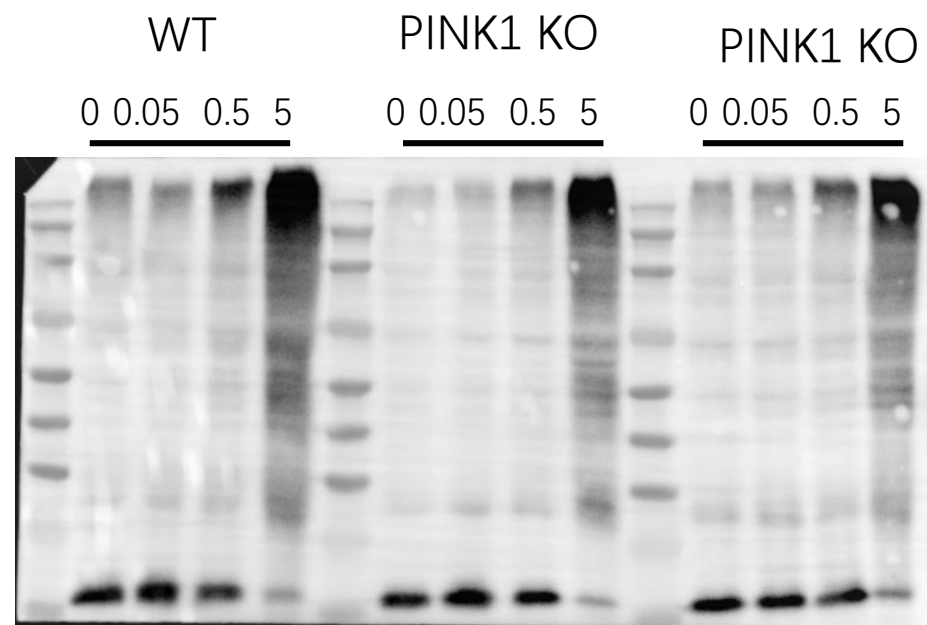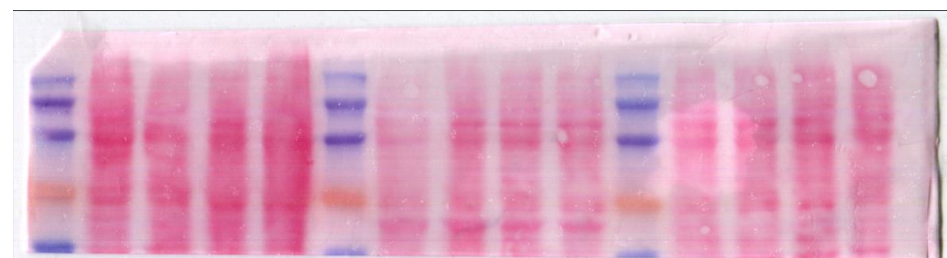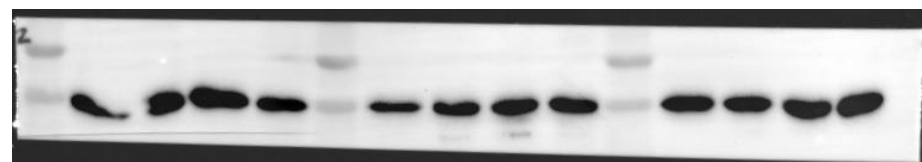

Ub

Ponceau staining

GAPDH

Supplement: Figure 4—source data 1. [file elife-103945-fig4-data1.pdf]

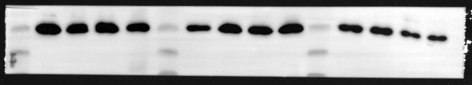

Supplement: Figure 4—source data 2. [file elife-103945-fig4-data2.zip › GAPDH-1.tif]

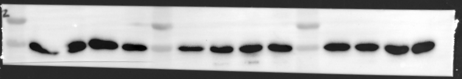

Supplement: Figure 4—source data 2. [file elife-103945-fig4-data2.zip › GAPDH-2.tif]

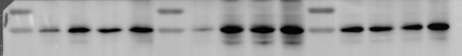

Supplement: Figure 4—source data 2. [file elife-103945-fig4-data2.zip › GAPDH-3.tif]

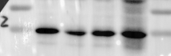

Supplement: Figure 4—source data 2. [file elife-103945-fig4-data2.zip › GAPDH-4.tif]

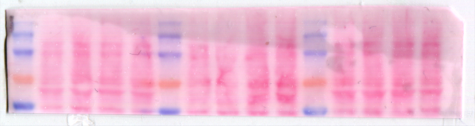

Supplement: Figure 4—source data 2. [file elife-103945-fig4-data2.zip › insoluble ponceau staining-1.tif]

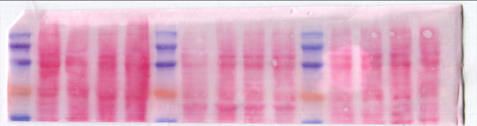

Supplement: Figure 4—source data 2. [file elife-103945-fig4-data2.zip › insoluble ponceau staining-2.tif]

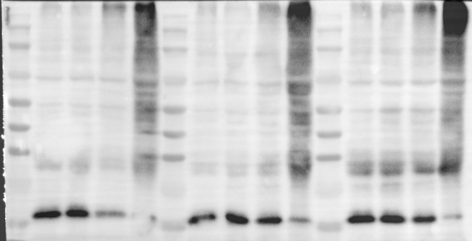

Supplement: Figure 4—source data 2. [file elife-103945-fig4-data2.zip › insoluble Ub-1.tif]

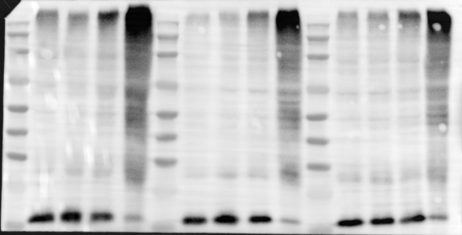

Supplement: Figure 4—source data 2. [file elife-103945-fig4-data2.zip › insoluble Ub-2.tif]

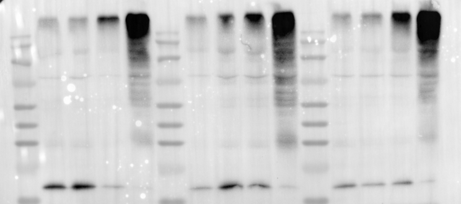

Supplement: Figure 4—source data 2. [file elife-103945-fig4-data2.zip › insoluble Ub-3.tif]

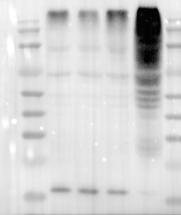

Supplement: Figure 4—source data 2. [file elife-103945-fig4-data2.zip › insoluble Ub-4.tif]

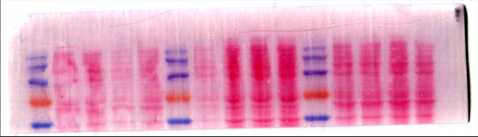

Supplement: Figure 4—source data 2. [file elife-103945-fig4-data2.zip › Ponceau stainging-3.tif]

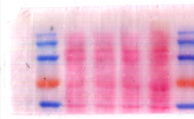

Supplement: Figure 4—source data 2. [file elife-103945-fig4-data2.zip › Ponceau stainging-4.tif]

0

3

6

9

12

4

4

0

3

6

9

12

4

4

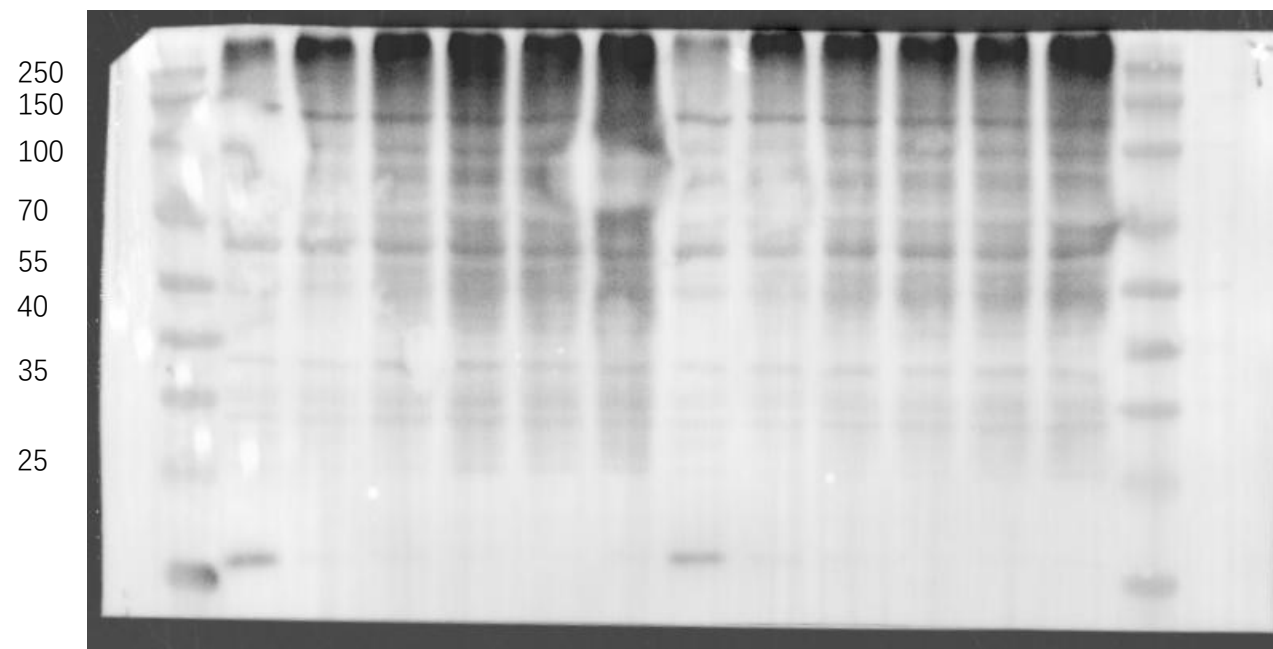

Ub

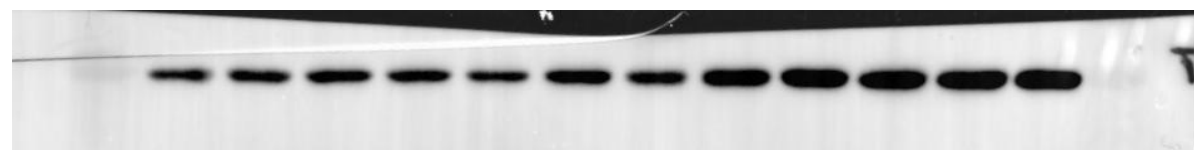

GAPDH

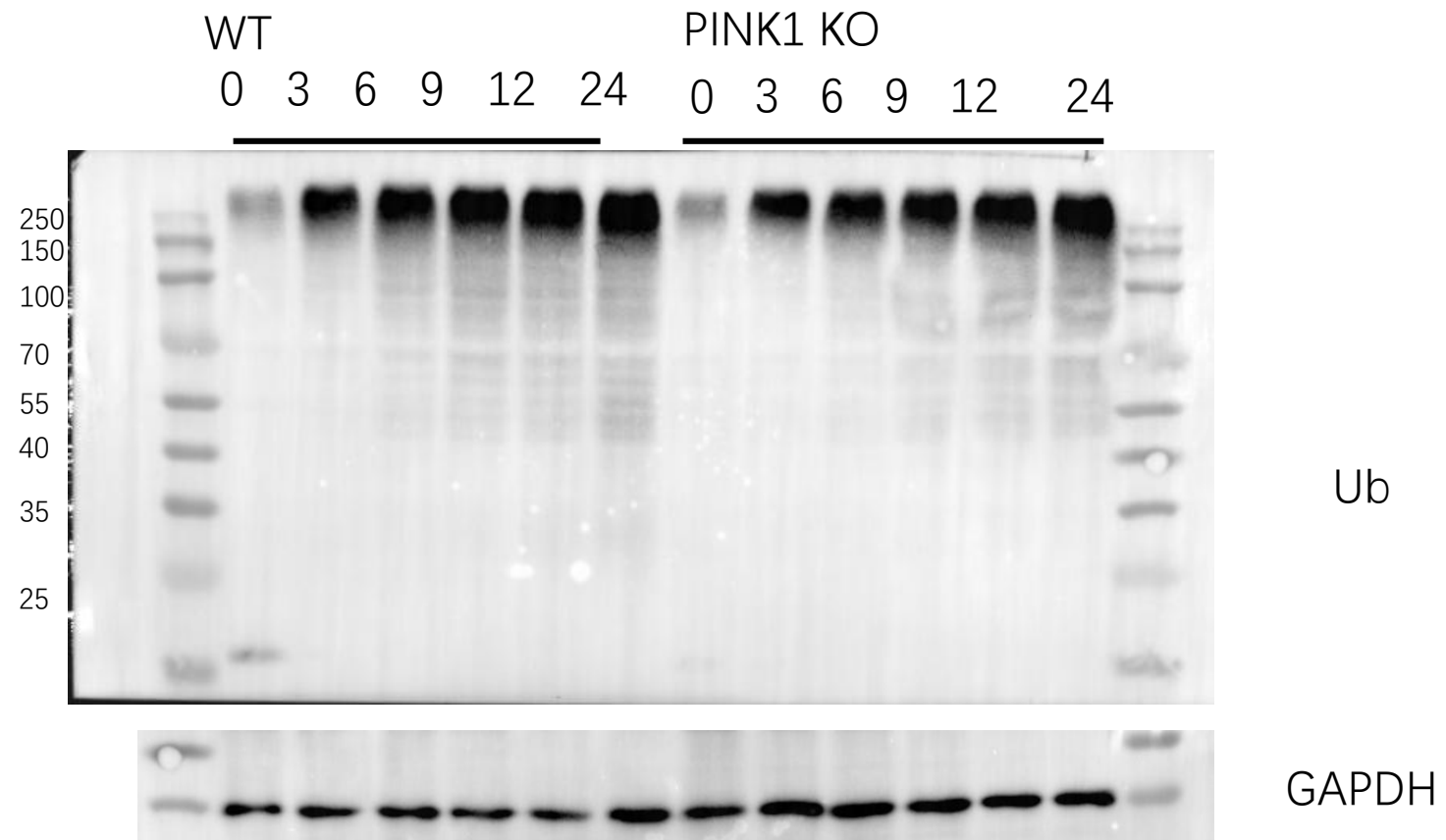

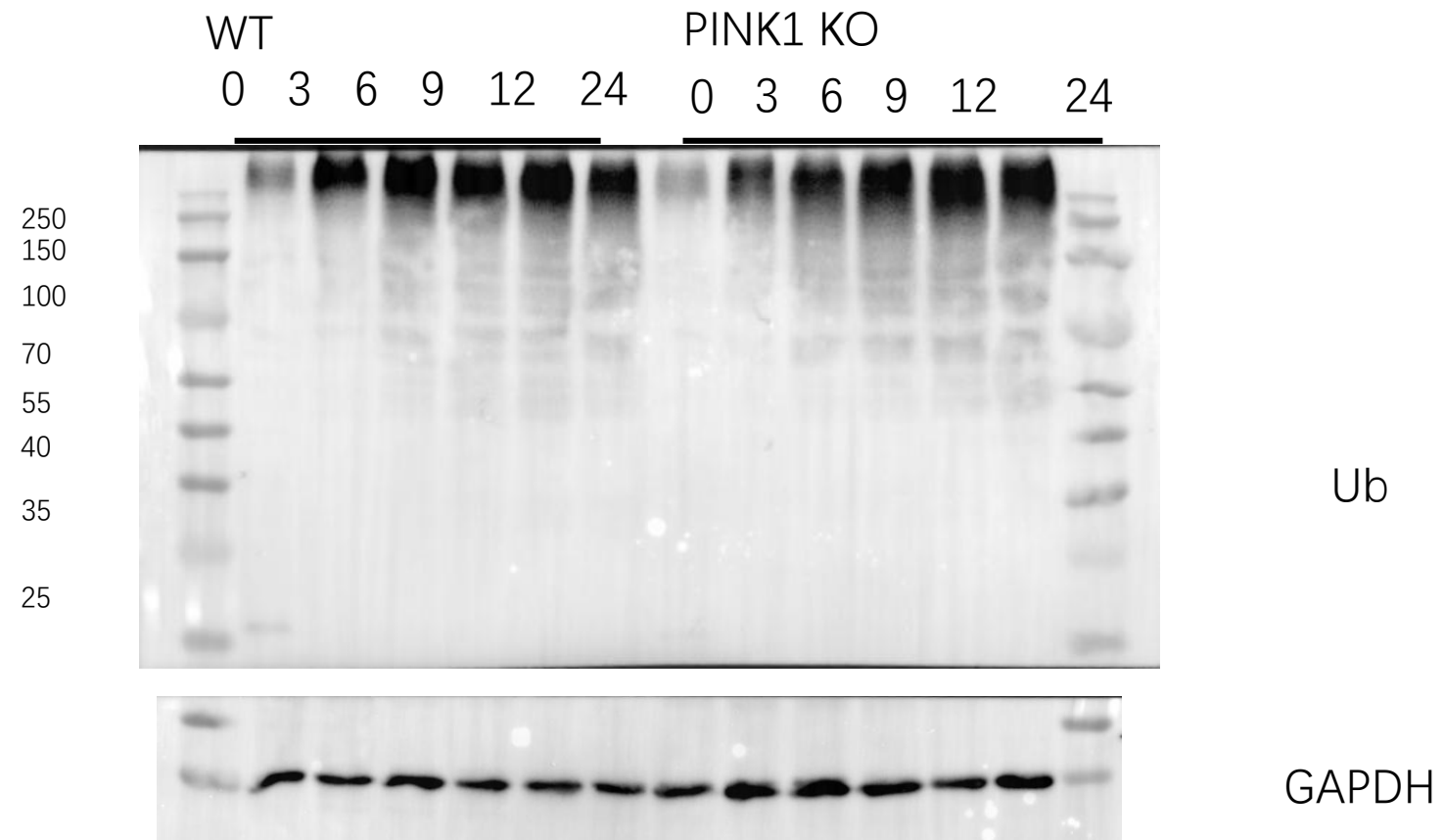

Supplement: Figure 4—source data 3. [file elife-103945-fig4-data3.pdf]

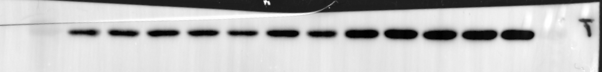

Supplement: Figure 4—source data 4. [file elife-103945-fig4-data4.zip › GAPDH-1.tif]

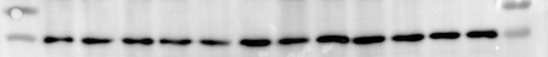

Supplement: Figure 4—source data 4. [file elife-103945-fig4-data4.zip › GAPDH-2.tif]

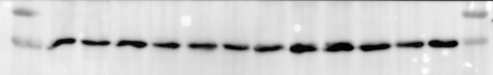

Supplement: Figure 4—source data 4. [file elife-103945-fig4-data4.zip › GAPDH-3.tif]

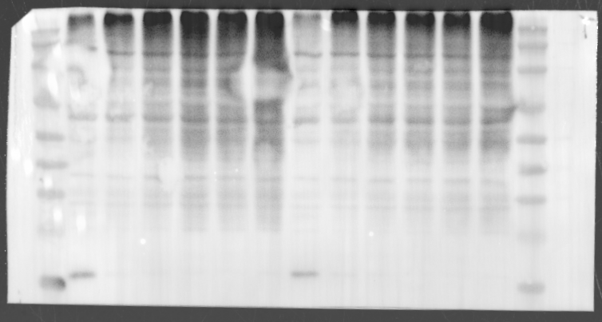

Supplement: Figure 4—source data 4. [file elife-103945-fig4-data4.zip › insoluble Ub-1.tif]

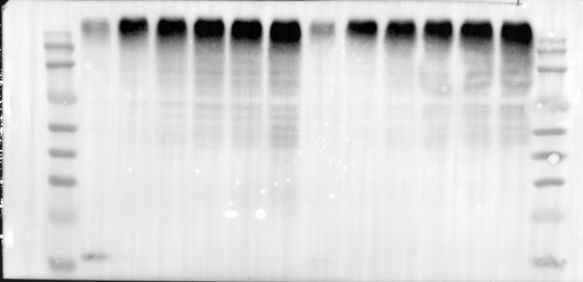

Supplement: Figure 4—source data 4. [file elife-103945-fig4-data4.zip › insoluble Ub-2.tif]

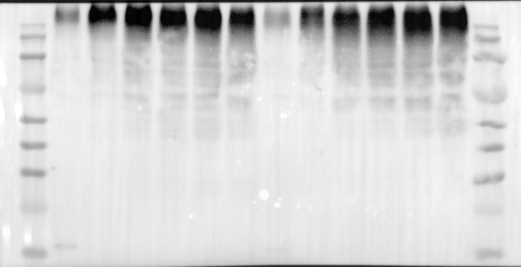

Supplement: Figure 4—source data 4. [file elife-103945-fig4-data4.zip › insoluble Ub-3.tif]

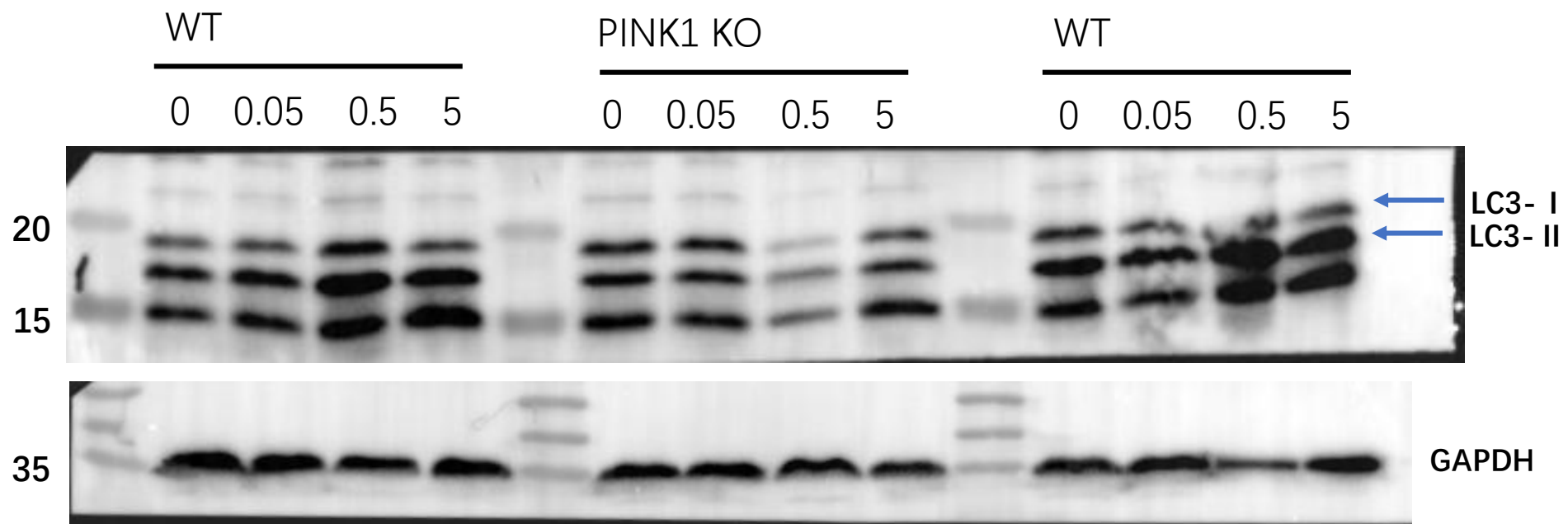

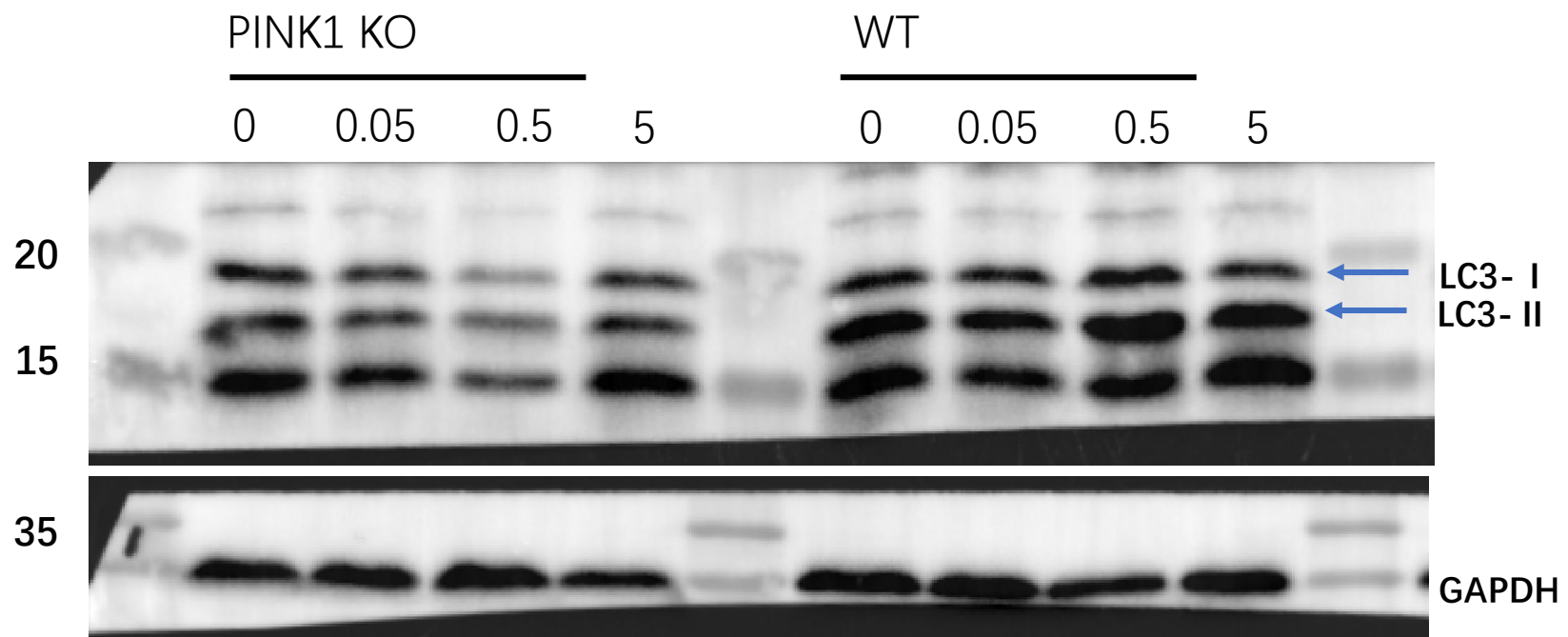

Supplement: Figure 4—source data 5. [file elife-103945-fig4-data5.pdf]

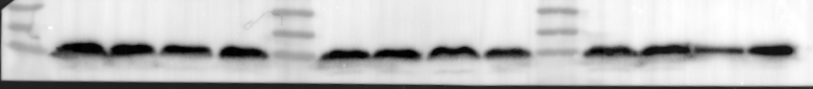

Supplement: Figure 4—source data 6. [file elife-103945-fig4-data6.zip › GAPDH-1.tif]

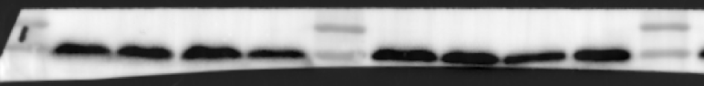

Supplement: Figure 4—source data 6. [file elife-103945-fig4-data6.zip › GAPDH-2.tif]

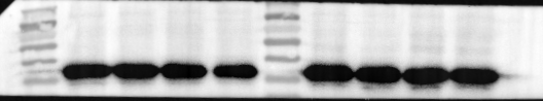

Supplement: Figure 4—source data 6. [file elife-103945-fig4-data6.zip › GAPDH-3.tif]

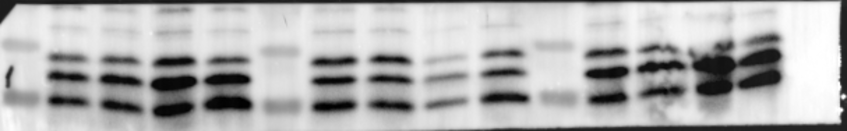

Supplement: Figure 4—source data 6. [file elife-103945-fig4-data6.zip › LC3-1.tif]

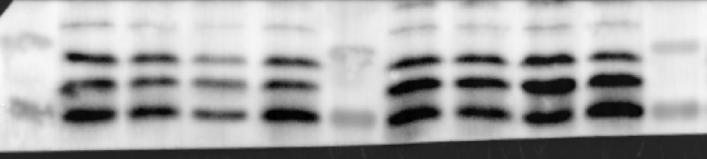

Supplement: Figure 4—source data 6. [file elife-103945-fig4-data6.zip › LC3-2.tif]

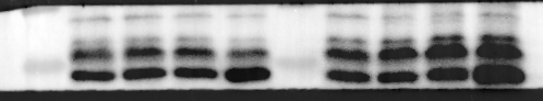

Supplement: Figure 4—source data 6. [file elife-103945-fig4-data6.zip › LC3-3.tif]

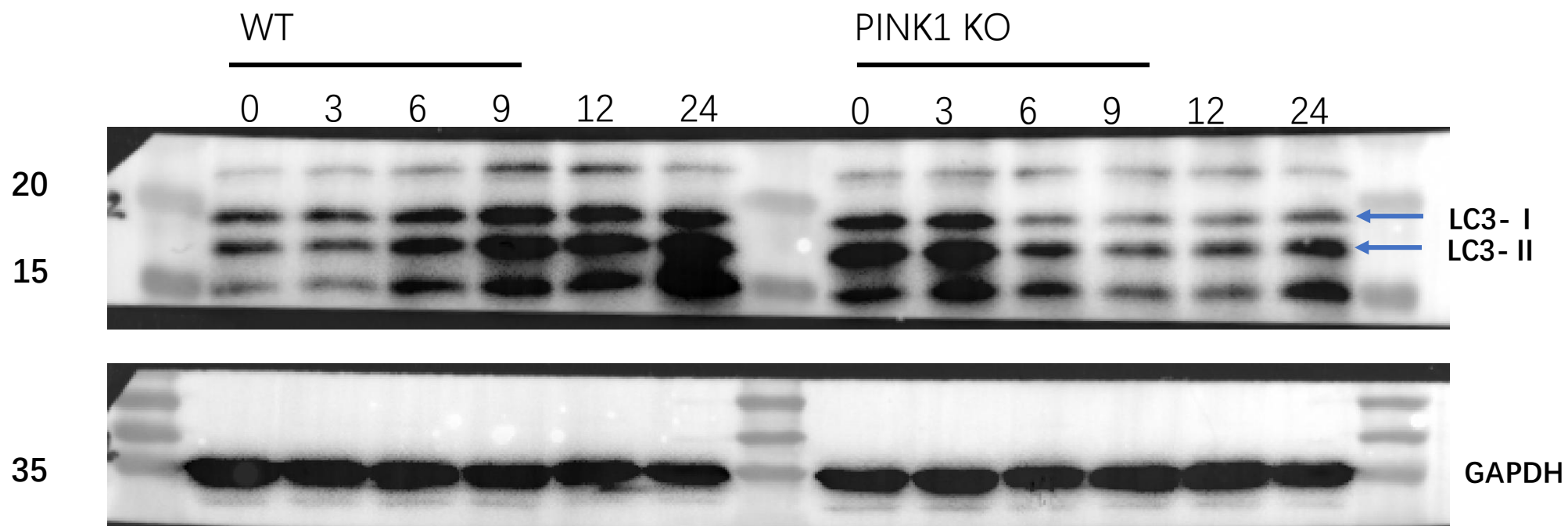

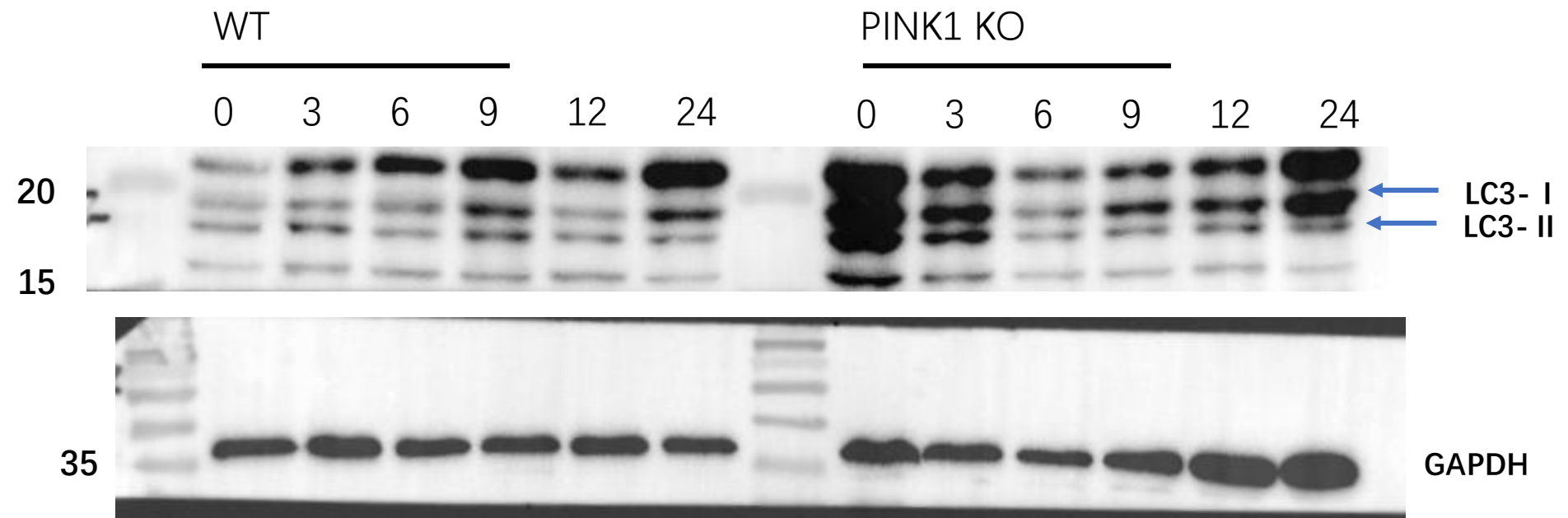

Supplement: Figure 4—source data 7. [file elife-103945-fig4-data7.pdf]

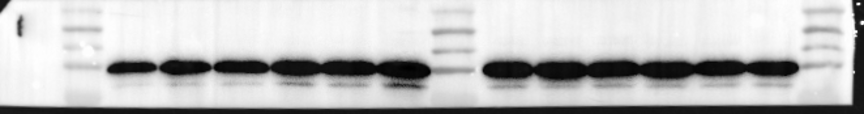

Supplement: Figure 4—source data 8. [file elife-103945-fig4-data8.zip › GAPDH-1.tif]

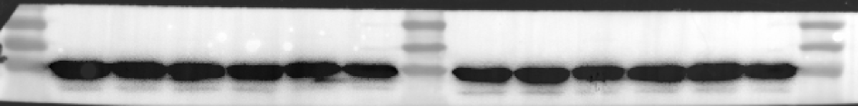

Supplement: Figure 4—source data 8. [file elife-103945-fig4-data8.zip › GAPDH-2.tif]

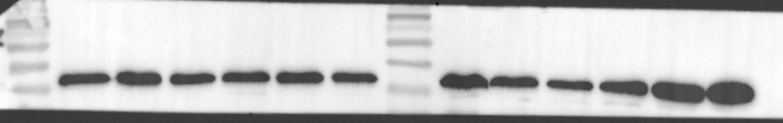

Supplement: Figure 4—source data 8. [file elife-103945-fig4-data8.zip › GAPDH-3.tif]

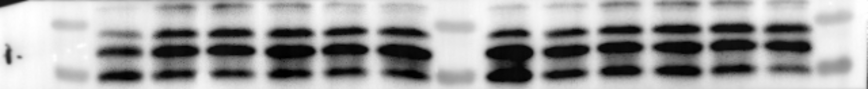

Supplement: Figure 4—source data 8. [file elife-103945-fig4-data8.zip › LC3-1.tif]

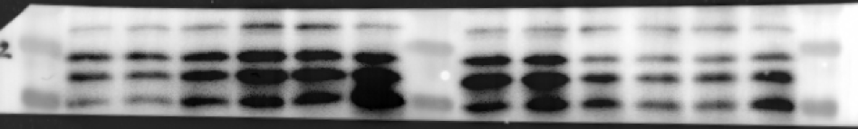

Supplement: Figure 4—source data 8. [file elife-103945-fig4-data8.zip › LC3-2.tif]

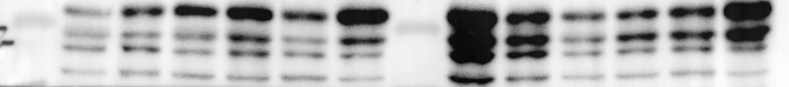

Supplement: Figure 4—source data 8. [file elife-103945-fig4-data8.zip › LC3-3.tif]

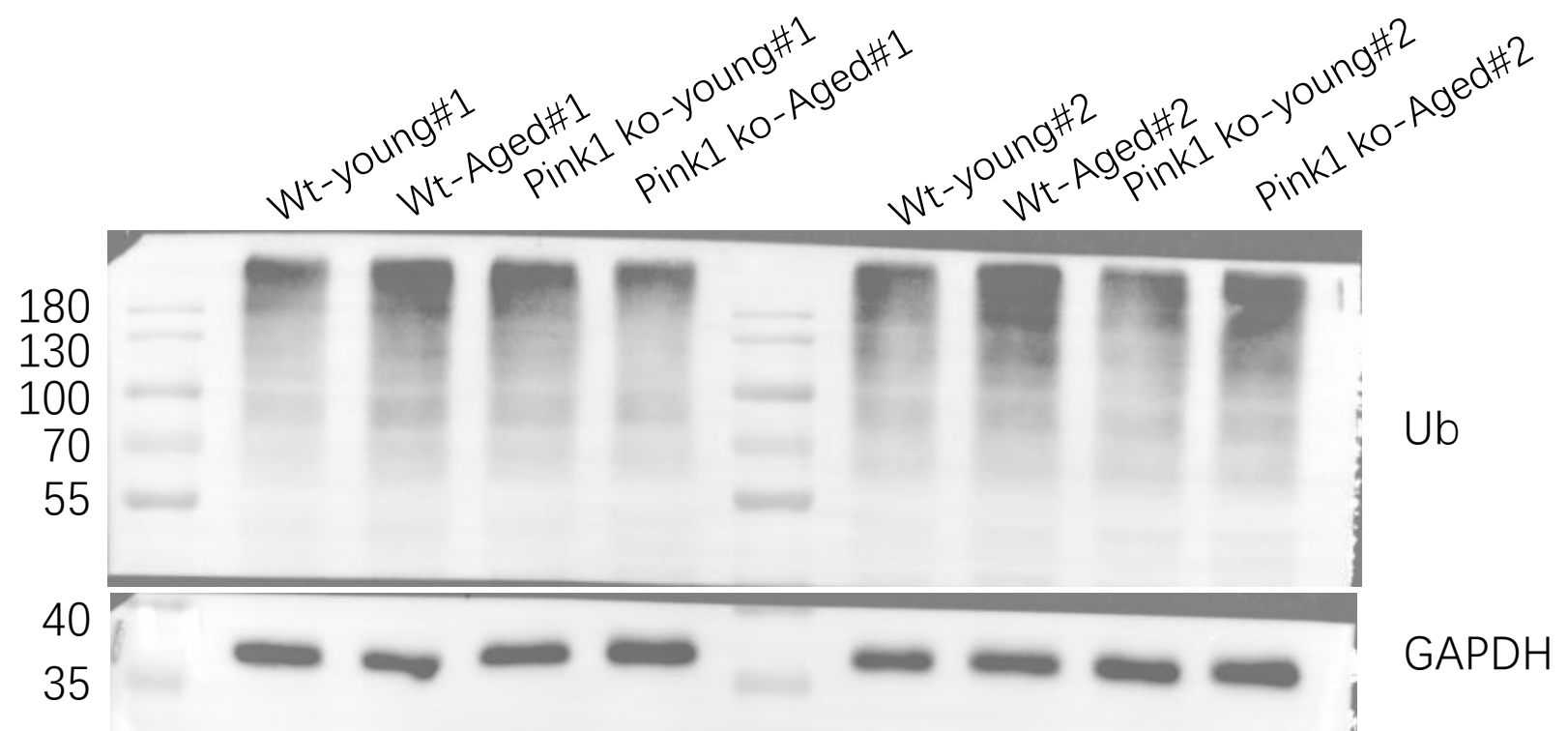

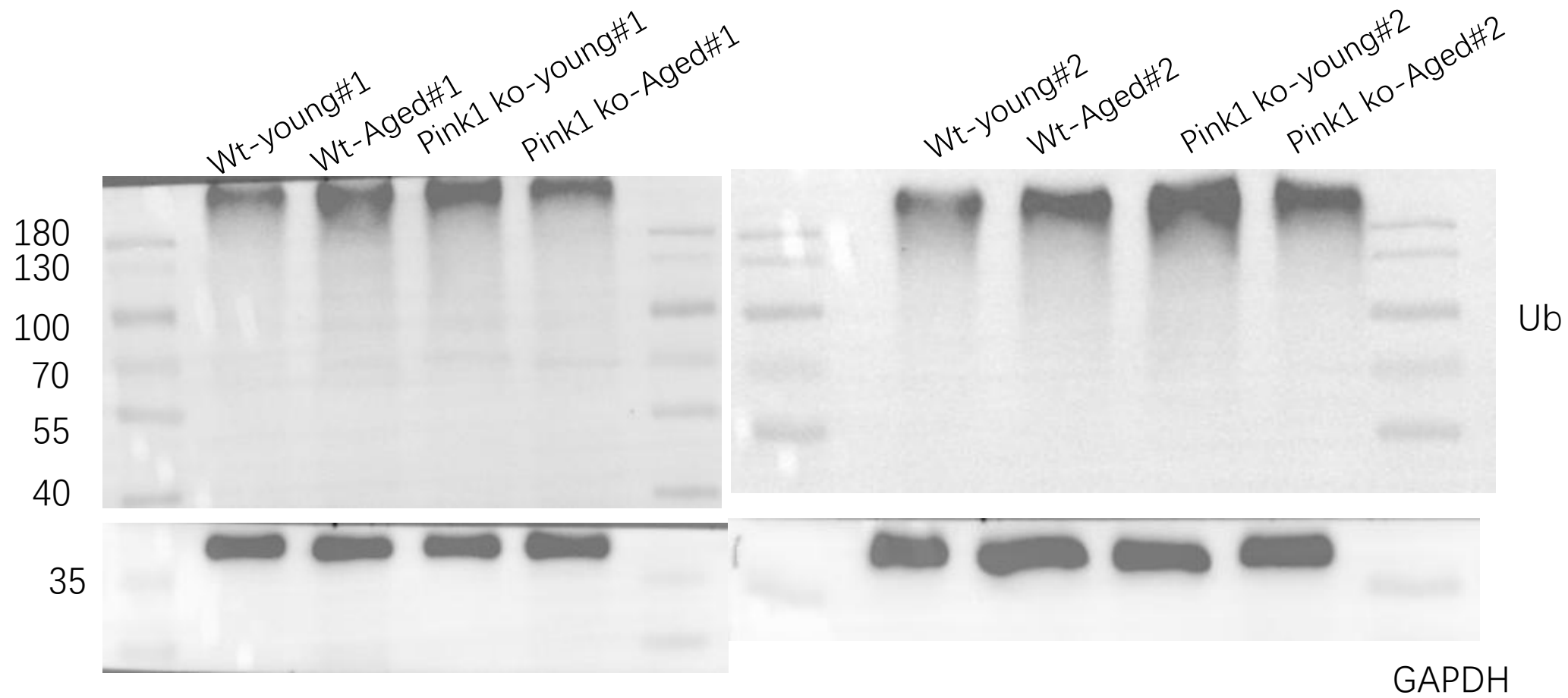

Supplement: Figure 4—source data 9. [file elife-103945-fig4-data9.pdf]

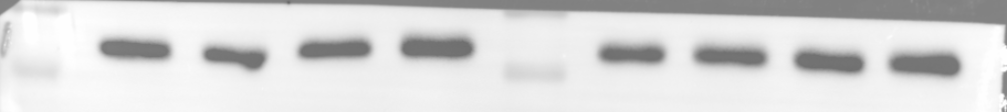

Supplement: Figure 4—source data 10. [file elife-103945-fig4-data10.zip › GAPDH-1.tif]

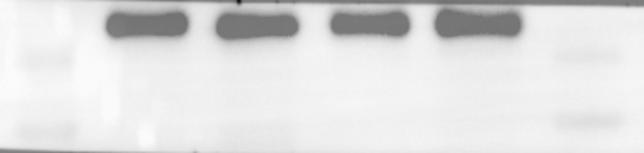

Supplement: Figure 4—source data 10. [file elife-103945-fig4-data10.zip › GAPDH-2.tif]

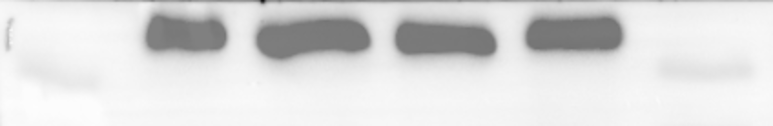

Supplement: Figure 4—source data 10. [file elife-103945-fig4-data10.zip › GAPDH-3.tif]

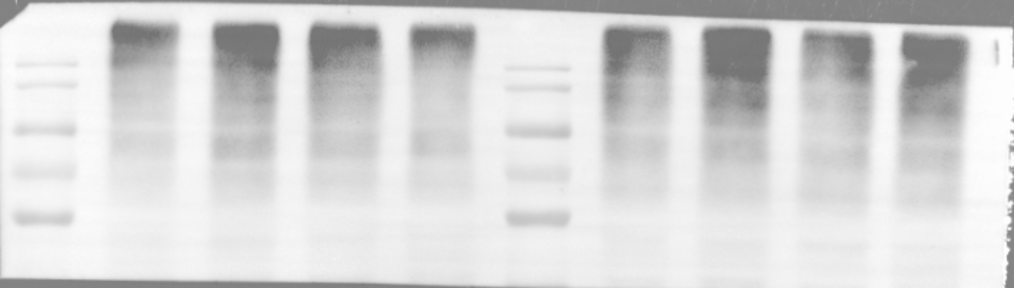

Supplement: Figure 4—source data 10. [file elife-103945-fig4-data10.zip › Ub-1.tif]

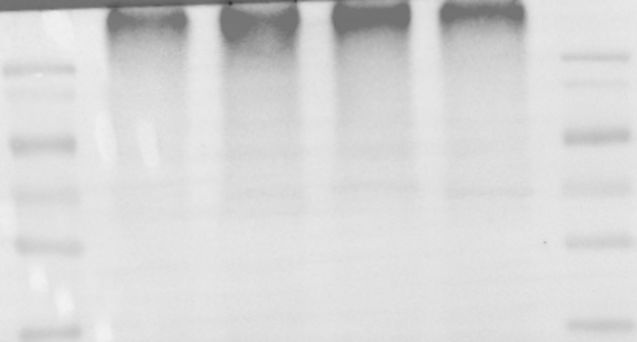

Supplement: Figure 4—source data 10. [file elife-103945-fig4-data10.zip › Ub-2.tif]

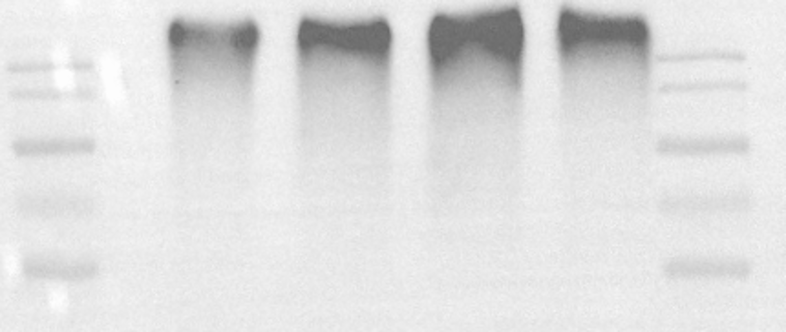

Supplement: Figure 4—source data 10. [file elife-103945-fig4-data10.zip › Ub-3.tif]

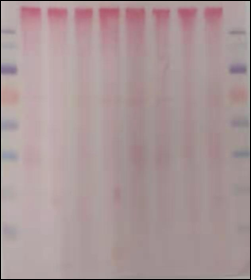

Supplement: Figure 4—source data 12. [file elife-103945-fig4-data12.zip › Ponceau staining-1.tif]

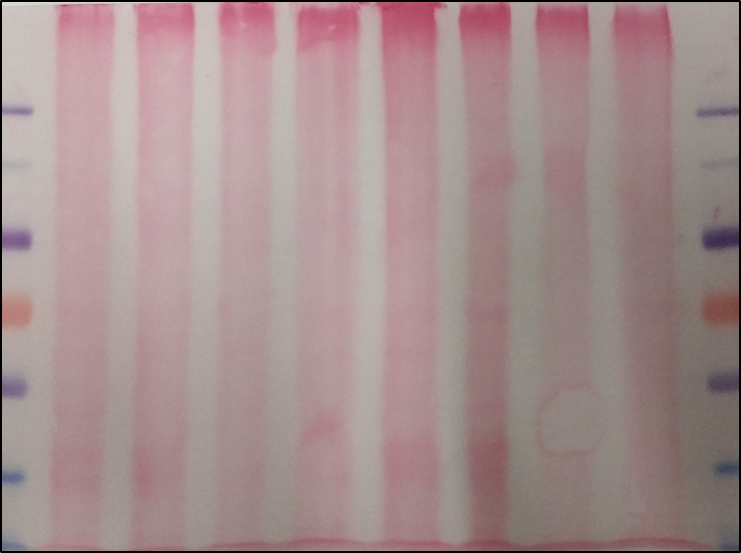

Supplement: Figure 4—source data 12. [file elife-103945-fig4-data12.zip › Ponceau staining-2.tif]

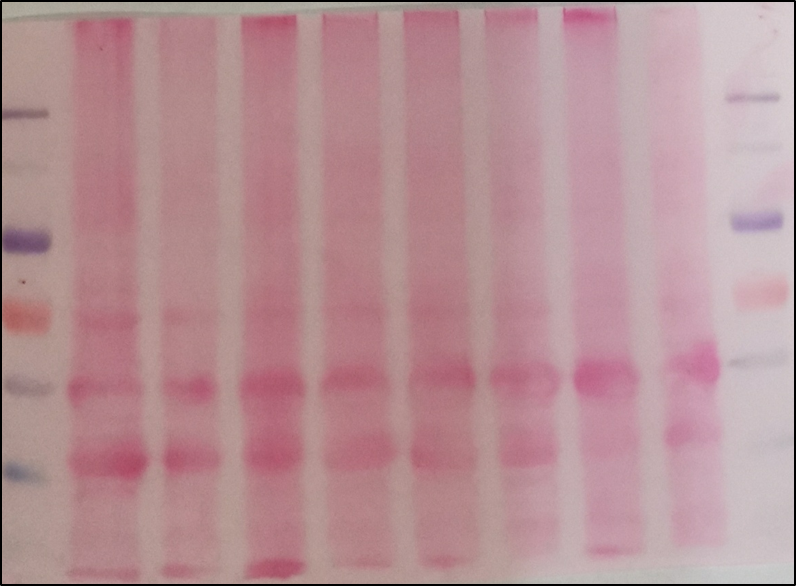

Supplement: Figure 4—source data 12. [file elife-103945-fig4-data12.zip › Ponceau staining-3.tif]

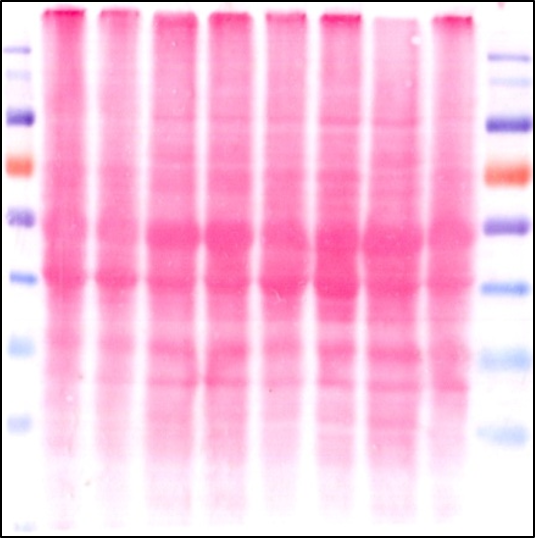

Supplement: Figure 4—source data 12. [file elife-103945-fig4-data12.zip › Ponceau staining-4.tif]

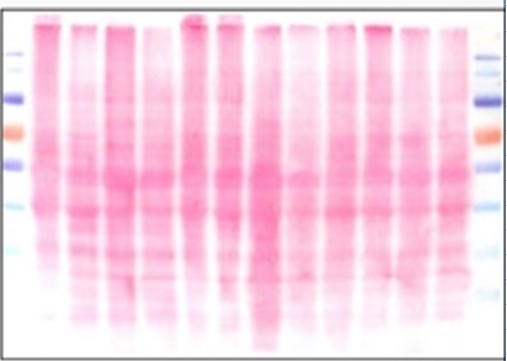

Supplement: Figure 4—source data 12. [file elife-103945-fig4-data12.zip › Ponceau staining-5.tif]

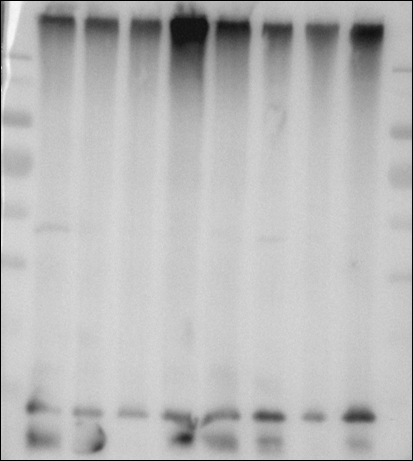

Supplement: Figure 4—source data 12. [file elife-103945-fig4-data12.zip › Ub-1.tif]
