# Supplementary material for: Elevated ubiquitin phosphorylation by PINK1 contributes to proteasomal impairment and promotes neurodegeneration
Source: eLife. 2025 Jul 31;14:RP103945. doi: 10.7554/eLife.103945 (PMC12313235; doi:10.7554/eLife.103945)
Supplement: Figure 4—source data 11. [file elife-103945-fig4-data11.pdf]

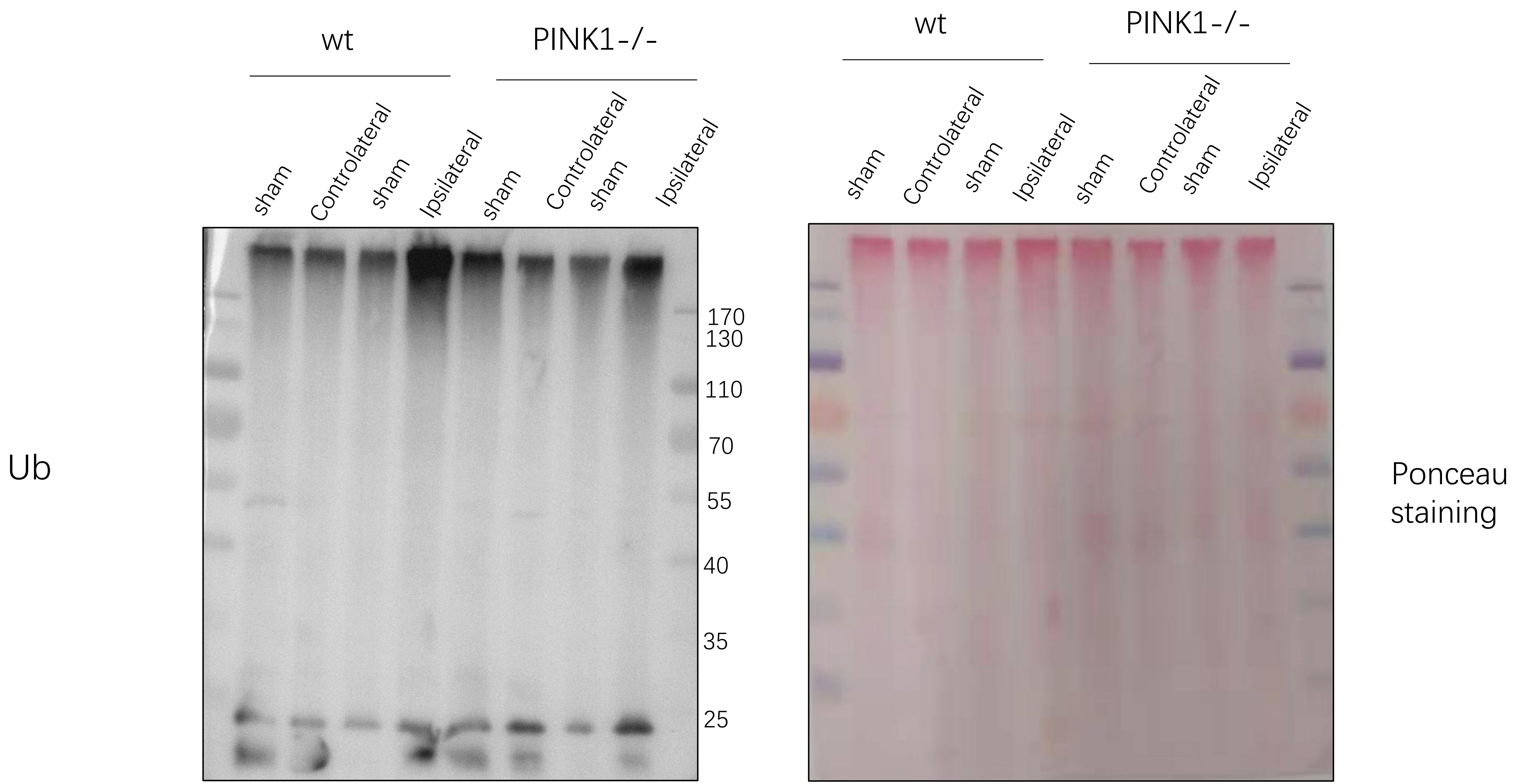

wt
PINK1-/-
sham
Controlateral sham
Ipsilateral
sham
Controlateral sham
Ipsilateral
170
130
110
70
55
40
35
25
Ub
wt
PINK1-/-
sham
Controlateral sham
Ipsilateral
sham
Controlateral sham
Ipsilateral
Ponceau staining

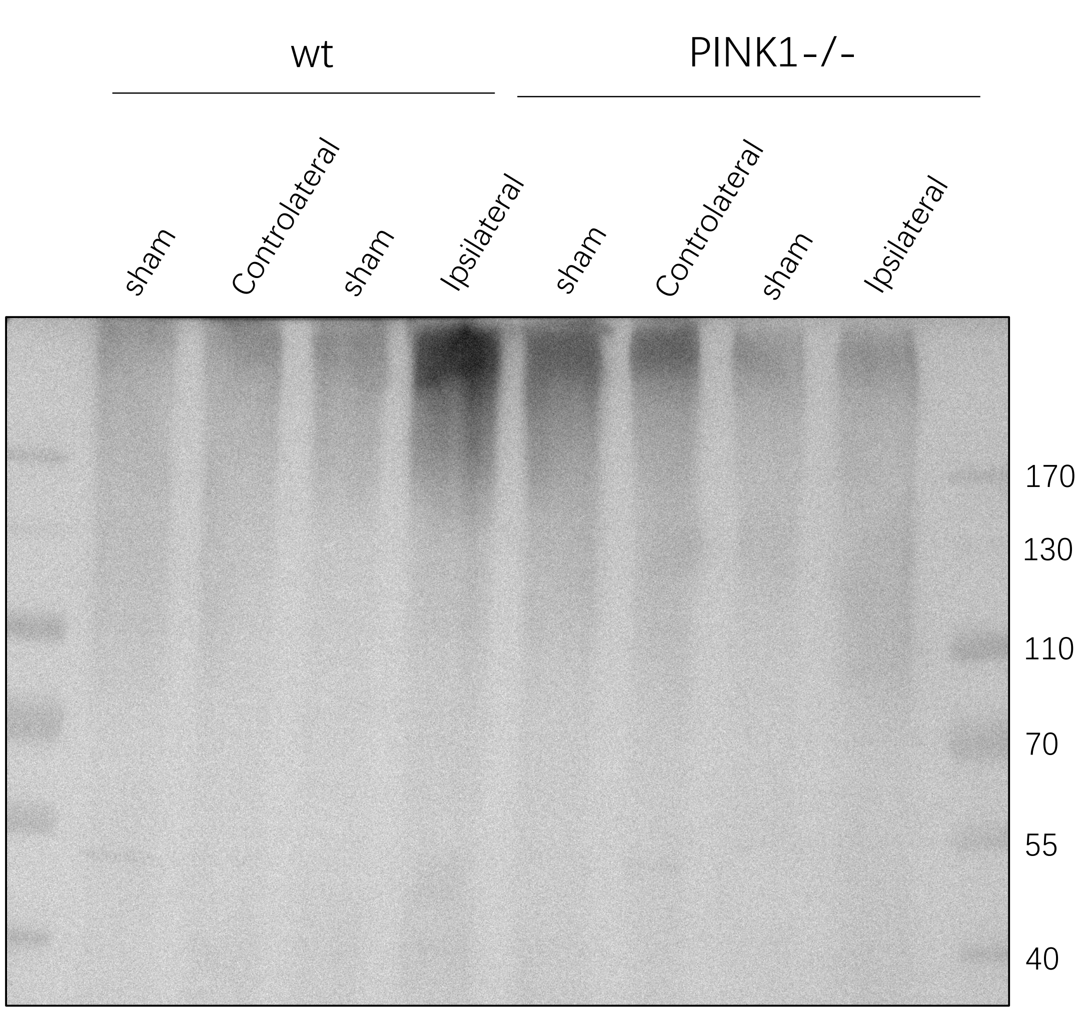

Ub

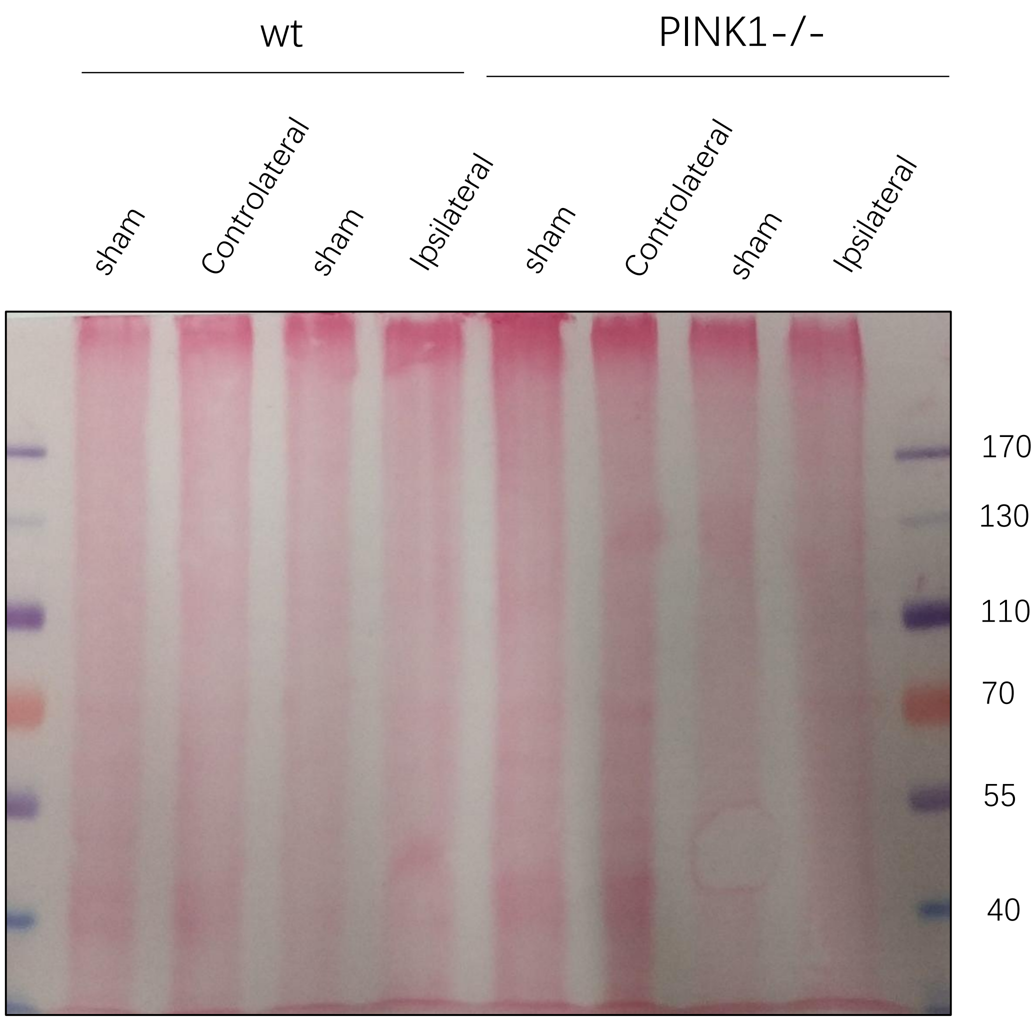

Ponceau staining

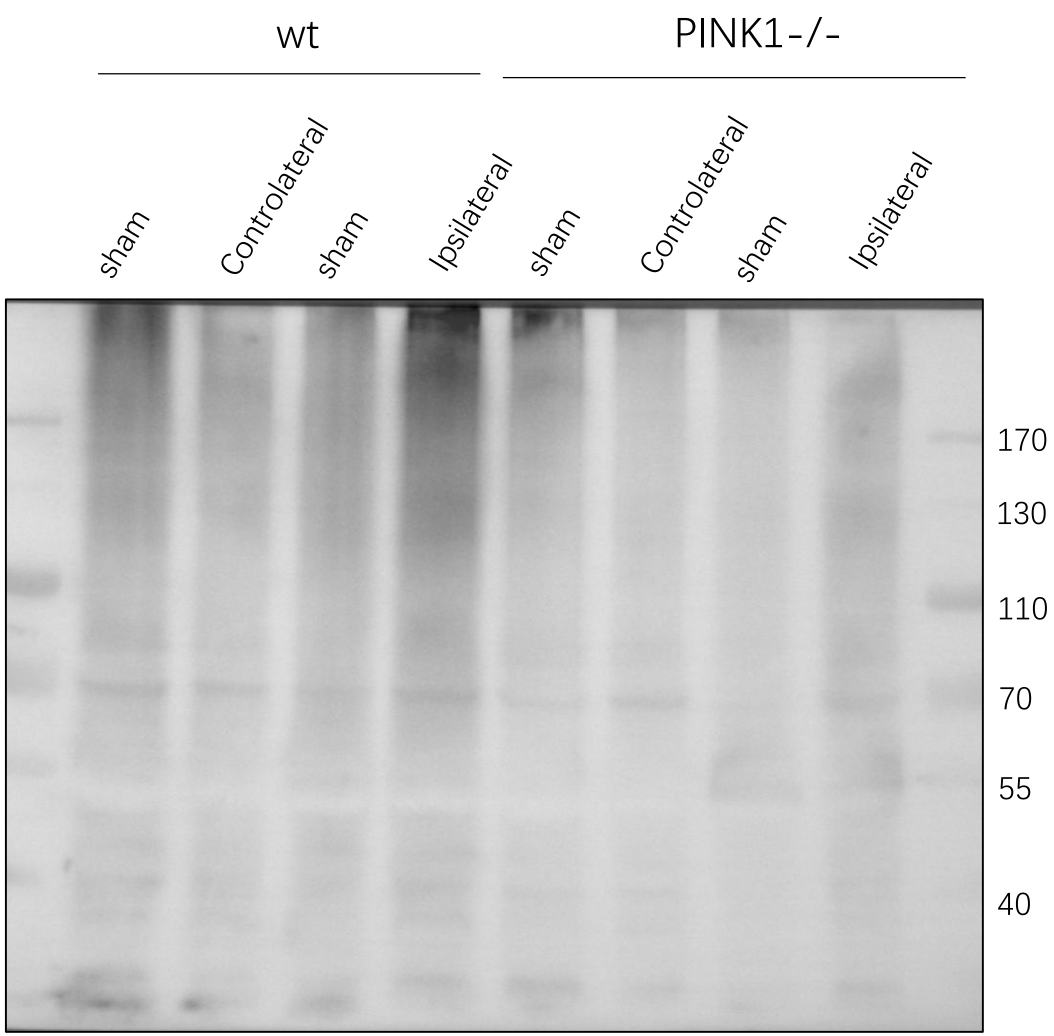

Ub

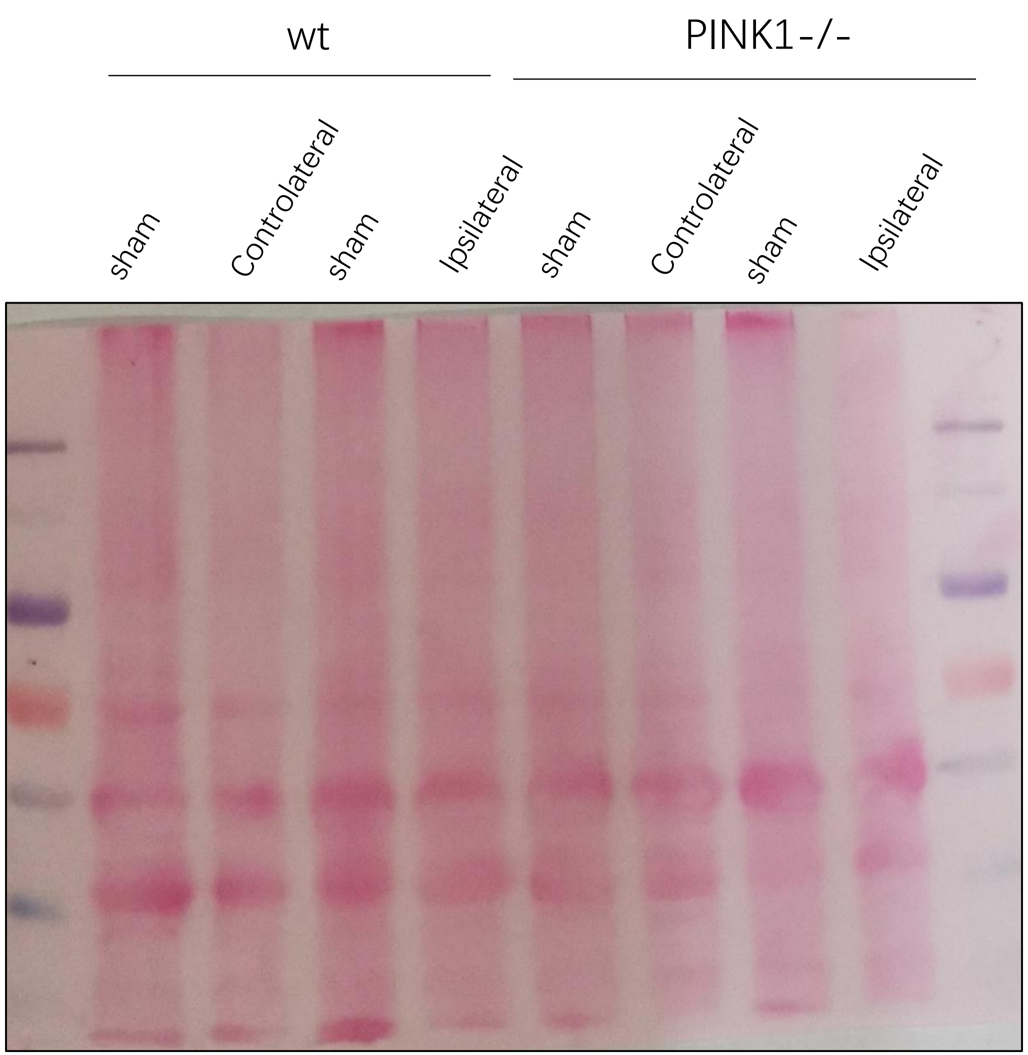

Ponceau staining

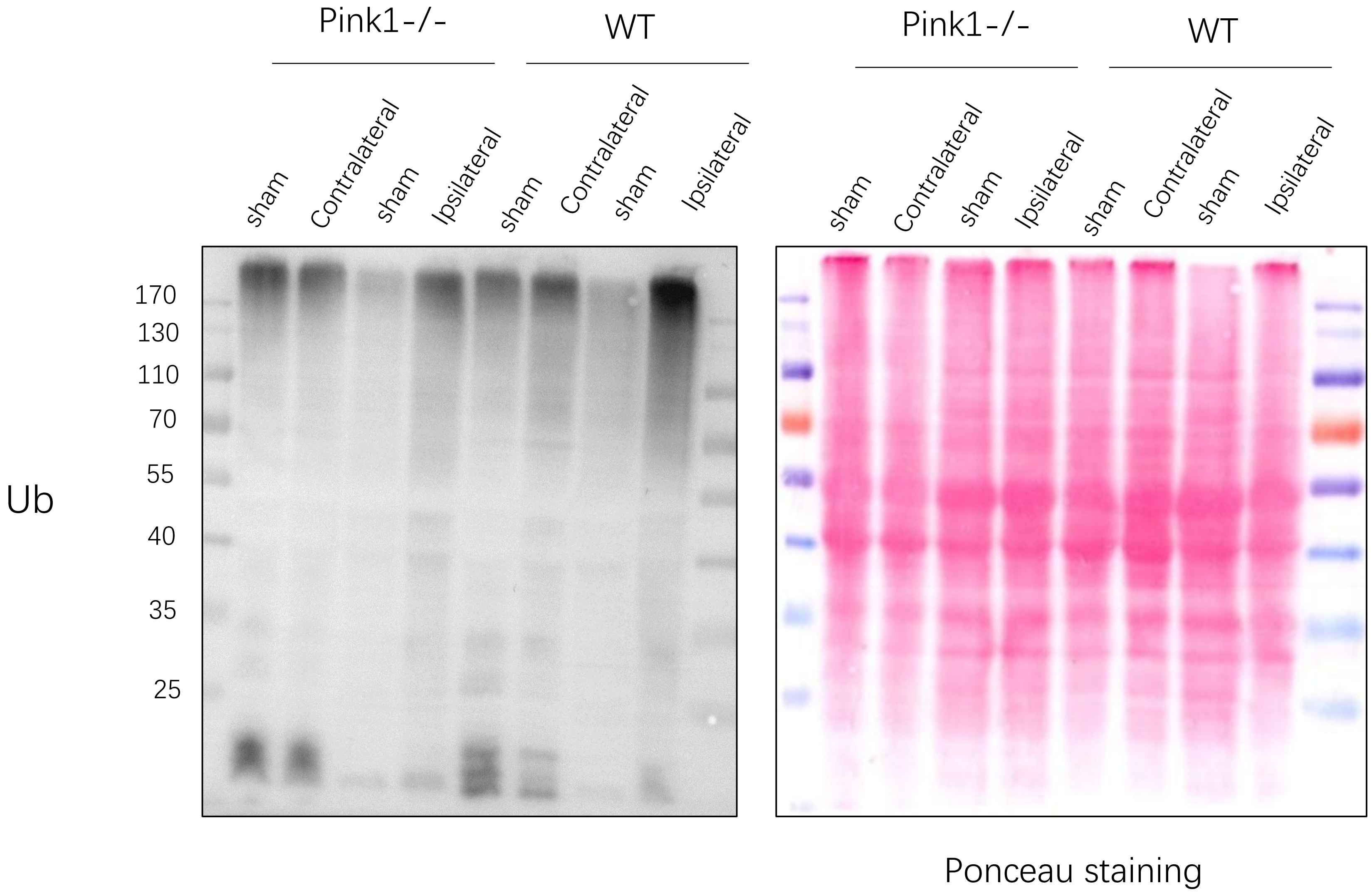
Pink1-/-
WT
sham
Contralateral
sham
Ipsilateral
sham
Contralateral
sham
Ipsilateral
Ub
170
130
110
70
55
40
35
25
Pink1-/-
WT
sham
Contralateral
sham
Ipsilateral
sham
Contralateral
sham
Ipsilateral
Ponceau staining

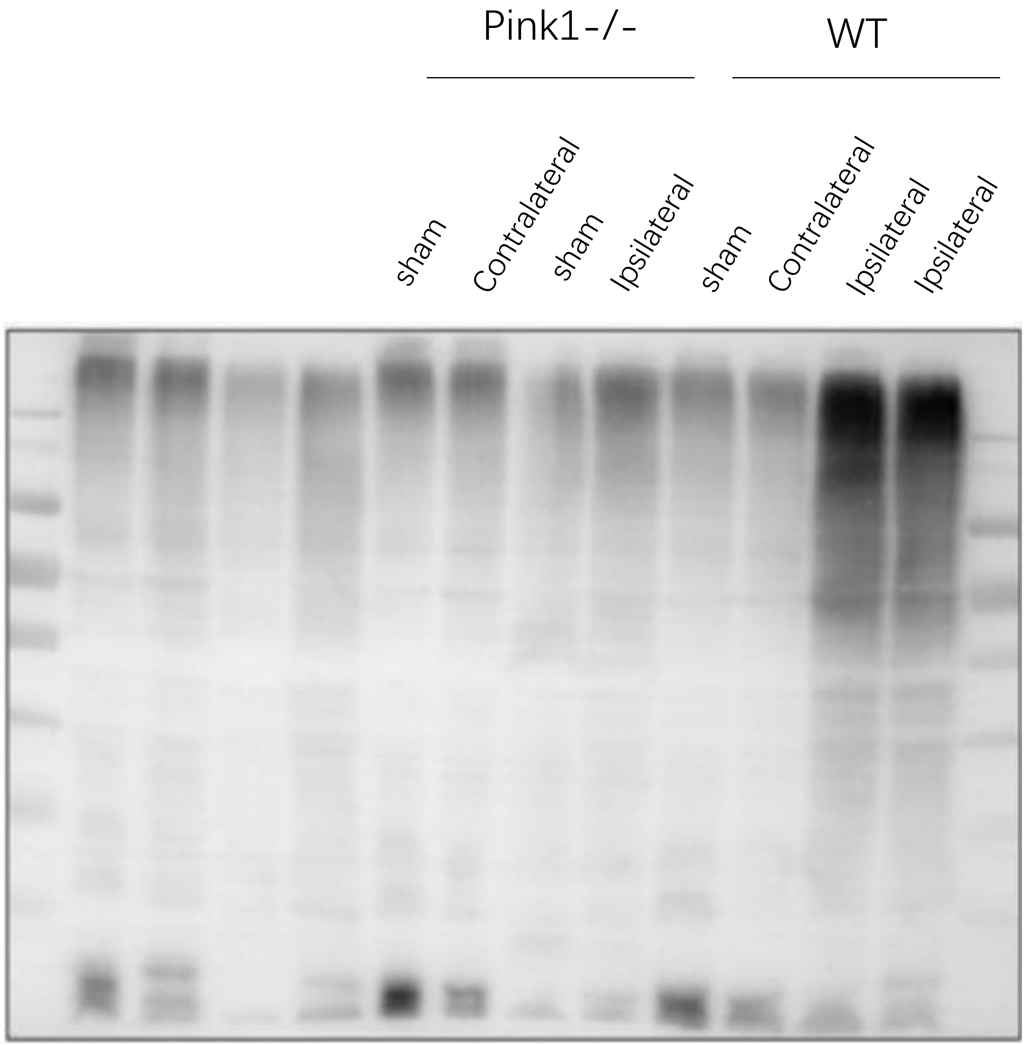

Ub

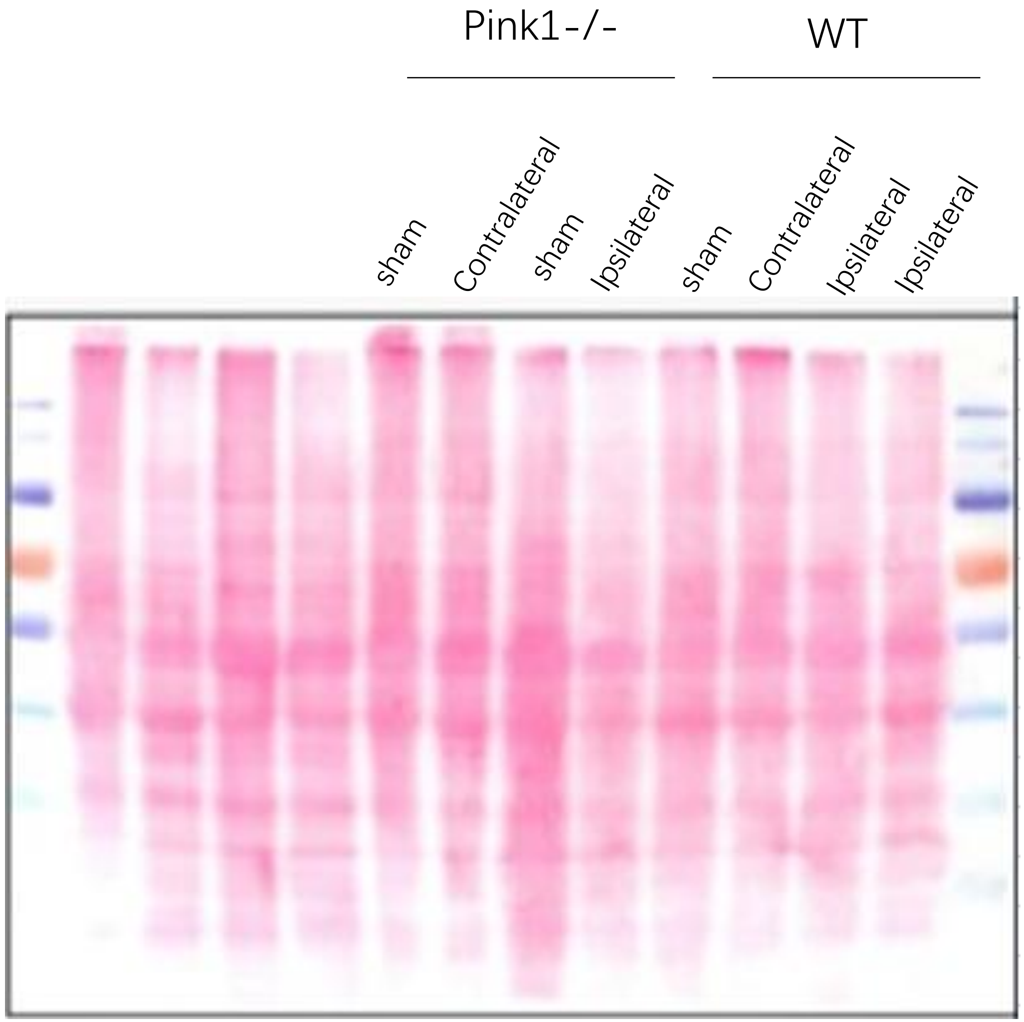

Ponceau staining
