# Supplementary figures and images for: Elevated ubiquitin phosphorylation by PINK1 contributes to proteasomal impairment and promotes neurodegeneration (part 2 of 2)
Source: eLife. 2025 Jul 31;14:RP103945. doi: 10.7554/eLife.103945 (PMC12313235; doi:10.7554/eLife.103945)

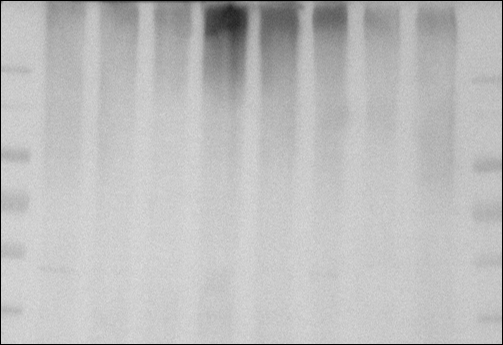

Supplement: Figure 4—source data 12. [file elife-103945-fig4-data12.zip › Ub-2.tif]

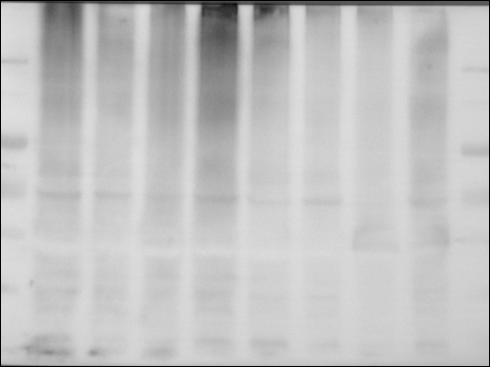

Supplement: Figure 4—source data 12. [file elife-103945-fig4-data12.zip › Ub-3.tif]

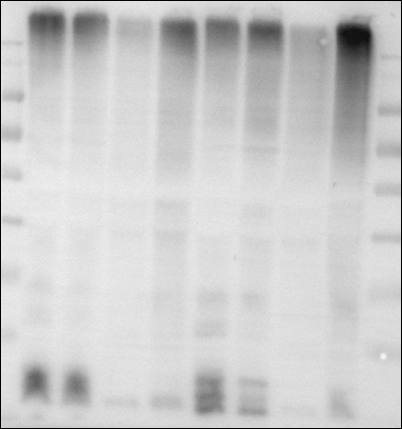

Supplement: Figure 4—source data 12. [file elife-103945-fig4-data12.zip › Ub-4.tif]

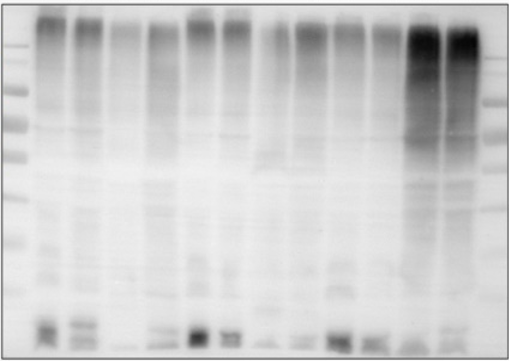

Supplement: Figure 4—source data 12. [file elife-103945-fig4-data12.zip › Ub-5.tif]

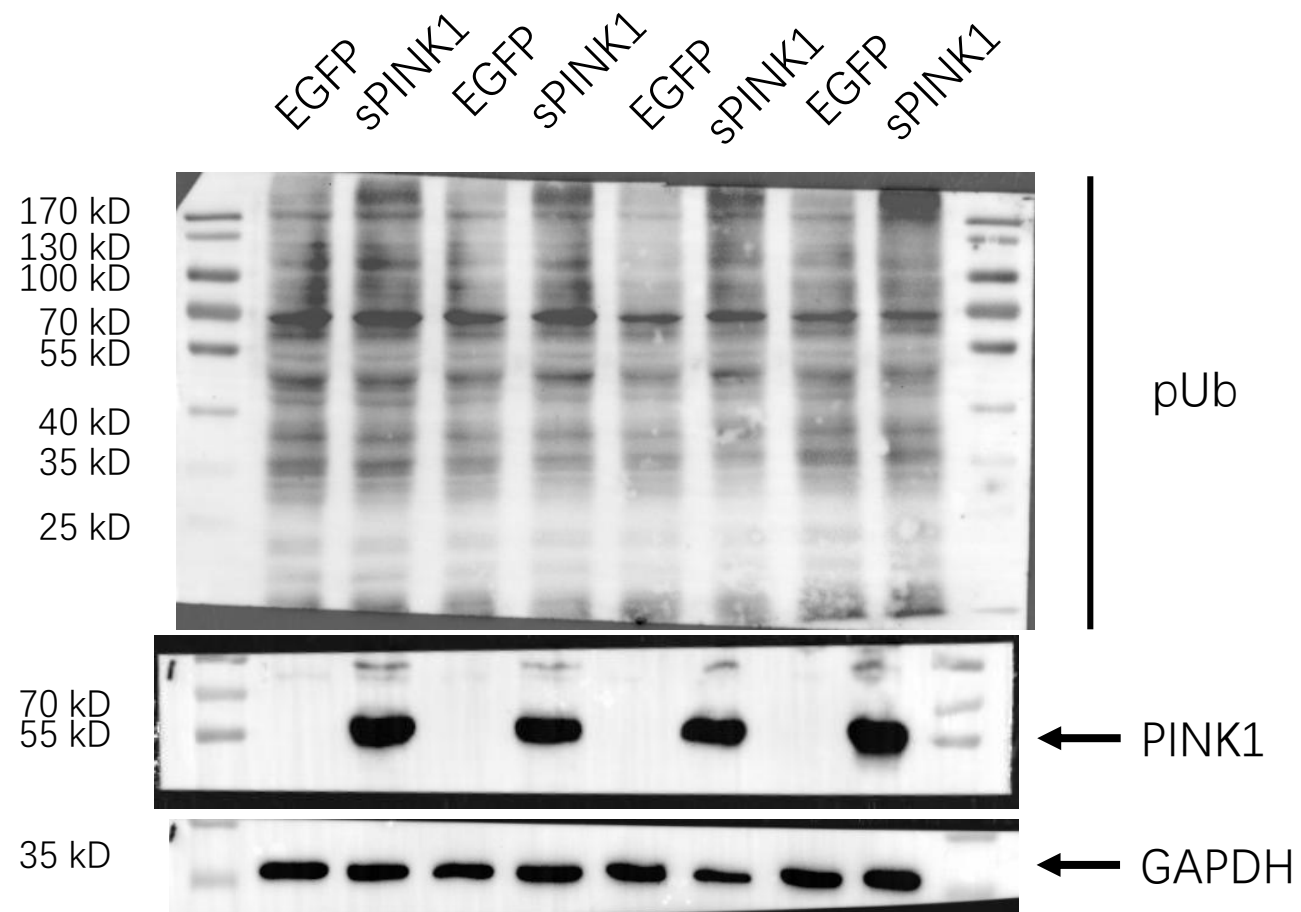

Supplement: Figure 6—source data 1. [file elife-103945-fig6-data1.pdf]

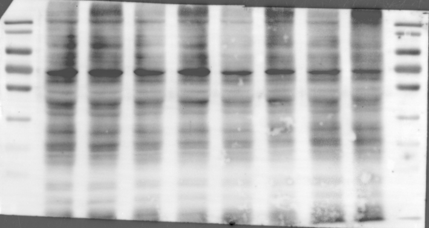

Supplement: Figure 6—source data 2. [file elife-103945-fig6-data2.zip › pUb.tif]

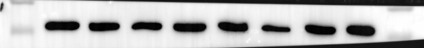

Supplement: Figure 6—source data 2. [file elife-103945-fig6-data2.zip › GAPDH.tif]

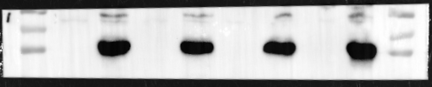

Supplement: Figure 6—source data 2. [file elife-103945-fig6-data2.zip › PINK1.tif]

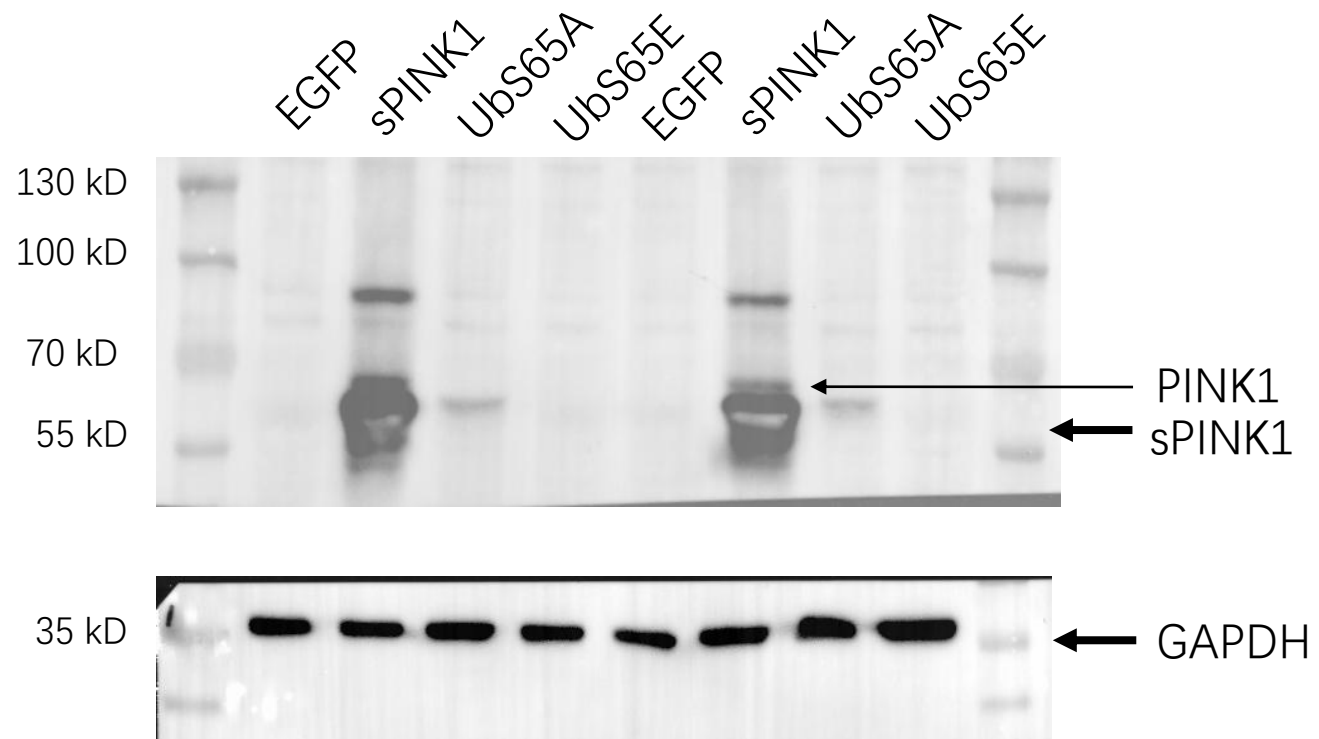

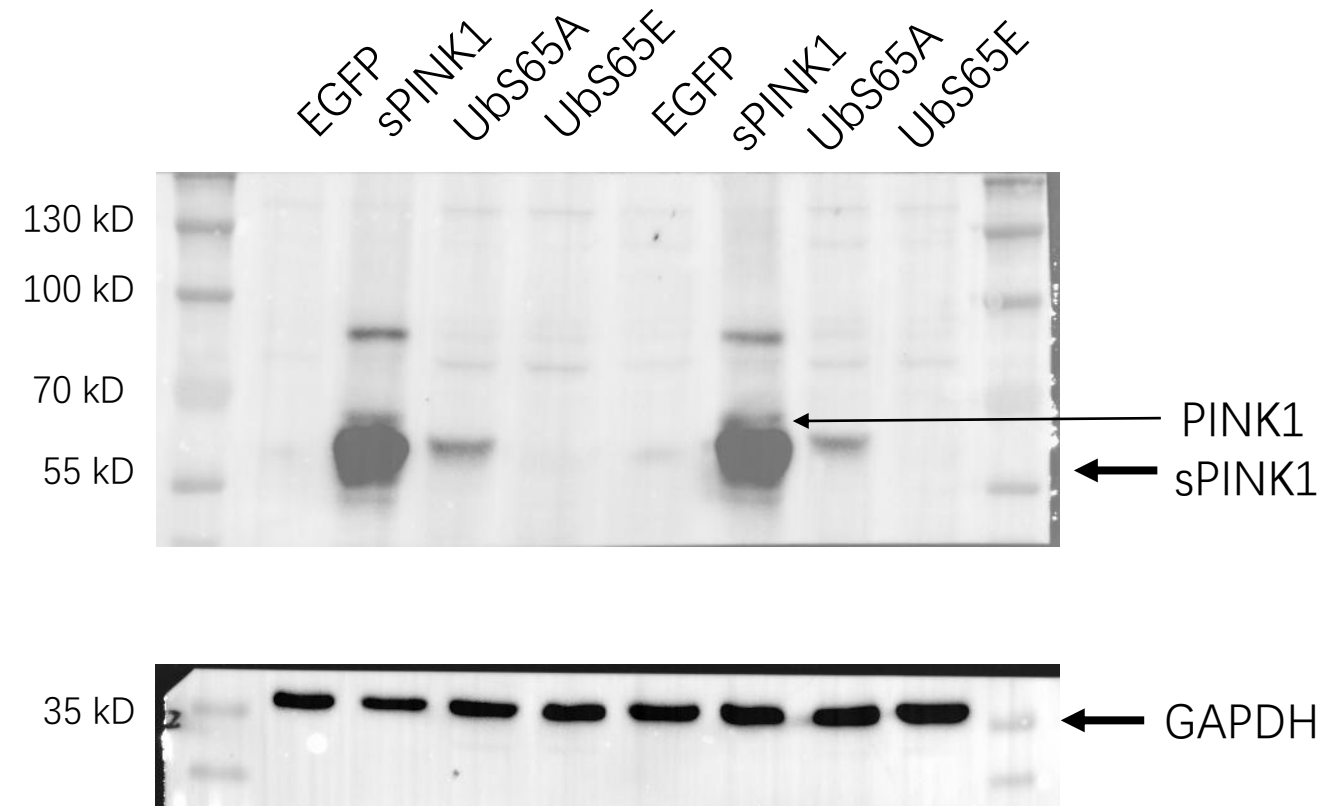

Supplement: Figure 6—source data 3. [file elife-103945-fig6-data3.pdf]

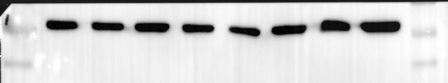

Supplement: Figure 6—source data 4. [file elife-103945-fig6-data4.zip › GAPDH-1.tif]

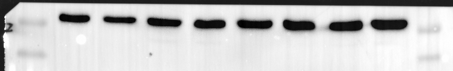

Supplement: Figure 6—source data 4. [file elife-103945-fig6-data4.zip › GAPDH-2.tif]

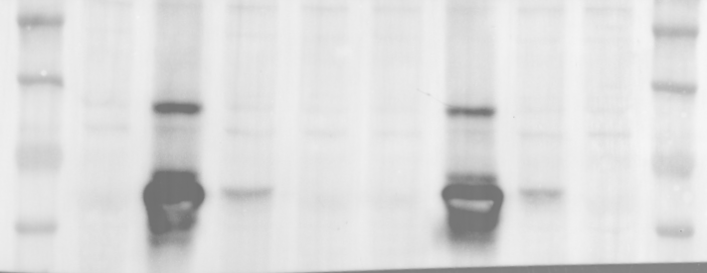

Supplement: Figure 6—source data 4. [file elife-103945-fig6-data4.zip › PINK1-1.tif]

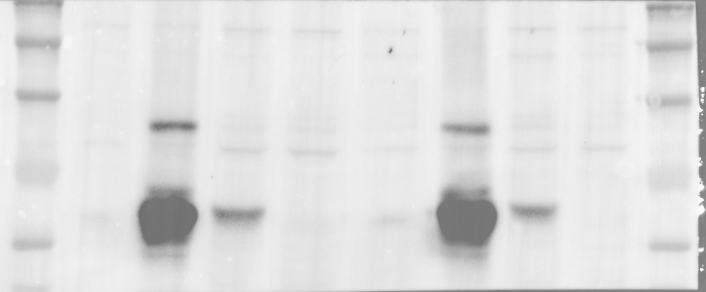

Supplement: Figure 6—source data 4. [file elife-103945-fig6-data4.zip › PINK1-2.tif]

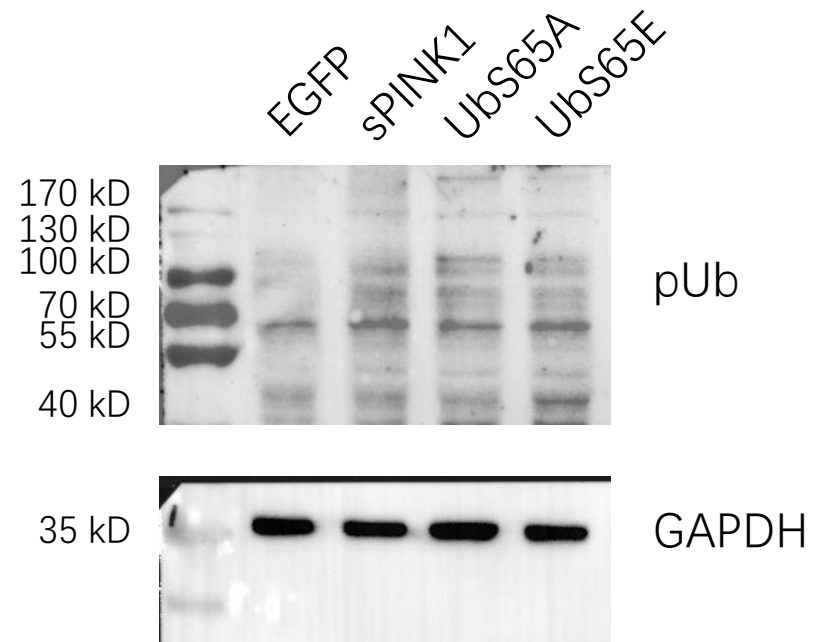

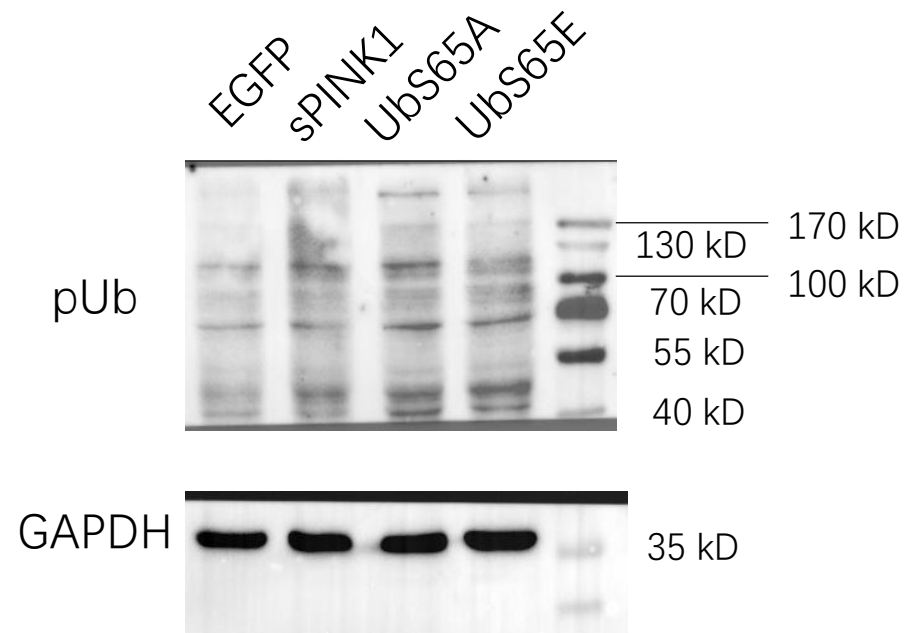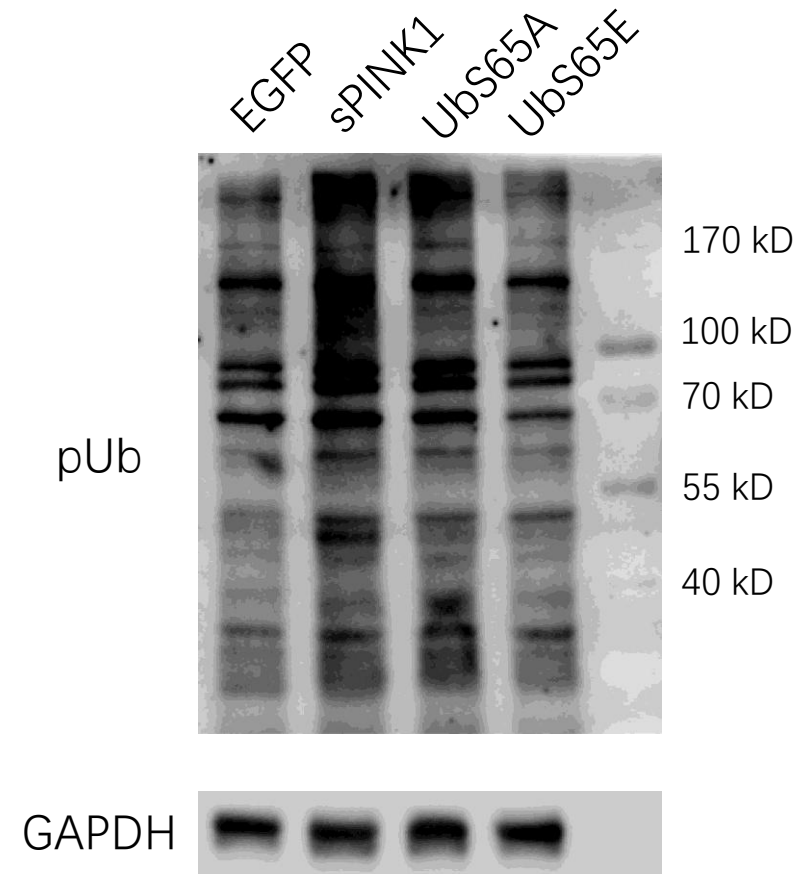

EGFP SPINK1 UbS65A UbS65E EGFP SPINK1 UbS65A UbS65E

130 kD

100 kD

70 kD

55 kD

40 kD

35 kD

pUb

GAPDH

Supplement: Figure 6—source data 5. [file elife-103945-fig6-data5.pdf]

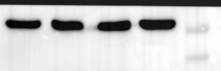

Supplement: Figure 6—source data 6. [file elife-103945-fig6-data6.zip › GAPDH-1.tif]

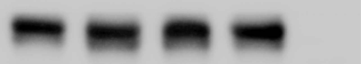

Supplement: Figure 6—source data 6. [file elife-103945-fig6-data6.zip › GAPDH-2.tif]

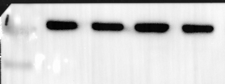

Supplement: Figure 6—source data 6. [file elife-103945-fig6-data6.zip › GAPDH-3.tif]

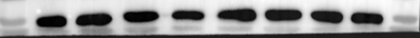

Supplement: Figure 6—source data 6. [file elife-103945-fig6-data6.zip › GAPDH-4.tif]

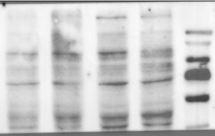

Supplement: Figure 6—source data 6. [file elife-103945-fig6-data6.zip › pUb-1.tif]

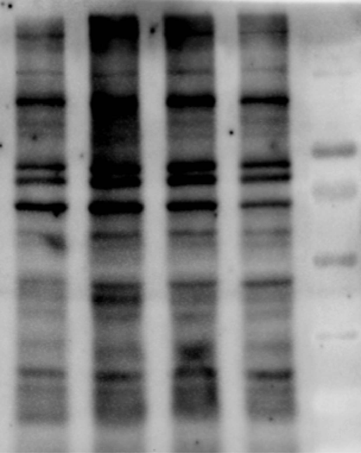

Supplement: Figure 6—source data 6. [file elife-103945-fig6-data6.zip › pUb-2.tif]

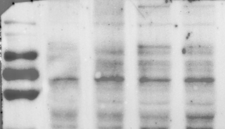

Supplement: Figure 6—source data 6. [file elife-103945-fig6-data6.zip › pUb-3.tif]

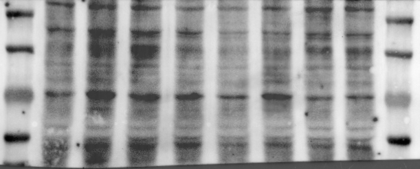

Supplement: Figure 6—source data 6. [file elife-103945-fig6-data6.zip › pUb-4.tif]

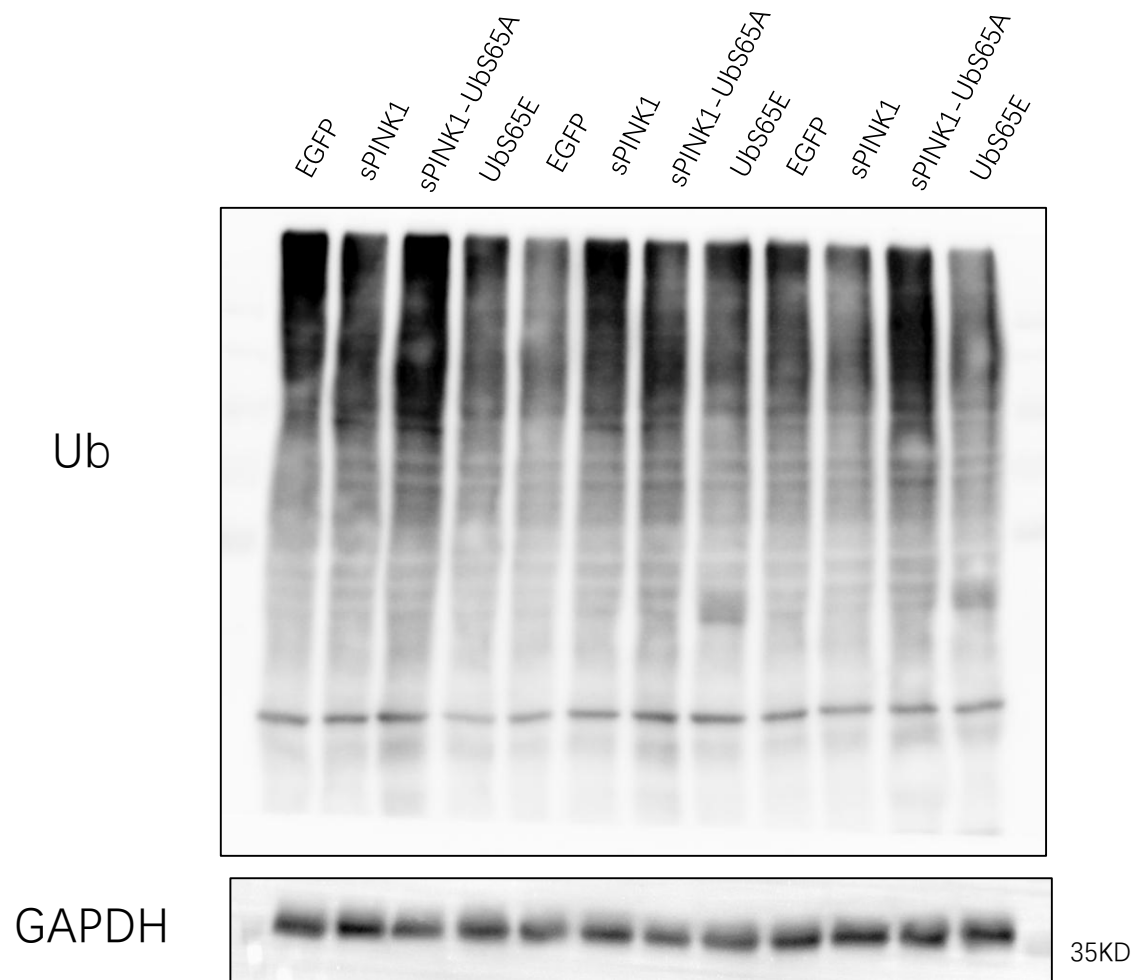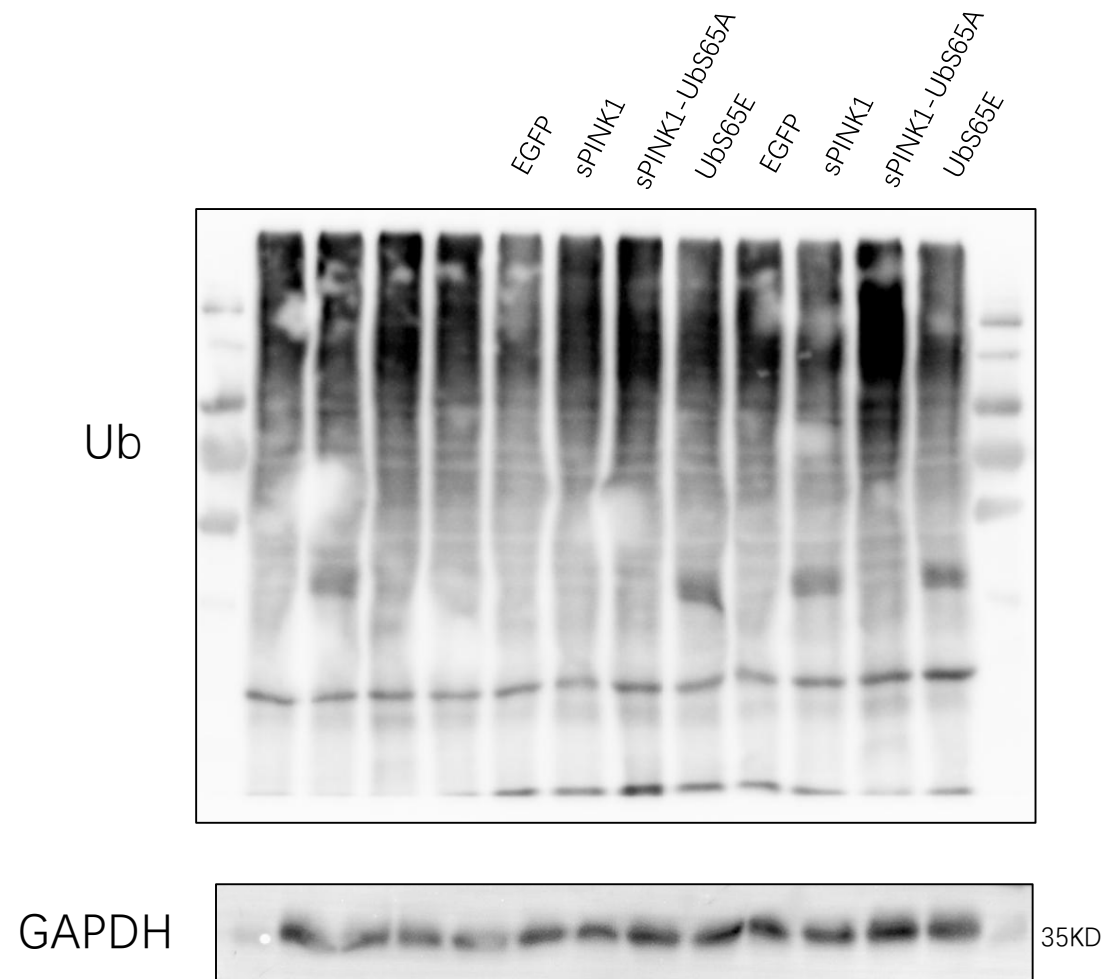

Ub

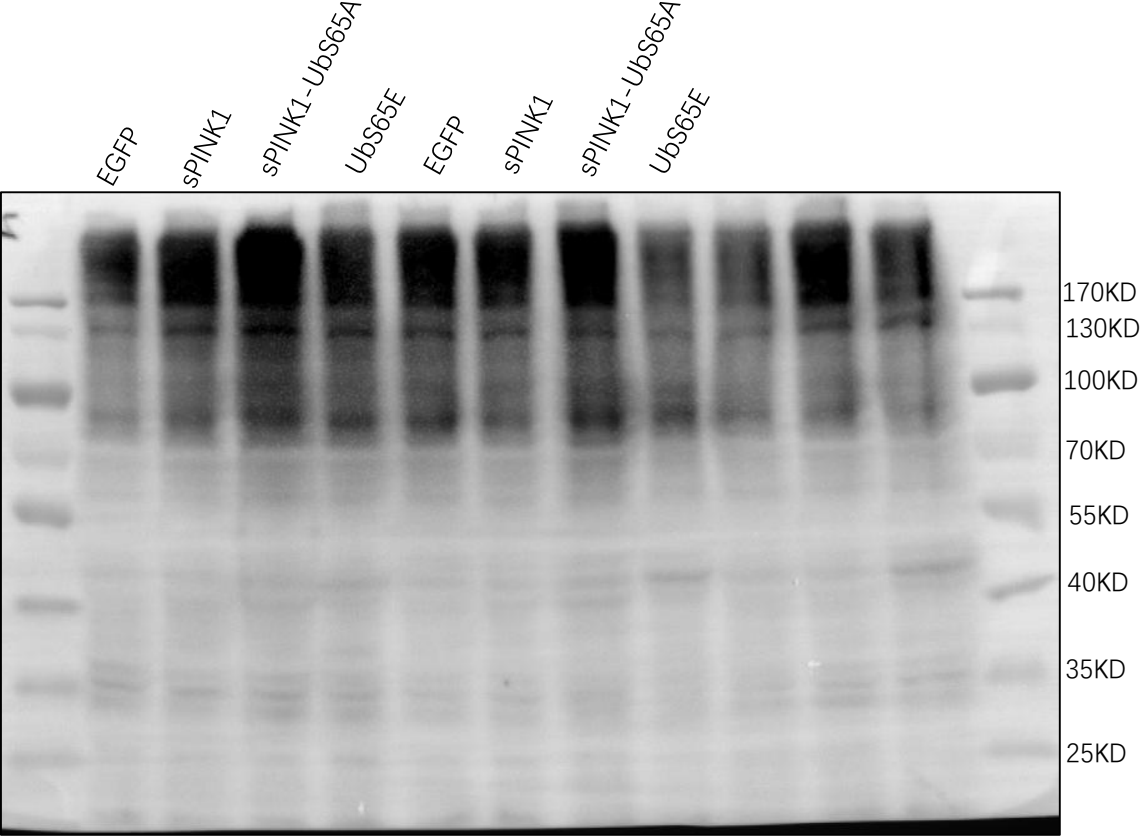

poncean staining

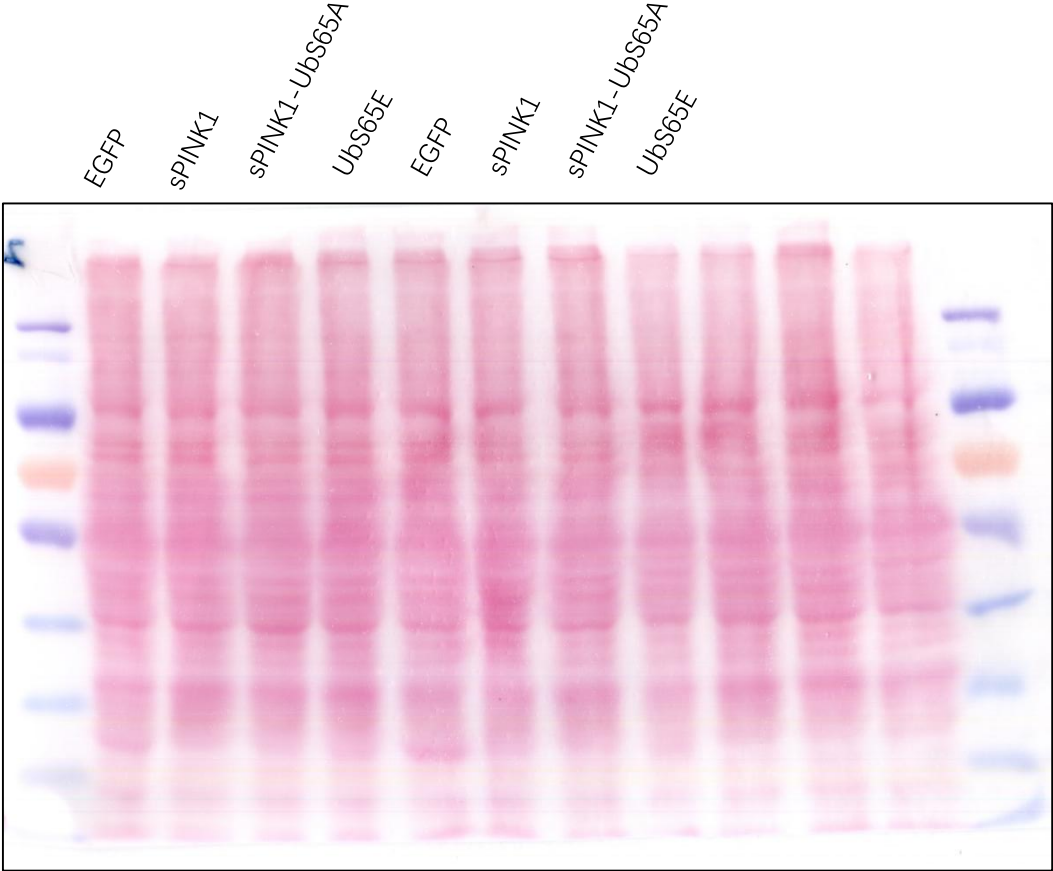

Supplement: Figure 6—source data 7. [file elife-103945-fig6-data7.pdf]

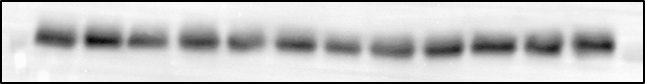

Supplement: Figure 6—source data 8. [file elife-103945-fig6-data8.zip › GAPDH-1.tif]

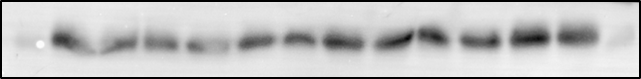

Supplement: Figure 6—source data 8. [file elife-103945-fig6-data8.zip › GAPDH-2.tif]

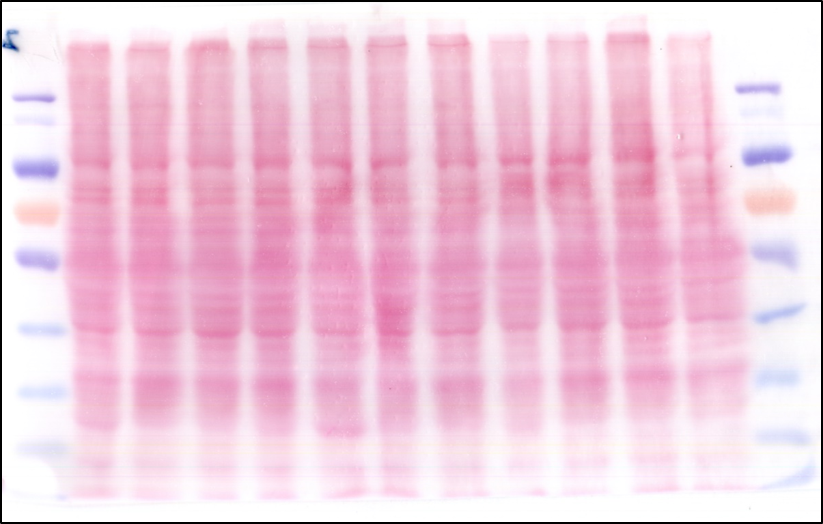

Supplement: Figure 6—source data 8. [file elife-103945-fig6-data8.zip › Ponceau staining.tif]

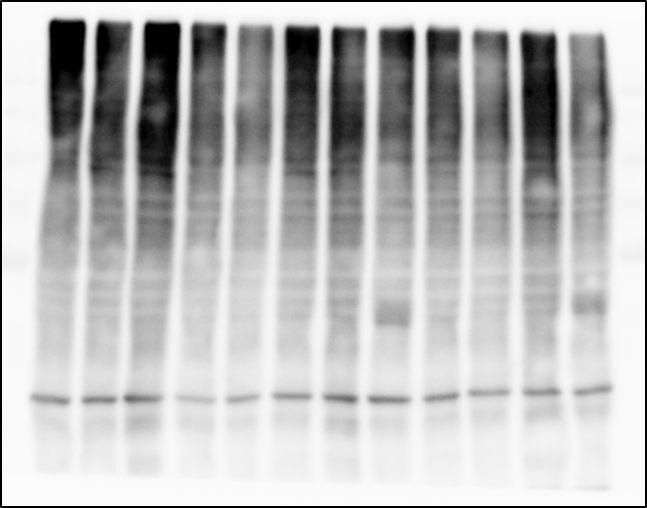

Supplement: Figure 6—source data 8. [file elife-103945-fig6-data8.zip › souble Ub-1.tif]

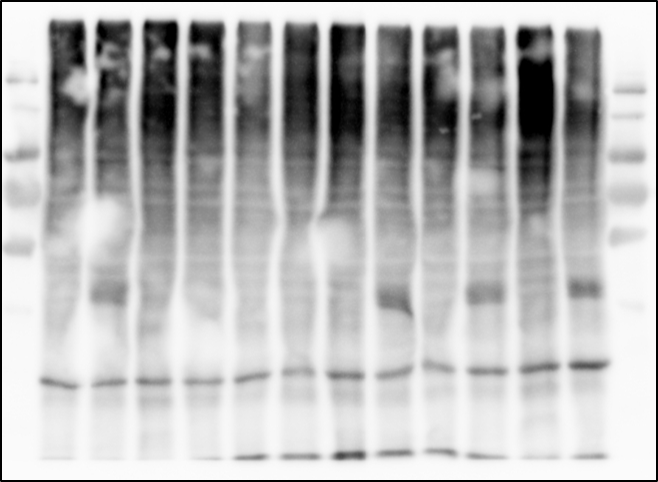

Supplement: Figure 6—source data 8. [file elife-103945-fig6-data8.zip › souble Ub-2.tif]

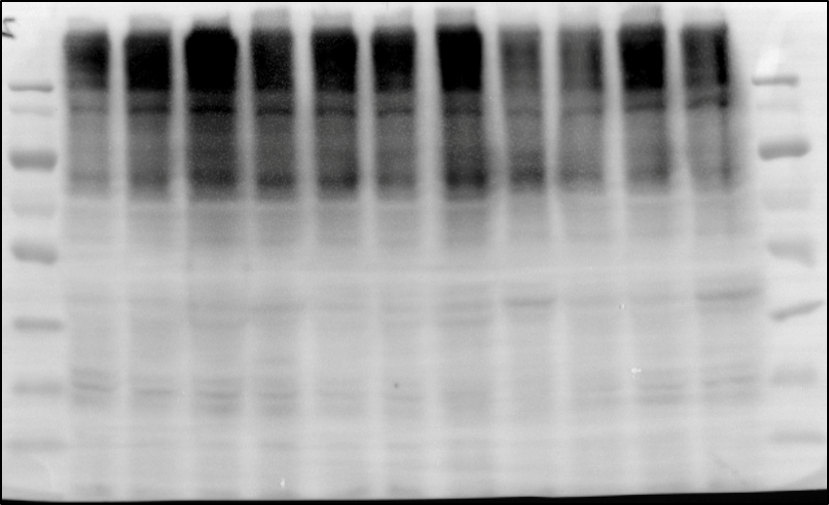

Supplement: Figure 6—source data 8. [file elife-103945-fig6-data8.zip › souble Ub-3.tif]

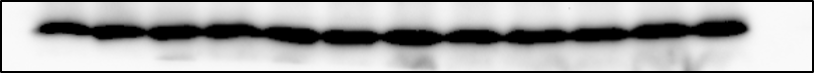

Supplement: Figure 6—source data 10. [file elife-103945-fig6-data10.zip › GAPDH-1.tif]

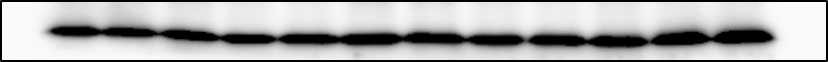

Supplement: Figure 6—source data 10. [file elife-103945-fig6-data10.zip › GAPDH-2.tif]

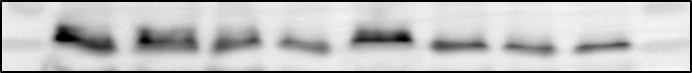

Supplement: Figure 6—source data 10. [file elife-103945-fig6-data10.zip › GAPDH-3.tif]

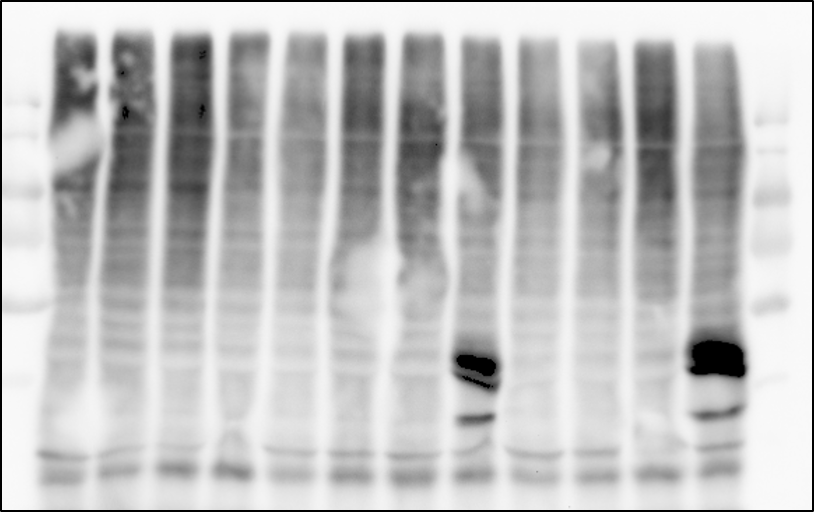

Supplement: Figure 6—source data 10. [file elife-103945-fig6-data10.zip › insoluble Ub-1.tif]

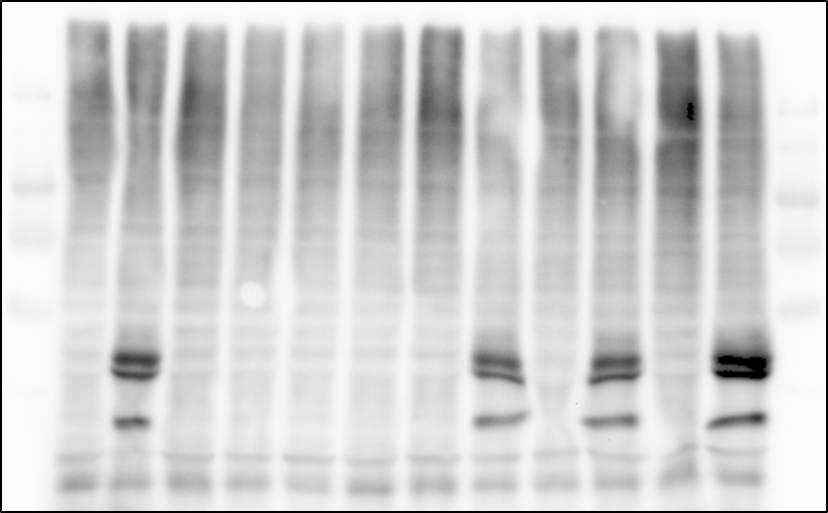

Supplement: Figure 6—source data 10. [file elife-103945-fig6-data10.zip › insoluble Ub-2.tif]

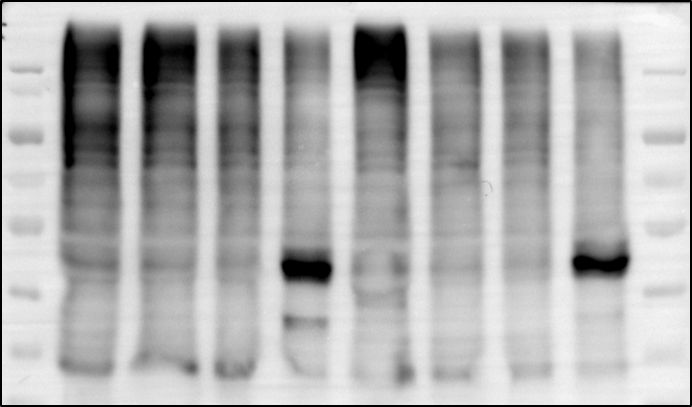

Supplement: Figure 6—source data 10. [file elife-103945-fig6-data10.zip › insoluble Ub-3.tif]

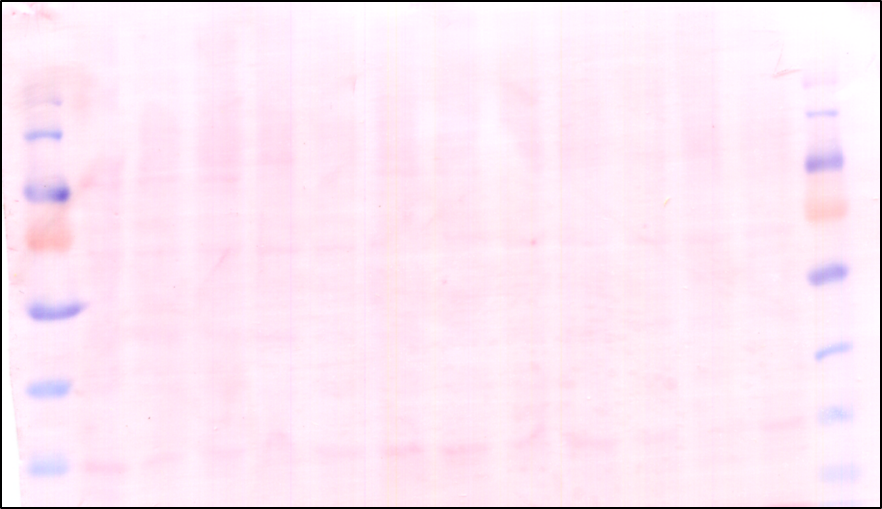

Supplement: Figure 6—source data 10. [file elife-103945-fig6-data10.zip › Ponceau staining-1.tif]

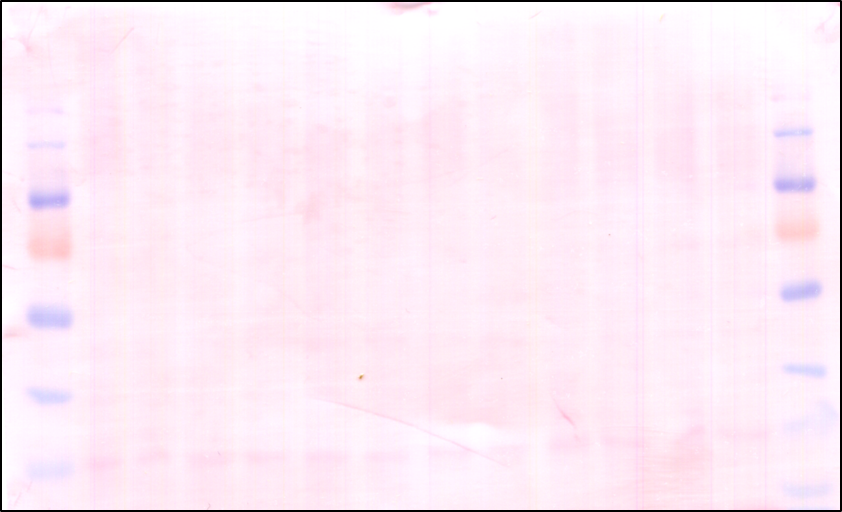

Supplement: Figure 6—source data 10. [file elife-103945-fig6-data10.zip › Ponceau staining-2.tif]

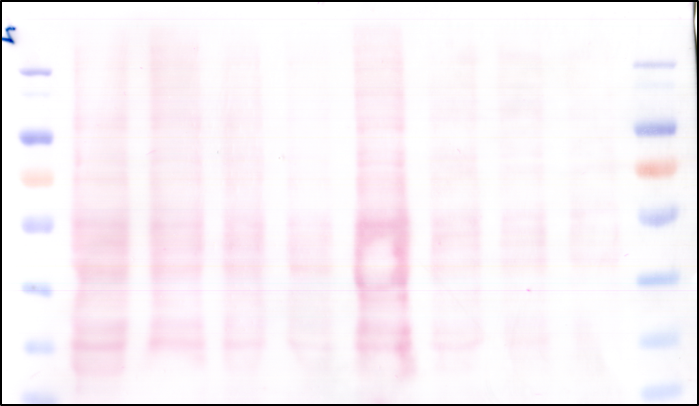

Supplement: Figure 6—source data 10. [file elife-103945-fig6-data10.zip › Ponceau staining-3.tif]

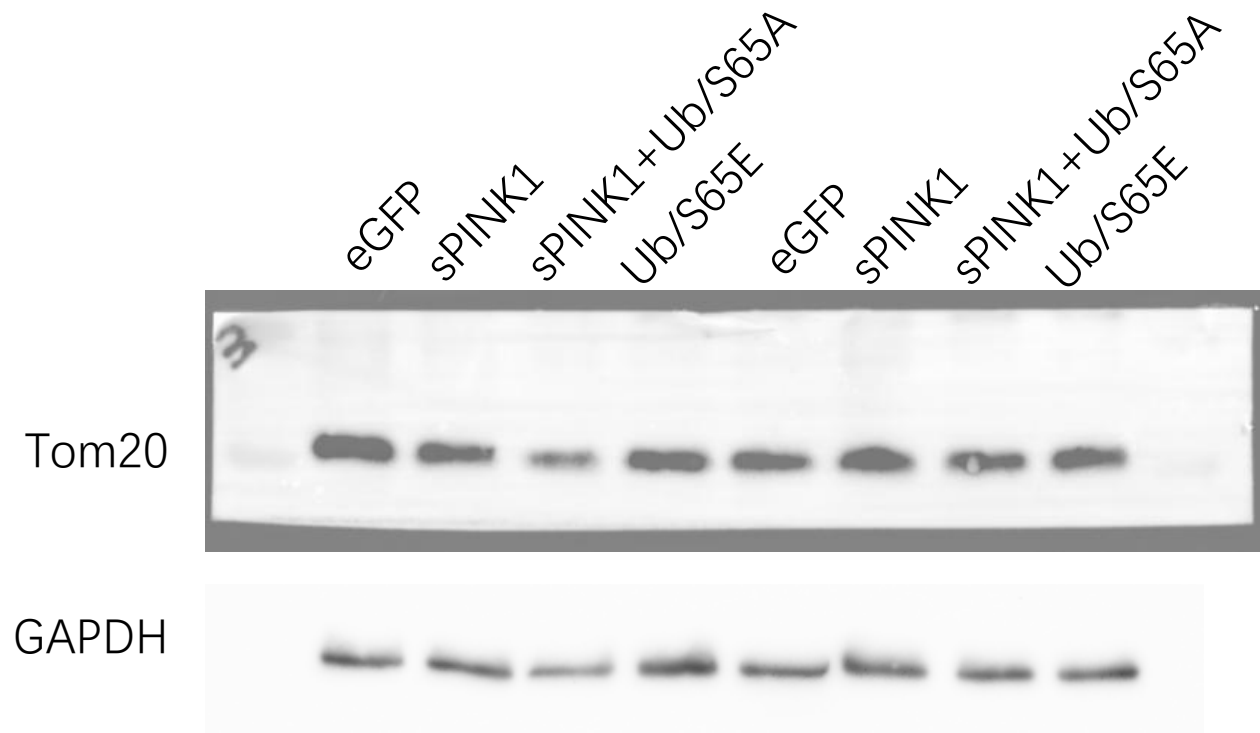

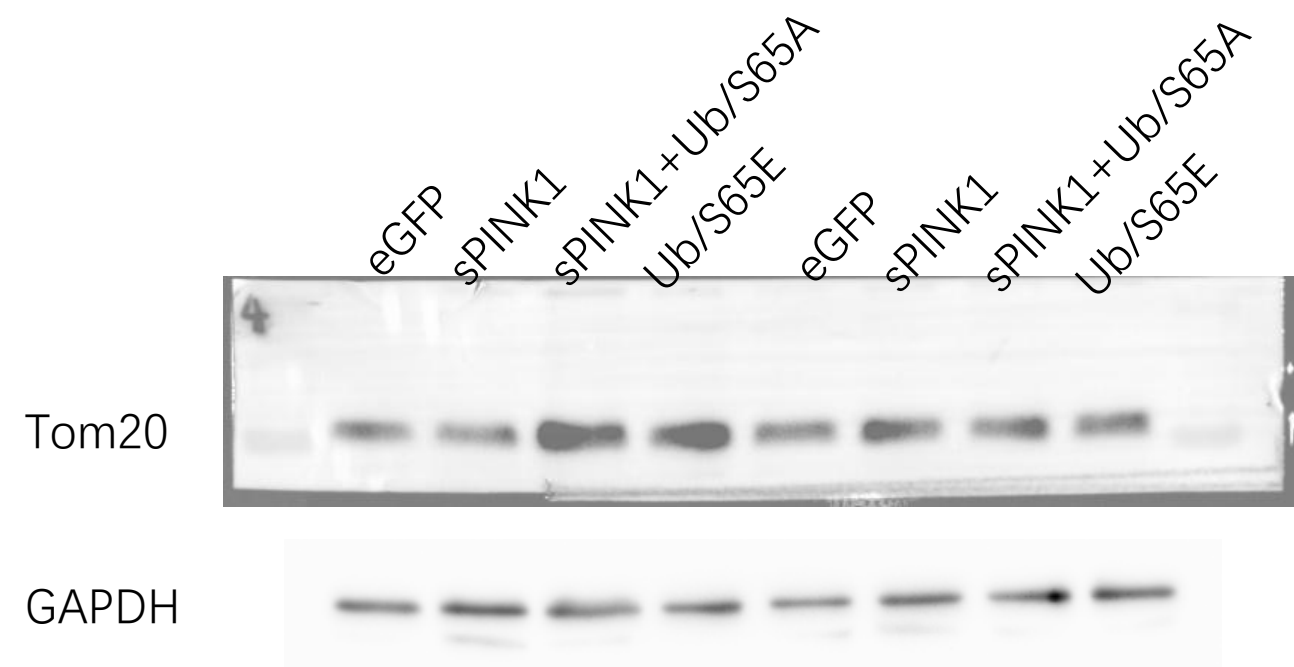

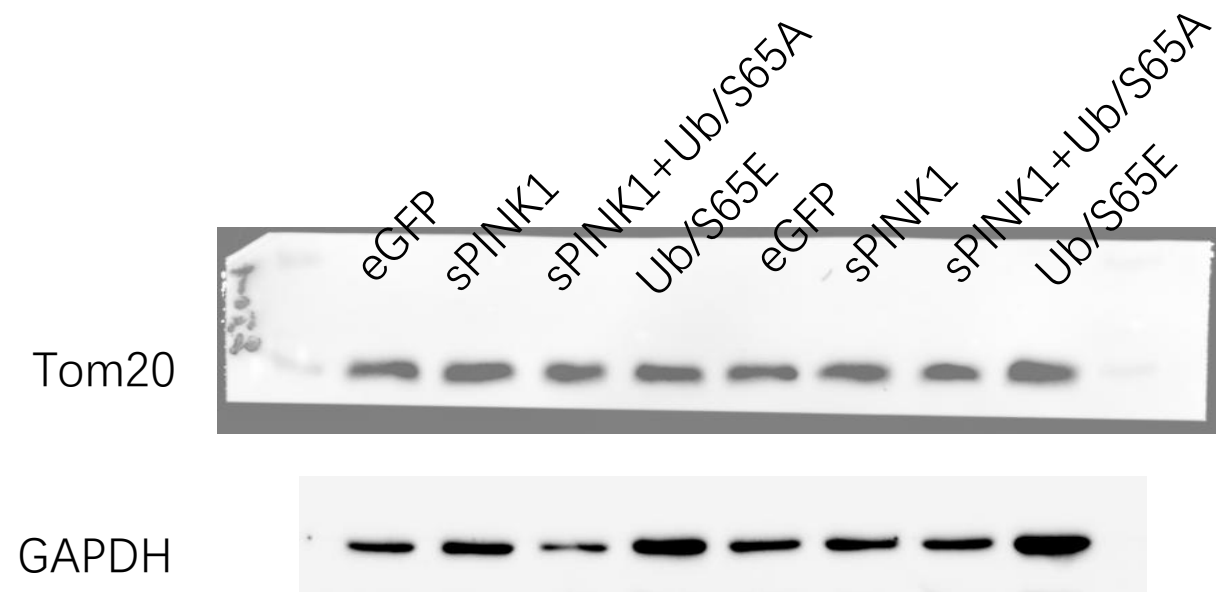

Supplement: Figure 7—source data 1. [file elife-103945-fig7-data1.pdf]

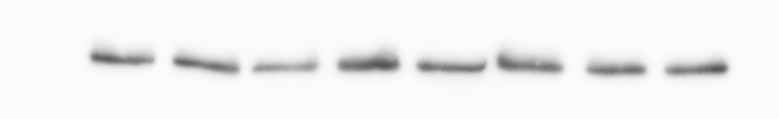

Supplement: Figure 7—source data 2. [file elife-103945-fig7-data2.zip › GAPDH-1.tif]

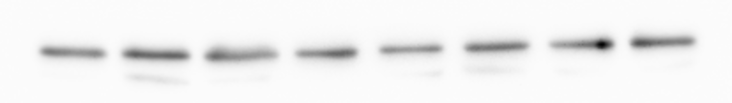

Supplement: Figure 7—source data 2. [file elife-103945-fig7-data2.zip › GAPDH-2.tif]

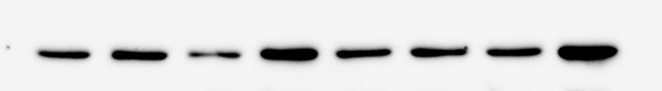

Supplement: Figure 7—source data 2. [file elife-103945-fig7-data2.zip › GAPDH-3.tif]

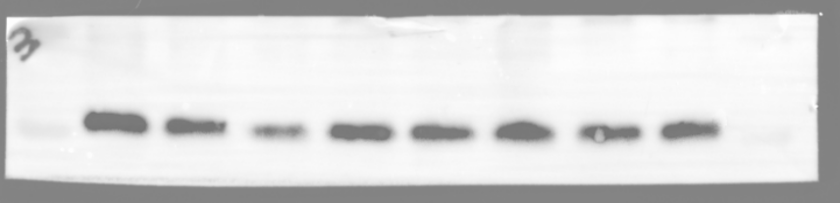

Supplement: Figure 7—source data 2. [file elife-103945-fig7-data2.zip › Tom20-1.tif]

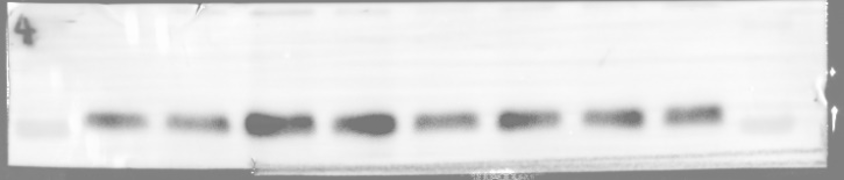

Supplement: Figure 7—source data 2. [file elife-103945-fig7-data2.zip › Tom20-2.tif]

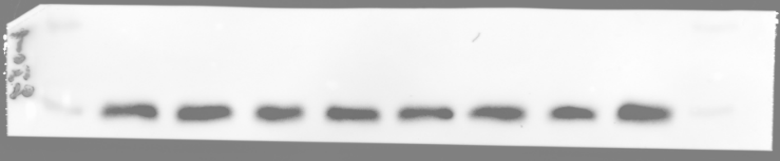

Supplement: Figure 7—source data 2. [file elife-103945-fig7-data2.zip › Tom20-3.tif]

MAP2

GAPDH

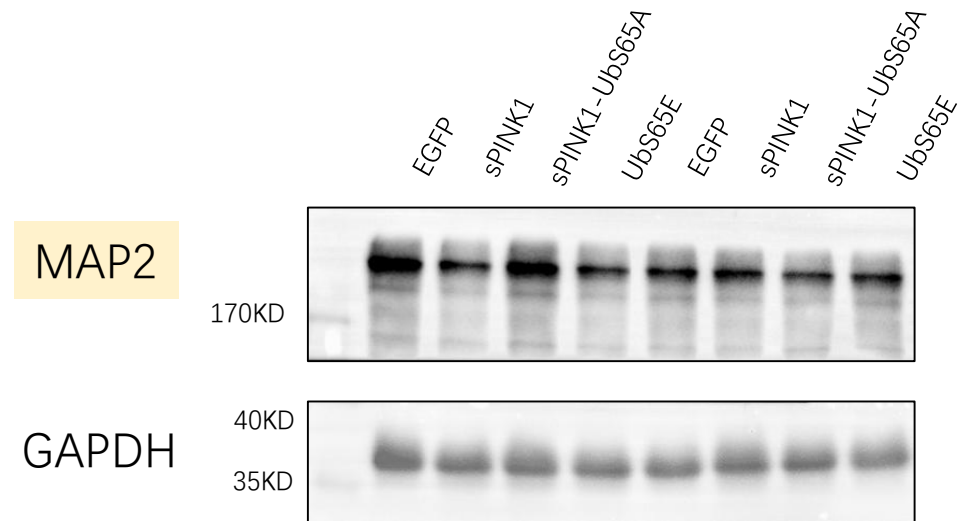

MAP2

GAPDH

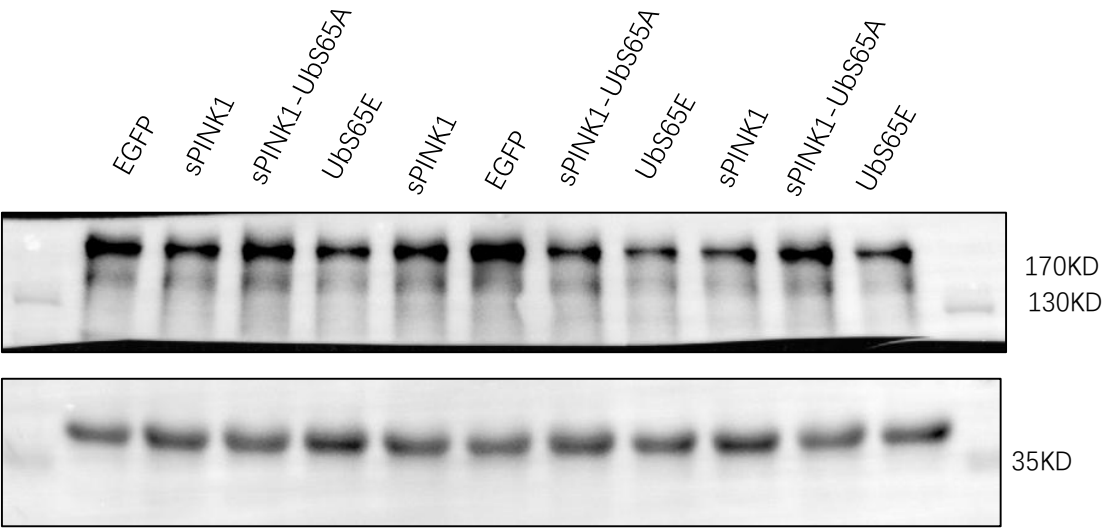

Supplement: Figure 7—source data 3. [file elife-103945-fig7-data3.pdf]

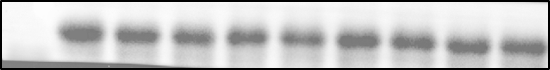

Supplement: Figure 7—source data 4. [file elife-103945-fig7-data4.zip › GAPDH-1.tif]

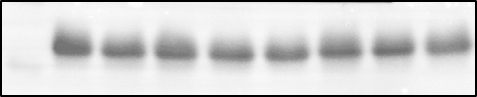

Supplement: Figure 7—source data 4. [file elife-103945-fig7-data4.zip › GAPDH-2.tif]

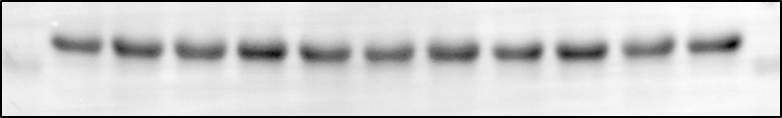

Supplement: Figure 7—source data 4. [file elife-103945-fig7-data4.zip › GAPDH-3.tif]

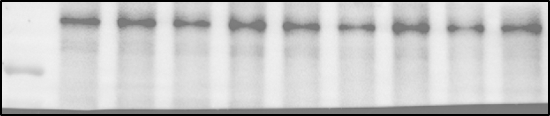

Supplement: Figure 7—source data 4. [file elife-103945-fig7-data4.zip › MAP2-1.tif]

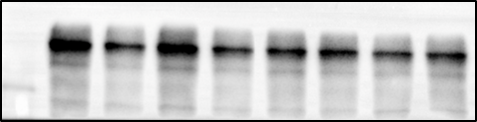

Supplement: Figure 7—source data 4. [file elife-103945-fig7-data4.zip › MAP2-2.tif]

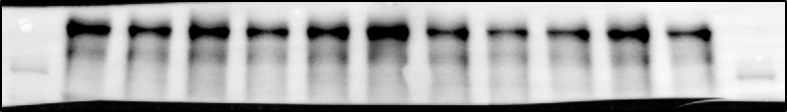

Supplement: Figure 7—source data 4. [file elife-103945-fig7-data4.zip › MAP2-3.tif]

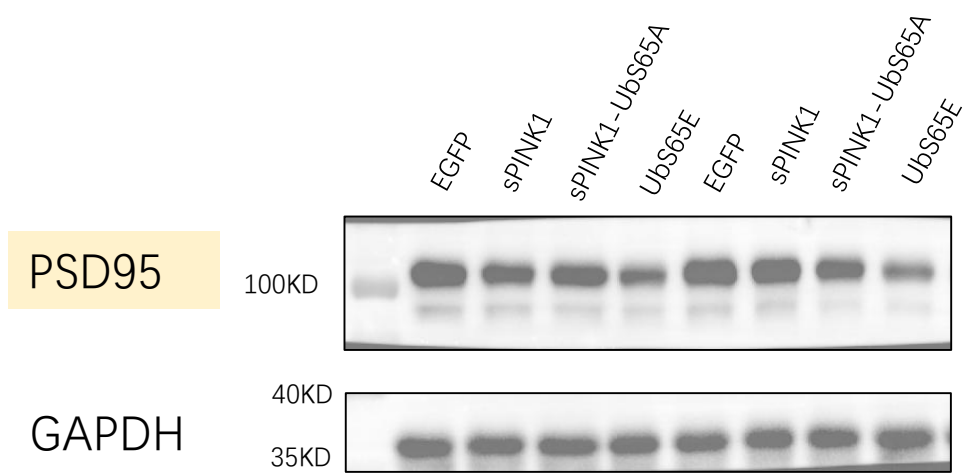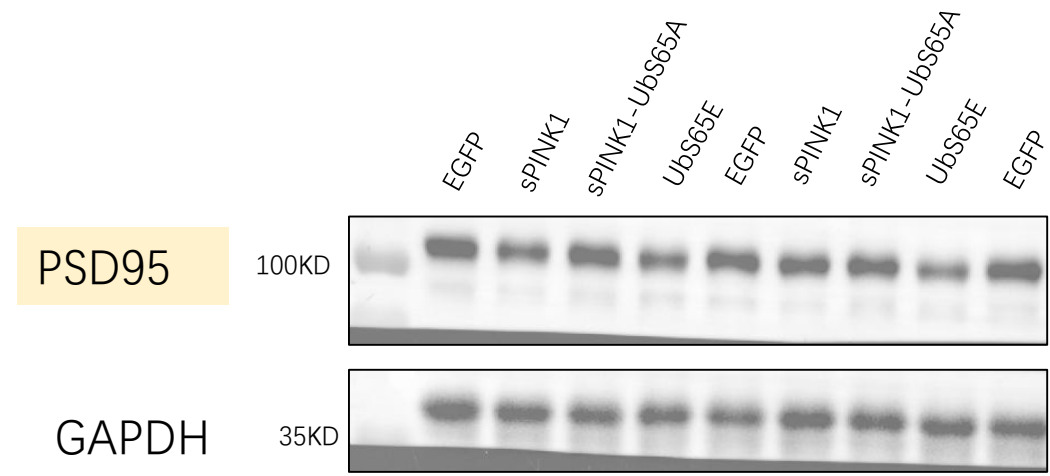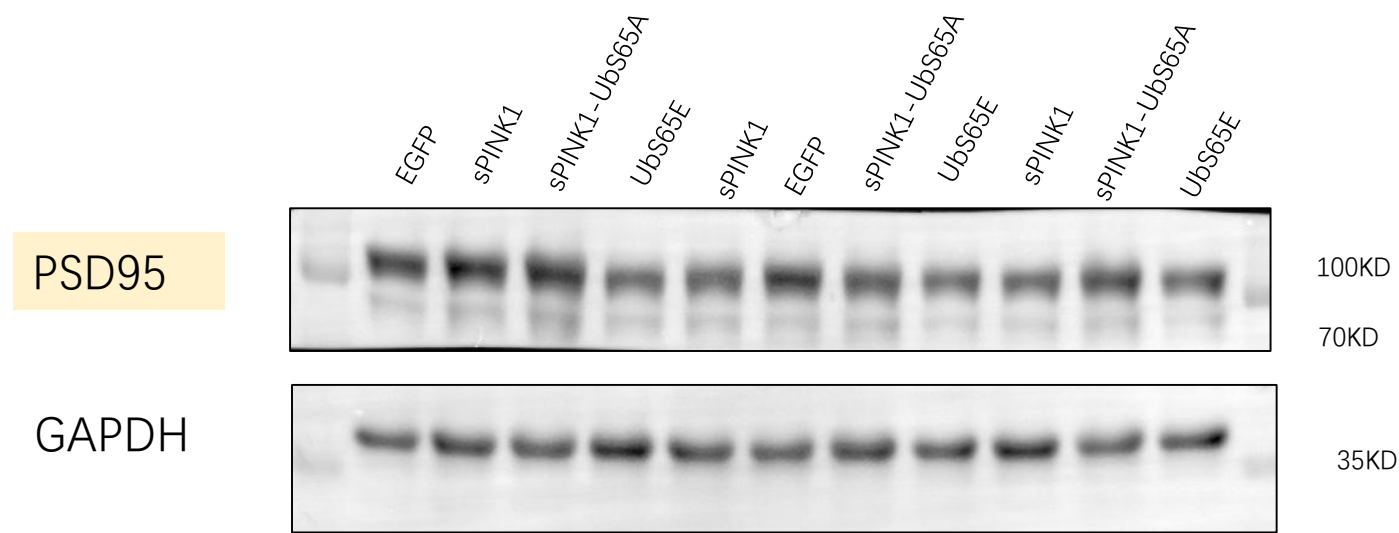

Supplement: Figure 7—source data 5. [file elife-103945-fig7-data5.pdf]

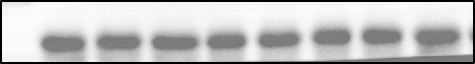

Supplement: Figure 7—source data 6. [file elife-103945-fig7-data6.zip › GAPDH-1.tif]

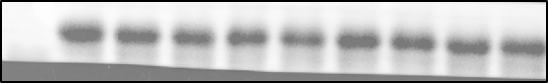

Supplement: Figure 7—source data 6. [file elife-103945-fig7-data6.zip › GAPDH-2.tif]

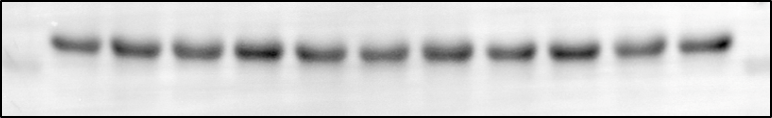

Supplement: Figure 7—source data 6. [file elife-103945-fig7-data6.zip › GAPDH-3.tif]

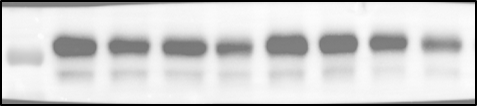

Supplement: Figure 7—source data 6. [file elife-103945-fig7-data6.zip › PSD95-1.tif]

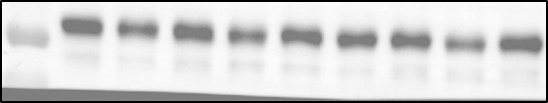

Supplement: Figure 7—source data 6. [file elife-103945-fig7-data6.zip › PSD95-2.tif]

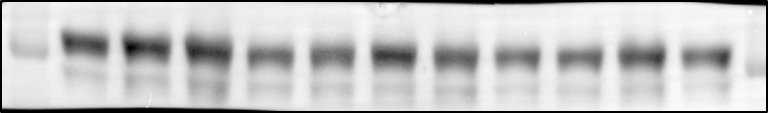

Supplement: Figure 7—source data 6. [file elife-103945-fig7-data6.zip › PSD95-3.tif]

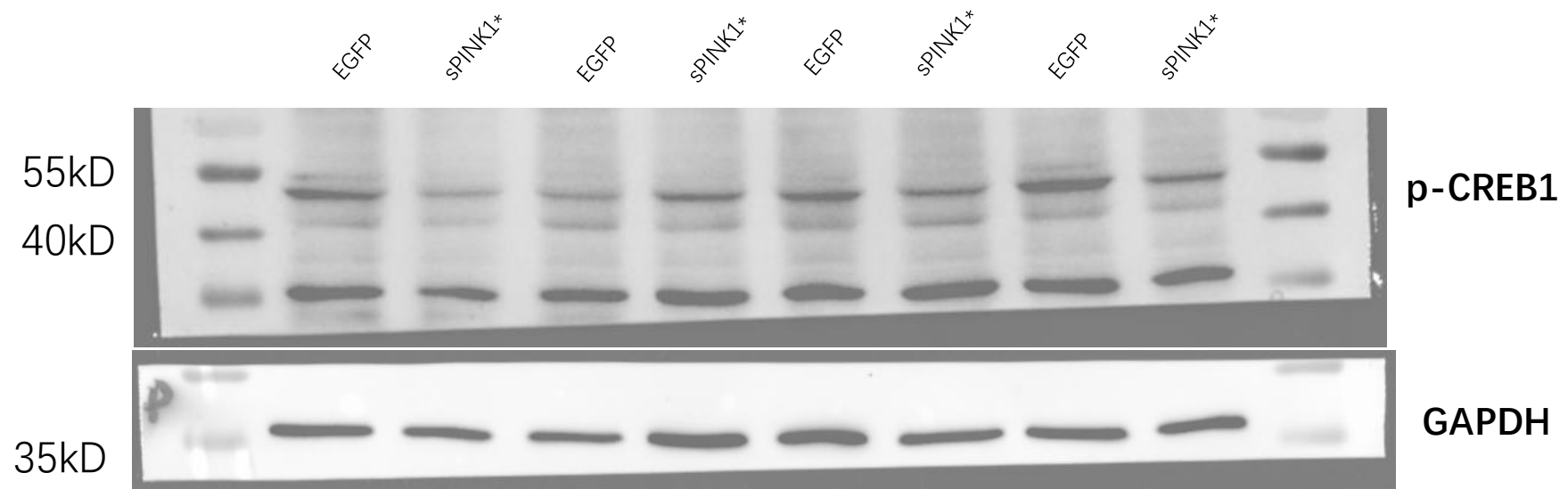

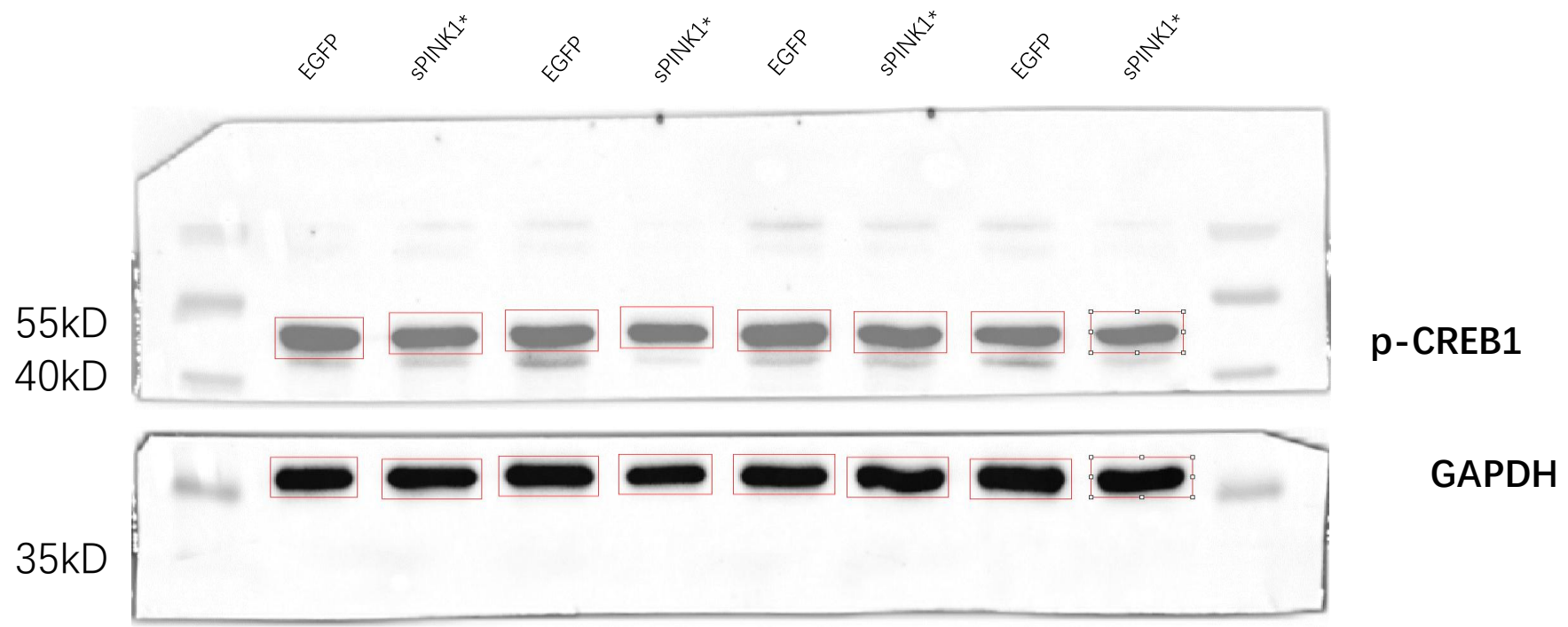

Supplement: Figure 8—source data 1. [file elife-103945-fig8-data1.pdf]

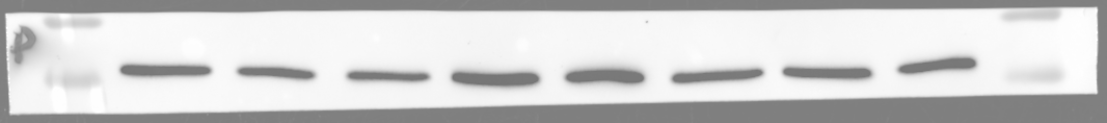

Supplement: Figure 8—source data 2. [file elife-103945-fig8-data2.zip › GAPDH 1.tif]

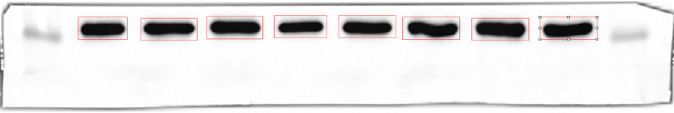

Supplement: Figure 8—source data 2. [file elife-103945-fig8-data2.zip › GAPDH 2.tif]

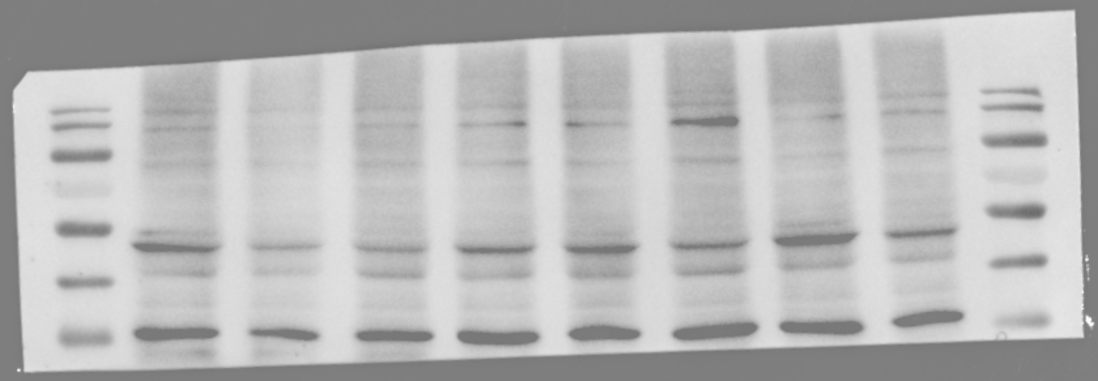

Supplement: Figure 8—source data 2. [file elife-103945-fig8-data2.zip › p-CREB 1.tif]

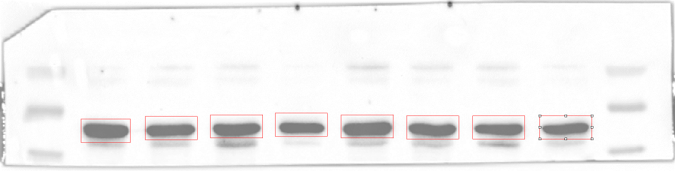

Supplement: Figure 8—source data 2. [file elife-103945-fig8-data2.zip › p-CREB 2.tif]
