# Supplementary material for: Elevated ubiquitin phosphorylation by PINK1 contributes to proteasomal impairment and promotes neurodegeneration
Source: eLife. 2025 Jul 31;14:RP103945. doi: 10.7554/eLife.103945 (PMC12313235; doi:10.7554/eLife.103945)
Supplement: Figure 6—source data 9. [file elife-103945-fig6-data9.pdf]

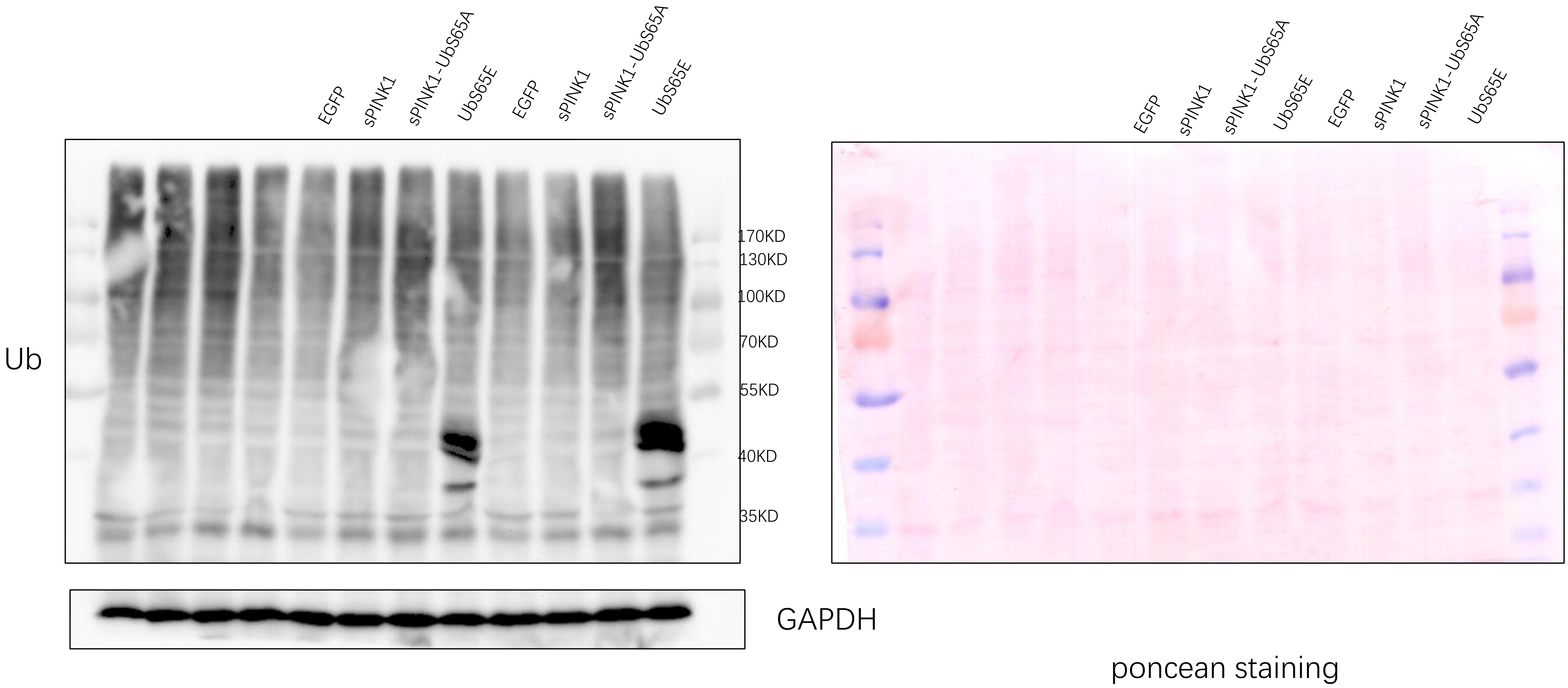
EGFP
sPINK1
sPINK1-UbS65A
UbS65E
EGFP
sPINK1
sPINK1-UbS65A
UbS65E
170KD
130KD
100KD
70KD
55KD
40KD
35KD
Ub
GAPDH
EGFP
sPINK1
sPINK1-UbS65A
UbS65E
EGFP
sPINK1
sPINK1-UbS65A
UbS65E
poncean staining

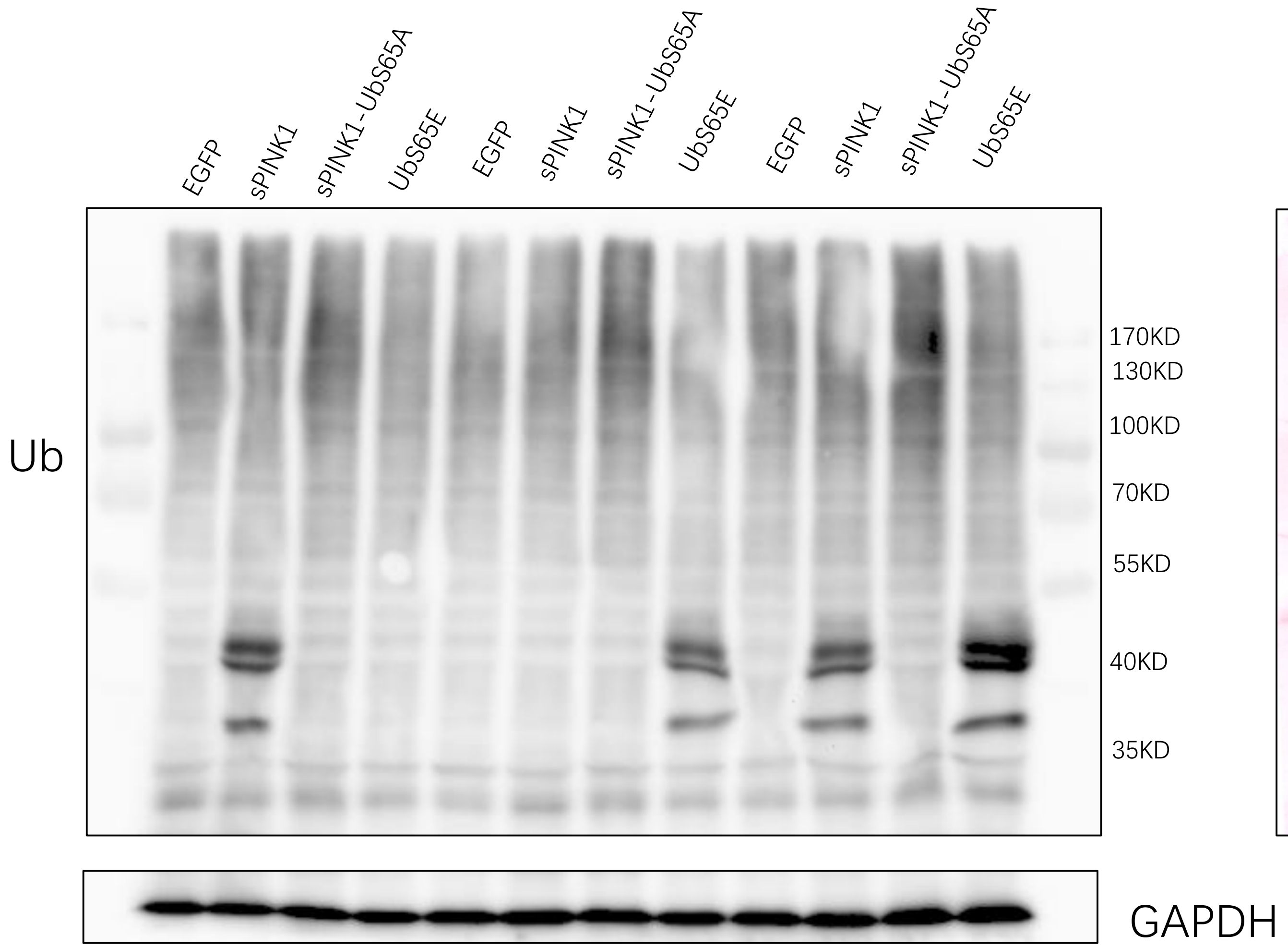

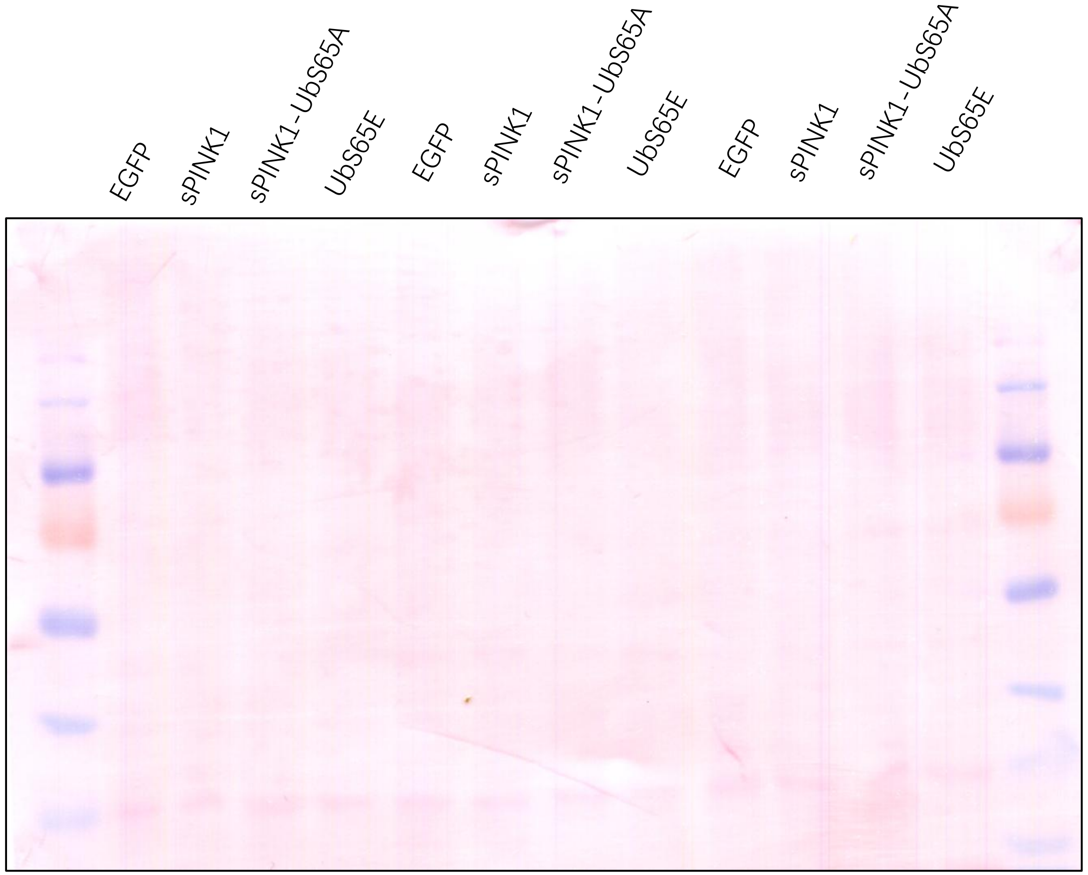

poncean staining

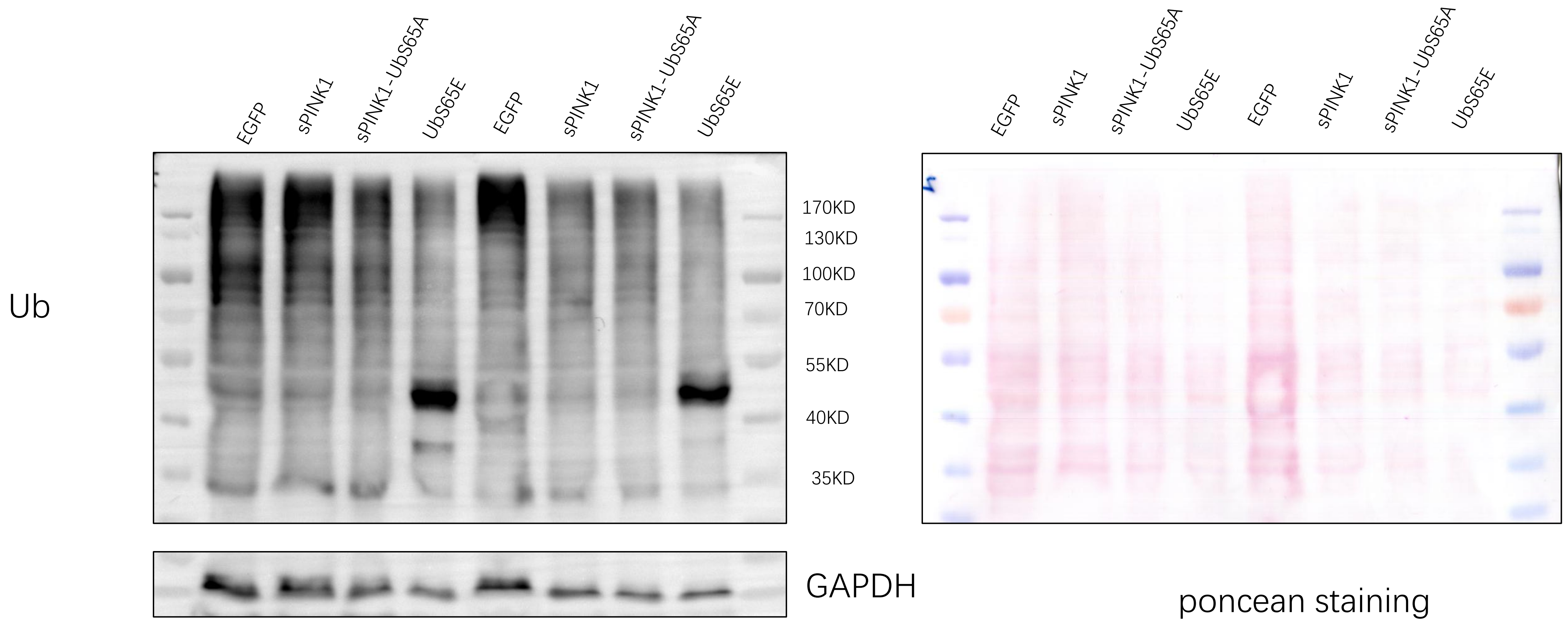

Ub
EGFP
sPINK1
sPINK1-UbS65A
UbS65E
EGFP
sPINK1
sPINK1-UbS65A
UbS65E
170KD
130KD
100KD
70KD
55KD
40KD
35KD
GAPDH
EGFP
sPINK1
sPINK1-UbS65A
UbS65E
EGFP
sPINK1
sPINK1-UbS65A
UbS65E
poncean staining
