# Supplementary material for: Elevated ubiquitin phosphorylation by PINK1 contributes to proteasomal impairment and promotes neurodegeneration
Source: eLife. 2025 Jul 31;14:RP103945. doi: 10.7554/eLife.103945 (PMC12313235; doi:10.7554/eLife.103945)

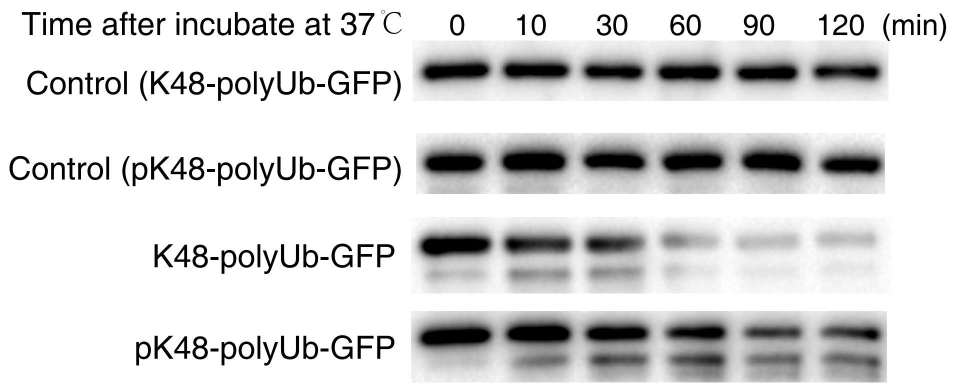

anti-GFP

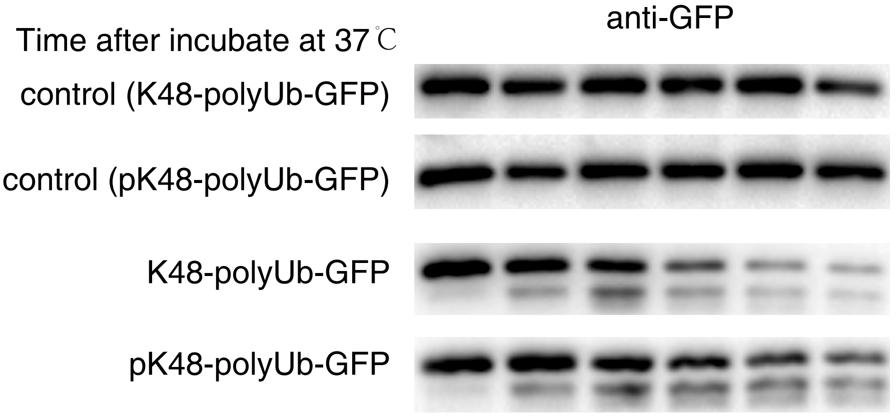

anti-GFP

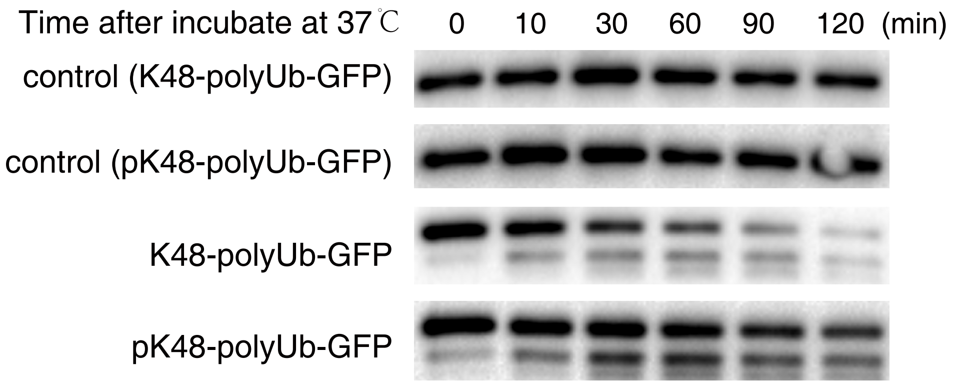

Supplement: Figure 3—source data 1. [file elife-103945-fig3-data1.pdf]
